# Metabolomics Analysis Reveals Deranged Energy Metabolism and Amino Acid Metabolic Reprogramming in Dogs With Myxomatous Mitral Valve Disease

**DOI:** 10.1161/JAHA.120.018923

**Published:** 2021-04-23

**Authors:** Qinghong Li, Éva Larouche‐Lebel, Kerry A. Loughran, Terry P. Huh, Jan S. Suchodolski, Mark A. Oyama

**Affiliations:** ^1^ Nestlé Purina Research St. Louis MO; ^2^ Department of Clinical Sciences and Advanced Medicine School of Veterinary Medicine University of Pennsylvania Philadelphia PA; ^3^ Gastrointestinal Laboratory Department of Small Animal Clinical Sciences College of Veterinary Medicine and Biomedical Sciences Texas A&M University College Station TX

**Keywords:** amino acids, canine, congestive heart failure, dog, energy metabolism, heart failure, metabolomics, mitral valve, mitral valve regurgitation, uremic toxin, Metabolism, Basic Science Research, Heart Failure, Valvular Heart Disease, Biomarkers

## Abstract

**Background:**

Myxomatous mitral valve disease (MMVD), a naturally occurring heart disease, affects 10% to 15% of the canine population. Canine MMVD shares many similarities with human MMVD. Untargeted metabolomics was performed to identify changes in metabolic pathways and biomarkers with potential clinical utilities.

**Methods and Results:**

Serum samples from 27 healthy, 22 stage B1, 18 stage B2 preclinical MMVD dogs, and 17 MMVD dogs with a history of congestive heart failure (CHF) were analyzed. Linear regression analysis identified 173 known metabolites whose concentrations were different among the 4 groups (adjusted *P*<0.05), of which 40% belonged to amino acid super pathways, while 30% were lipids. More than 50% of significant metabolites were correlated with left atrial diameter but not left ventricular dimension. Acylcarnitines, tricarboxylic acid cycle intermediates, and creatine accumulated in proportion to MMVD severity. α‐Ketobutyrate and ketone bodies were increased as MMVD advanced. Nicotinamide, a key substrate of the main nicotinamide adenine dinucleotide (NAD^+^) salvage pathway, was decreased, while quinolinate of the de novo NAD^+^ biosynthesis was increased in CHF dogs versus healthy dogs. 3‐Methylhistidine, marker for myofibrillar protein degradation, was higher in CHF dogs than non‐CHF dogs. Trimethylamine N‐oxide (TMAO) and TMAO–producing precursors, including carnitine, phosphatidylcholine, betaine, and trimethyllysine, were increased in CHF dogs versus non‐CHF dogs. Elevated levels of uremic toxins, including guanidino compounds, TMAO, and urea, were observed in CHF dogs. Pathway analysis highlighted the importance of bioenergetics and amino acid metabolism in canine MMVD.

**Conclusions:**

Our study revealed altered energy metabolism, amino acid metabolic programming, and reduced renal function in the development of MMVD and CHF. Complex interplays along the heart‐kidney‐gut axis were implicated.

**Nonstandard Abbreviations and Acronyms**
3‐MH: 3‐methylhistidine
BCS: body condition score
BHBA: 3‐hydroxybutyrate/β‐hydroxybutyrate
FC: fold‐change
FDR: false discovery rate
KEGG: Kyoto Encyclopedia of Genes and Genomes
MetPA: Metabolomics pathway analysis
MMVD: myxomatous mitral valve disease
NAD^+^: nicotinamide adenine dinucleotide
nLAD: normalized left atrial diameter
PC: principal component
PCA: principal component analysis
qMSEA: quantitative metabolite set enrichment analysis
TCA: tricarboxylic acid
TMAO: trimethylamine N‐oxide
TMAP: N,N,N‐trimethyl‐L‐alanyl‐L‐proline betaine
TMAVA: N,N,N‐trimethyl‐5‐aminovalerate

**Clinical Perspective**

**What Is New?**

- We reported the first untargeted serum metabolomic analysis comparing all stages of naturally occurring myxomatous mitral valve disease in dogs.

**What Are the Clinical Implications?**

- Eighty‐two differential metabolites were identified between healthy dogs and dogs with preclinical stage B1 myxomatous mitral valve disease, while 12 were identified between B1 and B2 stages, and the results may offer novel insights into early transition, development of volume overload, and diagnostic potentials.
- Canine myxomatous mitral valve disease is considered a model for human myxomatous mitral valve disease, and information gained in this study may have clinical relevance for human patients.

Myxomatous mitral valve disease (MMVD), the most common naturally occurring heart disease in dogs, is characterized by progressive valvular degeneration that can cause mitral regurgitation and lead to congestive heart failure (CHF).^1^ MMVD affects 10% to 15% of the canine population, with a greater frequency in small‐ and medium‐breed geriatric dogs.^2^ A staging scheme for classifying canine MMVD has been adopted by the consensus committee established by the American College of Veterinary Internal Medicine.^3^ Dogs at risk for developing MMVD but otherwise healthy are considered stage A; dogs with a heart murmur caused by mitral regurgitation but no clinical signs of CHF are classified as stage B; dogs with MMVD and overt clinical signs of CHF are classified as stage C; and MMVD dogs with CHF refractory to treatment are classified as stage D. Stage B dogs are further classified into B1 or B2 based on the absence or presence of cardiac remodeling, respectively. In general, MMVD dogs have a lengthy preclinical stage B. Once progressed to late stage B2 and stage C, the disease advances more rapidly, with a mean survival time of <12 months in dogs with CHF.^4^

Metabolomics has been increasingly used to interrogate molecular and metabolic changes in cardiovascular diseases and is regarded as one of the signposts on the path to clinical utility.^5^, ^6^, ^7^ Metabolomic analysis combined with other systems approaches provides an opportunity to investigate the mechanism of disease development and progression.^8^ The metabolic machinery of cardiac energy metabolism includes 3 interconnected components: substrate utilization, oxidative phosphorylation, and energy transfer to and utilization by myofibrils.^9^ A wealth of evidence supports a link between energy substrate metabolism and cardiac functions.^9^, ^10^ In healthy heart, the main energy substrate used by myocardial cells is fatty acids (FAs). The failing heart experiences reduced capacity of mitochondrial FA oxidation as the main energy source and increases its reliance on alternative energy substrates such as ketone bodies and glucose.^11^, ^12^, ^13^, ^14^, ^15^ In recent years, other gut microbe–related pathways have been associated with pathogenesis of cardiovascular diseases including heart failure (HF) in humans.^16^, ^17^ For instance, circulating levels of trimethylamine N‐oxide (TMAO), a gut microbial metabolite, were linked to increased risks of major adverse cardiovascular events in humans and MMVD in dogs.^18^, ^19^, ^20^

To date, there are only 2 metabolomic profiling studies on canine MMVD. One study compared serum samples between 18 healthy dogs and 11 age‐ and sex‐matched dogs with preclinical MMVD, while the other reported changes in serum metabolome in response to diet intervention in dogs with preclinical MMVD.^21^, ^22^ Direct comparisons in metabolome between various stages of MMVD are lacking. Metabolomic study of human MMVD is also scarce, with only 1 published investigation, which reported differential metabolites in affected versus healthy patients.^23^ Canine MMVD is considered a model for human MMVD in that they share many similarities at the molecular and pathophysiological levels,^23^, ^24^, ^25^, ^26^, ^27^, ^28^ and information gained from canine studies may therefore have relevance in human patients. Our hypothesis was that serum metabolomics changes reflect adaptations of energy substrates and interruptions of energy metabolic machinery during MMVD progression, and that gut microbial mediators and uremic toxins are associated with stage B2 or C/D MMVD. Our goal was to further our understanding of MMVD pathogenesis at the molecular and systemic levels, to identify future therapeutic targets and new biomarkers with diagnostic and prognostic potential through cross‐sectional comparisons.

## Materials and Methods

The authors declare that all of the supporting data are available as Data S1 and S2.

### Animals and Study Approval

The study protocol was reviewed and approved by the University of Pennsylvania Institutional Animal Care and Use Committee, and informed owner consent was obtained. Clinically healthy dogs without a heart murmur and without concurrent systemic disease were prospectively enrolled as controls (group A). This group of dogs primarily consisted of systemically healthy dogs owned by students and staff of the hospital. A cohort of dogs with a left apical systolic murmur, echocardiographic diagnosis of thickened and prolapsing mitral valve leaflet(s), and mitral regurgitation, as well as clinical history and physical examination consistent with stage B1, B2, C, or D MMVD were considered for group B1, group B2, and group C/D, respectively.^3^ Any dog with severe concurrent systemic disease including diabetes mellitus, cancer, or renal failure, or those with any congenital heart disease, were excluded.

### Serum Sample Collection

Venous blood samples of 2 to 3 mL were collected in plain red‐topped tubes. The blood was allowed to clot and centrifuged at 1600*g* for 5 minutes to yield serum samples, which were stored at −80°C until use.

### Echocardiography

Echocardiographic studies (iE33, Philips Healthcare) were performed without sedation. Left ventricular internal dimensions in end‐diastole and left ventricular internal dimensions in end‐systole, normalized left atrial diameter (nLAD), and normalized aortic root diameter were measured from right parasternal short‐axis 2‐dimensional images and normalized to body weight.^29^ The ratio of the left atrial diameter to the aortic root diameter was calculated.

### Metabolomics Assay

Untargeted metabolomics assays were performed at a commercial laboratory (Metabolon, Inc.). Sample preparation and extraction, liquid chromatography, and mass spectrometry followed Metabolon standard protocols as previously described (Supplemental Methods).^22^, ^30^ Compound detection and identification were performed using Metabolon proprietary software and database. A total of 1033 metabolites were identified, including 912 known and 121 unknown (Data S1).

### Data Processing

The raw data were generated based on the area‐under‐the‐curve formula using ion counts that provide relative quantification (Data S2). Metabolites with missing values in >80% of samples were removed. The remaining missing data were imputed with a value equal to half of the minimal value in the raw data under the assumption that missing data were those below the detection limit. Metabolites that comprised the bottom 25th percentile in the interquartile range represented near‐constant values and were removed. The data were further transformed using the logarithm to the base 2, and autoscaled to achieve a zero mean and unit variance for all metabolites.

### Statistical Analysis

Principal component (PC) analysis (PCA) for high‐dimensional multivariate data was performed using the R function “prcomp” and PCs were calculated. The first 2 PCs, PC1 and PC2, which capture more data variations than other PCs, were examined for their ability to separate the 4 groups. A multiple linear regression using PC1 or PC2 as the dependent variable and group as the independent variable, adjusted for age, body condition score (BCS), and body weight was performed. Tukey's post hoc test was performed to compare the means between groups and *P* values were adjusted for multiple testing error.

To identify differential metabolites, a multiple linear regression, adjusted for age, BCS, and body weight was performed. *P* values were adjusted to control the false discovery rate (FDR) using the Benjamini‐Hochberg method. Significant metabolites were subjected to pairwise comparisons using the R function “pairwise.t.test” with pooled SD and Benjamini‐Hochberg adjustment for multiple testing. FDR ≤0.05 was considered significant.

To compare the means of continuous variables, ANOVA and Tukey's post hoc tests were performed. To test the null hypothesis that the 2 categorical variables were independent, chi‐square test was performed if all expected numbers were >5. Otherwise, a Fisher's exact test was used instead. Fold‐change (FC) was defined as the ratio of the mean of group 2 (g2) over that of group 1 (g1): ${\mathrm{FC}=2}^{\left(\log_{2}g2‐\log_{2}g1\right)}$.

Pearson's correlation coefficients between echocardiographic variables and significant metabolites were calculated. The 8 dogs without echocardiographic measurements from group A were excluded from the analysis. *P* values were adjusted for multiple testing errors using the Benjamini‐Hochberg method. Adjusted *P*≤0.05 was considered significant. Pairwise Pearson's correlation analysis among significant metabolites was also performed.

### Analysis of Confounding Effects

To account for any potential confounding effect from age or body weight, 500 iterations of bootstrap resampling without replacement were performed where 10 samples from each group were randomly selected. Those bootstrapped subsamples with no difference in age or body weight were selected for further analysis. PCA and multiple linear regression were performed on each subsample set using PC1 or PC2 as the dependent variable and group as the explanatory variable, adjusted for age, BCS, and sex. *P* value distributions were analyzed.

Potential confounding effect from cardiac medications was also evaluated using data from groups B1 and B2 dogs. Dogs taking ≥1 of 4 commonly used cardiac medications, pimobendan, furosemide, angiotensin‐converting enzyme inhibitors, and spironolactone, for at least 2 weeks before the time of sampling were considered for group Y, while the rest of the dogs were considered for group N. PCA and Student *t* test were performed to test the null hypothesis that the means of PC1 or PC2 between group Y and group N were not different.

### Metabolomics Pathway Analysis and Quantitative Metabolite Set Enrichment Analysis

Metabolomics pathway analysis (MetPA) was performed using the Human Metabolome Database IDs of the significant metabolites.^31^ The Kyoto Encyclopedia of Genes and Genomes (KEGG) pathway library for *homo sapiens* was searched and pathway overrepresentation analysis was performed using hypergeometric test. The pathway impact was calculated as the sum of the importance measures of the matched metabolites normalized by the sum of the importance measures of all metabolites in each pathway. Quantitative metabolite set enrichment analysis (qMSEA) was performed to identify enriched metabolite sets between group C/D and group A using the PubChem IDs and the concentration data of all metabolites.^32^ Metabolite sets with at least 2 compounds in the Small Molecule Pathway Database (www.smpdb.ca) was searched. Pathways with *P*≤0.05 were considered significant.

Data processing and statistical analysis were performed in the statistical computing software R (version 3.5.0). Partial least square discriminant analysis, MetPA and qMSEA were performed in MetaboAnalyst 4.0.^33^

## Results

Eighty‐four client‐owned dogs, including 27 group A, 22 group B1, 18 group B2, and 17 group C/D dogs, were enrolled in the study (Table). Significant differences in age and body weight were found (both *P*_ANOVA_<0.001). Dogs in group A were significantly younger than dogs in any other group, and had a greater mean body weight than those in groups B2 and C/D (adjusted *P*<0.05 in all comparisons, Figure S1). No difference was found in sex or BCS.

There were significant differences in left ventricular internal dimensions in end‐diastole, nLAD, and ratio of the left atrial diameter to the aortic root diameter (*P*_ANOVA_<0.0001, Table), where differences were found in all pairwise group comparisons (adjusted *P*<0.05) except between groups A and B1 (Figure S1). No difference was observed in left ventricular internal dimensions in end‐systole or normalized aortic root diameter.

### Global Metabolome Changes

**Figure 1:**
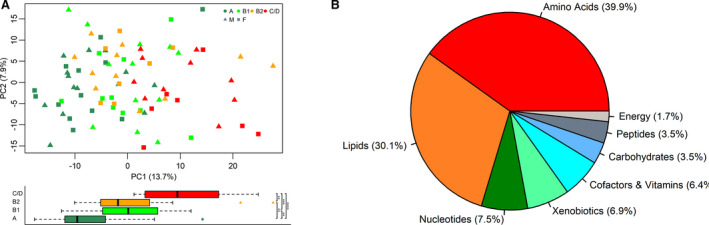
Principal component analysis (PCA) and differential metabolites. The PCA plot (**A**, top panel) and boxplots along the first principal component (PC1) (**A**, bottom panel). The percentages of data variation explained by PC1 and PC2 are indicated on the x and y axes, respectively. Tukey tests were performed to compare between groups. *P* values were adjusted for multiple testing. **P<0.01; ***P<0.001; ****P<0.0001. **B**, The pie chart shows percentages of classes of the 173 known metabolites. F indicates female; and M, male.

PCA showed clear separation of the 4 groups along PC1 (*P*=1.93×10^−11^; Figure 1A, top panel), but not along PC2 (*P*=0.18). Differences were found in all pairwise group comparisons (adjusted *P*<0.01; Figure 1A, lower panel) except between groups B1 and B2. In partial least square discriminant analysis, separations were evidenced along component 1 and component 2 (*P*=3.2×10^−16^ and 1.1×10^−5^ respectively, Figure S2).

Multiple linear regression analysis identified 201 significant metabolites, of those 173 of 201 (86.1%) were known (Table S1). Sixty‐nine metabolites (69/173) belonged to amino acid super pathways, while 52 of 173 (30.1%) were lipids (Figure 1B).

### Analysis of Confounding Effects

In 1 computational simulation, 500 bootstrap experiments generated 177 subsamples with no difference in age or body weight (*P*>0.05 in both cases, Table S2). Among those, 17 (17/177) had a significant difference in BCS but none (0/177) had a sex effect. The minimum, median, and maximum of the *P* values for PC1 were 1.7×10^−7^, 2.5×10^−4^, and 0.02, respectively (Figure S3A).

Forty dogs from group B1 and B2 were analyzed for potential effects of cardiac medications. No difference in PC1 or PC2 was found between group Y and group N (*P*=0.38, 0.69 respectively; Figure S3B).

### Energy Metabolism

**Figure 2:**
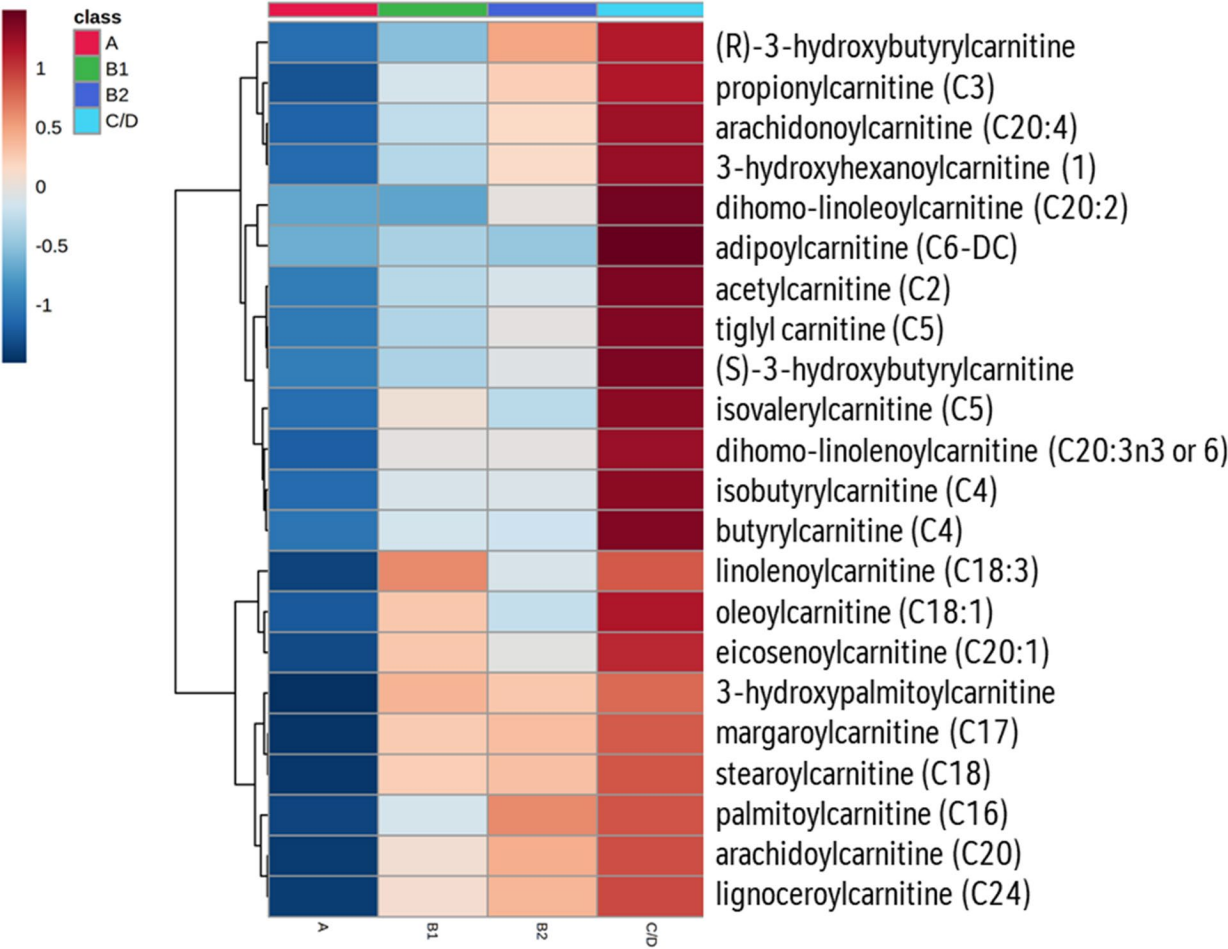
Heat map of 22 significant acylcarnitines. Hierarchical clustering on the features was performed using the Euclidean distances calculated from the group means. The color scale corresponds to concentrations in log scale from low (deep blue) to high (maroon).

**Figure 3:**
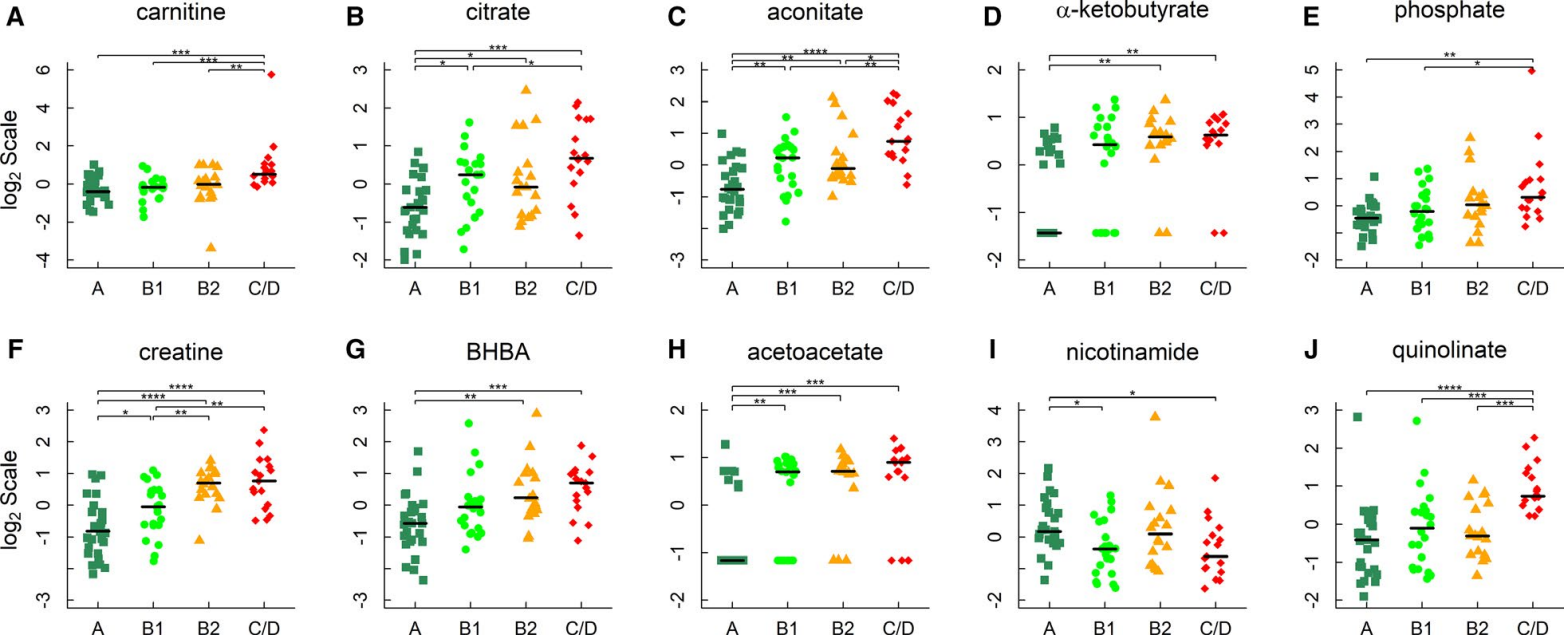
Metabolites in energy metabolism. **A** through **F**, Carnitine, citrate, aconitate, α‐ketobutyrate, phosphate, and creatine; (**G** and **H**) 3‐hydroxybutyrate/β‐hydroxybutyrate (BHBA) and acetoacetate; and (**I** and **J**) nicotinamide and quinolinate. Horizontal lines denote medians. The metabolites were identified by linear regression analyses and subject to post hoc comparisons with corrections for multiple testing. Adjusted *P* values: *P<0.05; **P<0.01; ***P<0.001; ****P<0.0001.

Metabolites in all 3 components of the energy metabolic machinery, including energy substrate transfer, oxidative phosphorylation, and high‐energy phosphate bond transfer and utilization, were examined. Significant changes were found in 22 acylcarnitines, all of which showed >1.8‐fold increases in group C/D compared with group A, while 11of 22 (50%) and 8 of 22 (36.4%) of the acylcarnitines showed >1.5‐fold increases in group C/D versus group B1 or B2, respectively (FDR<0.05 in all cases; Figure 2, Figure S4). Carnitine concentration was increased by >2‐fold in group C/D versus any other group (Figure 3A).

Citrate and aconitate, 2 intermediates of the tricarboxylic acid (TCA) cycle, were increased in groups B1, B2, and C/D versus group A, and in group C/D versus group B1 (FC>1.5 in all cases, Figure 3B and 3C). The level of α‐ketobutyrate, a precursor of succinyl‐coenzyme A, was higher in groups B2 and C/D than that of group A (FC>1.9 in all cases, Figure 3D). Inorganic phosphate, which is taken up by ADP to form ATP in the TCA cycle, was increased in group C/D compared with group A or B1 (FC>1.8 in both cases, Figure 3E). The level of creatine, a principal component of the creatine kinase energy shuttle system, was also elevated with MMVD progression (FC>1.5 in all cases, Figure 3F).

Significant changes in ketone bodies were observed: 3‐hydroxybutyrate/β‐hydroxybutyrate (BHBA) and acetoacetate were increased in both group B2 and group C/D compared with group A (FC≥2 in all cases, Figure 3G and 3H). Acetoacetate level was also higher in group B1 than that of group A (FC=1.7). An increase in BHBA was observed in group B1 versus group A, but it did not reach statistical significance after adjusting for multiple testing (FC=1.5, FDR=0.06).

Nicotinamide, the precursor for nicotinamide adenine dinucleotides (NAD^+^) salvage pathway, was decreased in group B1 and group C/D compared with group A (FC=1.6 in both cases, Figure 3I). The concentration of quinolinate, a key intermediate in the NAD^+^ de novo biosynthesis pathway from L‐tryptophan, was higher in group C/D than that of any other group (FC >2.0 in all cases, Figure 3J).

### Amino Acid Metabolism

**Figure 4:**
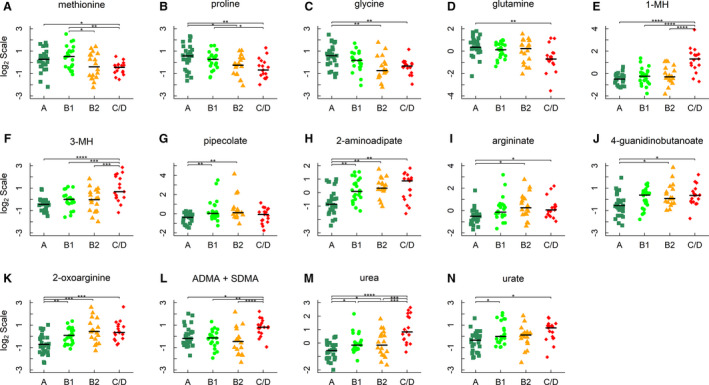
Amino acids, their derivatives, and uremic toxins. **A** through **D**, Methionine, proline, glycine, and glutamine; (**E** through **H**) 1‐methylhistidine (1‐MH), 3‐methylhistidine (3‐MH), pipecolate, and 2‐aminoadipate; and (**I** through **N**) argininate, 4‐guanidinobutanoate, 2‐oxoarginine, asymmetric and symmetric dimethylarginine (ADMA+SDMA), urea, and urate. Horizontal lines denote medians. The metabolites were identified by linear regression analyses and subject to post hoc comparisons with corrections for multiple testing. Adjusted *P* values: *P<0.05; **P<0.01; ***P<0.001; ****P<0.0001.

The concentrations of methionine, proline, glycine, and glutamine were decreased as MMVD progressed (FC >1.5 in all cases, Figure 4A through 4D), while the levels of 1‐methylhistidine and 3‐methylhistidine (3‐MH) were increased in group C/D compared with other groups (FC >2.0 in all cases, Figure 4E and 4F). Several compounds in the lysine degradation pathway were also changed. Pipecolate and 2‐aminoadipate, key intermediates of lysine degradation, were increased in groups B1 and B2 when compared with group A (FC >1.8 in all cases, Figure 4G and 4H). The level of 2‐aminoadipate was also higher in group C/D versus group A (FC=2.1, FDR=0.001).

The concentrations of several uremic toxins including TMAO were changed. The circulating levels of 3 guanidino compounds, including argininate, 2‐oxoarginate, and 4‐guanidinobutanoate, were higher in MMVD dogs compared with healthy dogs (Figure 4I through 4K). The level of asymmetric dimethylarginine, an endogenous inhibitor of nitric oxide synthase, was also higher in group C/D than that of other groups (Figure 4L). In addition, 2 nitrogenous waste products, urea and urate, were increased in MMVD dogs versus healthy dogs (Figure 4M and 4N).

### TMAO and Its Precursors

**Figure 5:**
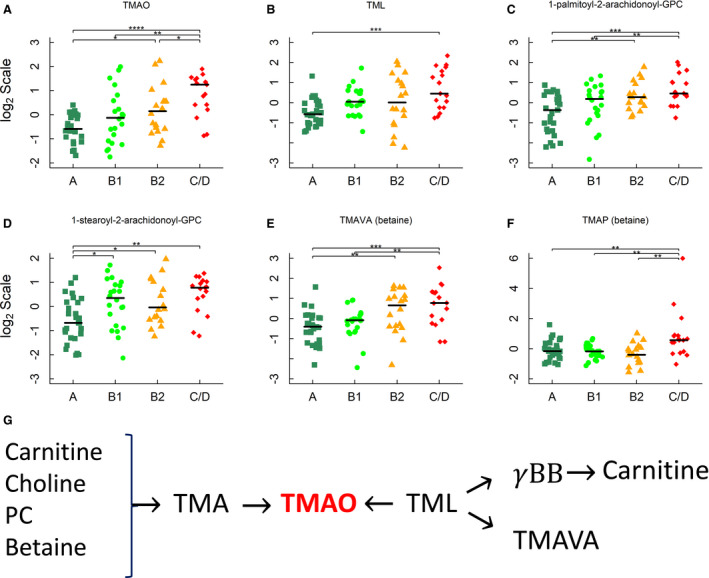
Trimethylamine N‐oxide (TMAO) and TMAO‐producing precursors. **A**, TMAO, (**B**) trimethyllysine, (**C** and **D**) 1‐palmitoyl‐arachidonoyl‐GPC and 1‐stearoyl‐2‐arachidonoyl‐GPC, and (**E** and **F**) N,N,N‐trimethyl‐5‐aminovalerate (also known as 5‐aminovaleric acid betaine) (TMAVA) or N,N,N‐trimethyl‐L‐alanyl‐L‐proline betaine (TMAP). **G**, Carnitine, choline, phosphatidylcholine, and betaine can be converted to trimethylamine by certain gut bacteria. Trimethylamine is released into circulation and converted to TMAO in the liver. Trimethyllysine is another precursor for microbial TMAO synthesis, as well as the substrate for γ‐butyrobetaine (γBB) and TMAVA synthesis. Horizontal lines denote medians. The metabolites were identified by linear regression analyses and subject to post hoc comparisons with corrections for multiple testing. Adjusted *P* values: *P<0.05; **P<0.01; ***P<0.001; ****P<0.0001.

The concentration of TMAO was higher in group C/D versus all other groups, and in group B2 versus group A (FC>1.5 in all cases, Figure 5A). Several TMAO‐producing nutrient precursors, including carnitine, trimethyllysine, phosphatidylcholines, and betaines, were also increased in MMVD dogs versus healthy dogs (Figures 3A and 5B through 5D). N,N,N‐trimethyl‐5‐aminovalerate (TMAVA, also known as 5‐aminovaleric acid betaine), another metabolite of intestinal microbes, was higher in groups B2 and C/D versus group A (Figure 5E). In addition, the concentration of N,N,N‐trimethyl‐L‐alanyl‐L‐proline betaine (TMAP), a novel marker for kidney function, was higher in group C/D than those of the other groups (FC>1.9 in all cases, Figure 5F).

### Correlation Analysis

**Figure 6:**
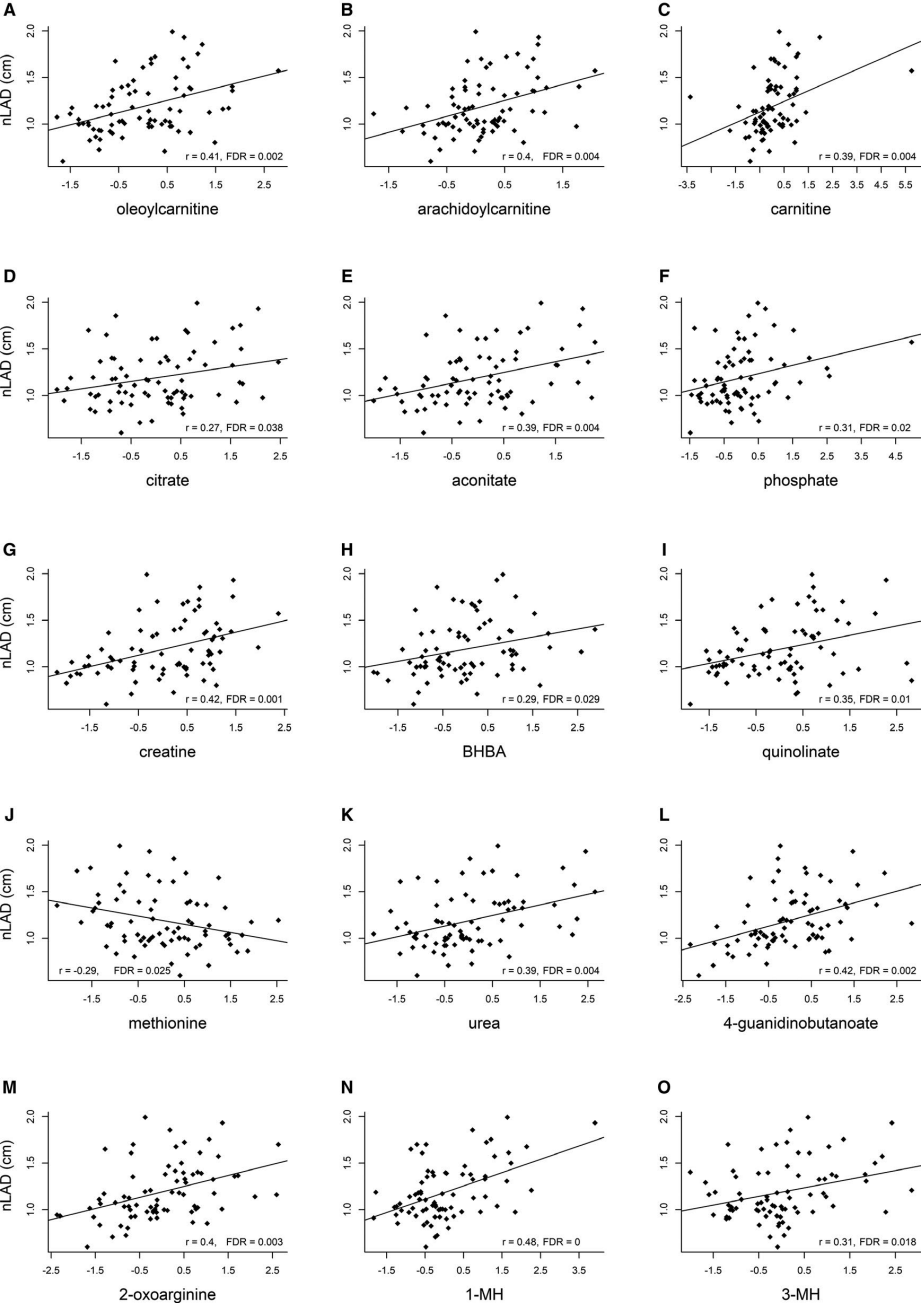
Pearson's correlations between normalized left atrial diameter (nLAD) and metabolites. **A** and **B**, Oleoylcarnitine and arachidoylcarnitine; (**C** through **J**) carnitine, citrate, aconitate, phosphate, creatine, 3‐hydroxybutyrate/β‐hydroxybutyrate (BHBA), quinolinate, and methionine, (**K** through **M**) urea, 4‐guanidinobutanoate, and 2‐oxoarginine, (**N** and **O**) 1‐methylhistidine (1‐MH) and 3‐methylhistidine (3‐MH). A fitted linear regression line, correlation coefficient (r), and adjusted *P* value (FDR) were included in each graph.

Among 173 known metabolites, 96 of 173 (55.5%) and 103 of 173 (59.5%) were significantly correlated with nLAD and ratio of the left atrial diameter to the aortic root diameter, respectively (FDR<0.05, Table S3). Of them, 88 of 173 (50.9%) were in common. In contrast, only 3 of 173 (1.7%) metabolites were correlated with left ventricular internal dimensions in end‐diastole. Correlations between nLAD and key metabolites from energy metabolism, and amino acid metabolisms are shown in Figure 6.

Aconitate and citrate of the TCA cycle had a near‐perfect correlation with each other (*r*=0.93, Figure S5A). Aconitate was also positively correlated with numerous metabolites including quinolinate, urea, BHBA, TMAO, 3‐MH, and carnosine (Figure S5B through S5G). A strong positive correlation was also found between urea and 3‐MH (Figure S5H), and between 2‐oxoarginine and 4‐guanidinobutanoate (Figure S5I).

The majority of significant acylcarnitines (18/22) and carnitine were correlated with nLAD (FDR<0.05, Table S4). Carnitine and acylcarnitines were correlated with one another.

### Metabolic Pathways

**Figure 7:**
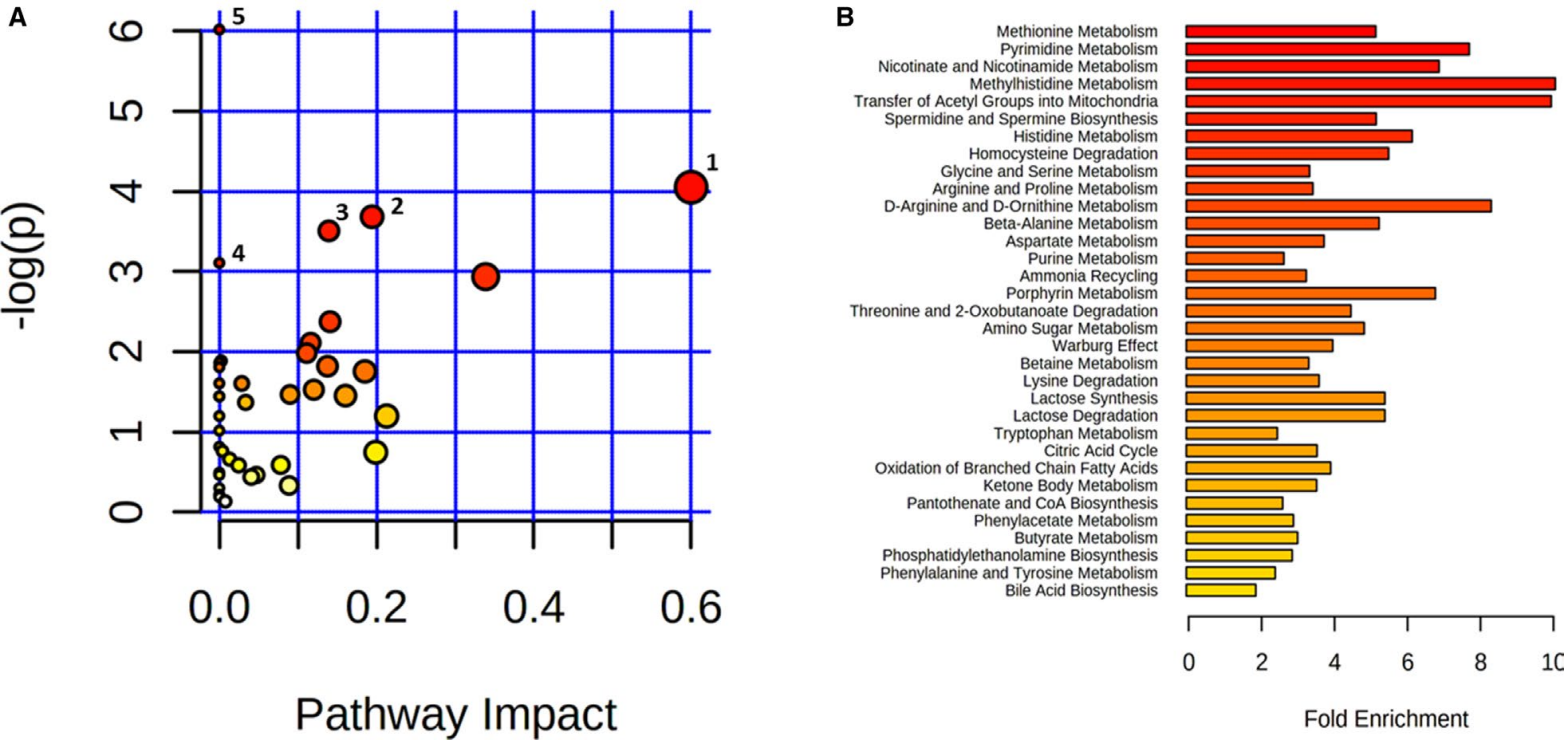
Metabolomic pathway analysis (A) and quantitative metabolite set enrichment analysis (B). **A**, The *y* axis represents the negative of natural logarithm of the *P* values. The significance of each pathway is represented by moving upwards on the *y* axis and by the color scale from white (low significance) to red (high significance). The impact of each pathway is represented by moving rightward on the *x* axis and by the diameter of each circle. The 5 pathways with *P*<0.05 are: (1) synthesis and degradation of ketone bodies; (2) nicotinate and nicotinamide metabolism; (3) histidine metabolism; (4) valine, leucine, and isoleucine biosynthesis; and (5) arginine biosynthesis. **B**, The significance of each enriched metabolite set is represented by the color scale from white (low significance) to red (high significance) as shown in the key. Fold of enrichment is indicated in the *x* axis.

Five KEGG pathways, including arginine biosynthesis, synthesis, and degradation of ketone bodies, nicotinate and nicotinamide metabolism, histidine metabolism, and branched chain amino acid biosynthesis, were overrepresented (*P*<0.05, Figure 7A and Table S5). In addition, qMSEA identified 33 metabolic pathways enriched in group C/D over group A (Figure 7B and Table S6). The overrepresented KEGG pathways except branched chain amino acid biosynthesis pathway were captured by both MetPA and qMSEA. Methionine metabolism, betaine metabolism, and homocysteine degradation pathways were associated with sulfur amino acid metabolism and methylation. Methylhistidine and histidine pathways were overrepresented in dogs with CHF. Many of the enriched pathways, including transfer of acetyl groups into mitochondria, citric acid cycle, lactose synthesis and degradation, ketone body metabolism, oxidation of branched chain FAs, butyrate metabolism, pantothenate (vitamin B5) and coenzyme A biosynthesis, nicotinate and nicotinamide metabolism, lysine degradation, threonine, and 2‐oxobutyric acid degradation were associated with or led to energy productions. Tryptophan metabolism and bile acid biosynthesis pathways were also overrepresented in group C/D.

## Discussion

Our analysis demonstrated robust changes in the metabolome of dogs with different stages of MMVD that affected several key metabolic pathways. Although the majority of these changes occurred at the stage of CHF, 41% of the significant metabolites had changes at the very early B1 stage, providing an opportunity to understand this early transition during the disease progression. In addition, a small number of metabolites had changes that occurred in the absence of overt congestion or any evidence of volume overload. Could they be early compensatory responses? Derangements in cardiac bioenergetics contribute to the pathogenesis of CHF in humans and animal models.^15^, ^34^, ^35^ Previous transcriptomics analysis of mitral valve and left ventricular tissues, as well as metabolomics analysis of serum samples, have suggested compromised long‐chain FA oxidation and increased reliance on glycolysis in dogs with preclinical MMVD compared with healthy dogs.^21^, ^22^ Recent studies demonstrated increased ketone body utilization in well‐defined mouse models of HF and in the failing human heart.^11^, ^12^ A higher ketone body uptake was reported by canine failing hearts in vivo.^36^ Therapeutic ketosis holds significant promise in HF.^37^ Supplement of ketogenic medium‐chain triglycerides in diets ameliorated cardiac remodeling in spontaneously hypertensive rats and reduced left atrial enlargements in dogs with early‐stage MMVD.^38^, ^39^ In this study, the concentrations of both BHBA and acetoacetate, which account for >90% of ketone bodies in man, were increased in B1, B2, and C/D MMVD dogs compared with healthy dogs. Our result was consistent with the observation that blood ketone bodies were elevated in human patients with CHF as compared with those free of CHF, and were in proportion to the severity of cardiac dysfunction.^40^, ^41^

Catabolism of threonine, serine, and methionine converges to α‐ketobutyrate, which enters mitochondrial matrix to produce succinyl‐coenzyme A via the propionate catabolic pathway. Accumulations of α‐ketobutyrate, TCA cycle intermediates, and inorganic phosphate signified perturbations in oxidative phosphorylation. In sarcolemma, creatine, which is produced in the liver and kidneys, is taken up by a membrane creatine transporter from the bloodstream.^42^ Creatine kinase catalyzes the transfer of high‐energy phosphate bond in ATP to creatine to form phosphocreatine, a small molecule that rapidly diffuses from mitochondria to myofibrils.^9^ Increased level of circulating creatine may suggest impaired creatine transporter or uncoupling of energy production and transfer. Acylcarnitines are key intermediates of long‐chain FA transport and oxidation. Circulating acylcarnitines accumulate as a result of incomplete or inefficient FA oxidation and have been used as diagnostic markers for disorders in peroxisomal or mitochondrial oxidation processes.^43^, ^44^, ^45^ Elevated plasma long‐chain acylcarnitines were documented in human patients with HF compared with normal controls.^46^, ^47^ Accumulation of long‐chain acylcarnitines may contribute to the HF by stimulating reactive oxygen species production and releasing circulating inflammatory mediators.^47^ Twenty‐two acylcarnitines were associated with increased MMVD severity in dogs. Some of these acylcarnitines were decreased in response to a diet intervention with demonstrated clinical benefits in MMVD dogs.^22^ Taken together, our data suggest perturbed energy metabolic machinery in dogs with MMVD.

Carnitine level was also significantly increased in dogs with CHF. Carnitine plays a key role in the transport of long‐chain FAs into mitochondrial matrix for oxidation, and its deficiency was associated with dilated cardiomyopathy in dogs and humans.^48^, ^49^ However, elevations in circulating carnitine concentration have also been reported in human patients with dilated cardiomyopathy and CHF.^50^, ^51^ It is possible that the increase of carnitine concentration in circulation was caused by reduced capacity of FA oxidation in dogs with CHF or signifies a compensatory effort for the failing heart to increase FA oxidation. It is also possible that the elevated level of circulating carnitine contributed to the TMAO production.

In mammalian cells, NAD^+^ is an essential cofactor for mitochondrial bioenergetics and important for TCA cycle, glycolysis, and FA oxidation. It is synthesized by 3 pathways, the main salvage pathway that recycles nicotinamide or nicotinamide riboside, the de novo biosynthesis through the kynurenine pathway from L‐tryptophan, and the Preiss‐Handler pathway from nicotinic acid.^52^ The concentration of nicotinamide was reduced in groups B1, B2, and C/D, compared with group A. In contrast, quinolinate, a key intermediate of the kynurenine pathway, was increased in dogs with CHF compared with dogs in other groups. Higher circulating level of quinolinate was reported in human patients with CHF versus healthy patients and was associated with a higher mortality rate.^53^ Thus, it is possible that the predominant salvage pathway is compromised in MMVD dogs, and the de novo NAD^+^ biosynthesis is activated.

The “gut hypothesis” in cardiovascular disease and HF has gained considerable attention in recent years.^16^, ^17^ Evidence for the causal association has begun to emerge.^54^ Several notable gut microbiota‐mediated metabolic pathways and metabolites, including short‐chain FAs, bile acids, and TMA/TMAO, were associated with cardiovascular disease and HF.^16^ Gut microbiota metabolize dietary nutrients such as L‐carnitine, phosphatidylcholine, choline, and betaine to produce trimethylamine, which is oxidized to TMAO in the liver and released into circulation (Figure 5G). Although less efficient than trimethylamine, trimethyllysine is another source of TMAO, and can also serve as the substrate for the synthesis of γ‐butyrobetaine and TMAVA.^55^ Circulating TMAO levels were higher in patients with HF versus healthy humans and dogs.^20^, ^56^ Both B2 preclinical dogs and CHF dogs had higher TMAO levels than healthy dogs, suggesting that the change began before the onset of CHF. However, the causal relationship between the TMAO‐mediated microbial pathway and canine MMVD has yet to be established. Integrational analysis with microbiome changes from these dogs may provide additional insights.

Several uremic toxins were identified in this study. Deficiency in arginase, the enzyme that catalyzes the conversion from arginine to urea in the urea cycle, results in accumulations of guanidino compounds. The levels of 3 guanidino compounds, argininate, 2‐oxoarginine, and 4‐guanidinobutanoate, were elevated in groups B1, B2, and C/D versus group A. TMAP, a novel marker for kidney function, was also increased in CHF dogs versus non‐CHF dogs. The changes in blood uremic toxins, including TMAO, guanidino compounds, and urea and uric acid, as well as TMAP, provide yet another example of the complex interplay along the cardiorenal axis.^57^ It will be interesting to sort out which of these uremic toxins are relevant to mitral regurgitation or MMVD, and those that are associated with CHF. Nonetheless, pharmaceutical or nutritional therapies to reduce these circulating uremic toxins and to improve renal functions may provide clinical benefits to patients with MMVD or CHF.

A majority of the significant metabolites were correlated with both nLAD and ratio of the left atrial diameter to the aortic root diameter, but not left ventricular internal dimensions in end‐diastole. Left atrial enlargement was considered to be the most reliable independent indicator for increased risk of progression of MMVD in dogs.^58^ This may provide opportunities to explore potential markers for progression or risk prediction. The significance of this, if any, remains to be determined.

The MetPA approach depends on a list of significant metabolites that meet the stringent threshold to discern telltale biological clues, while the qMSEA approach uses predefined pathways based on prior biological knowledge to determine whether and how many members of a metabolic pathway act in concert to exert cellular functions. Degradations of homocysteine, methionine, or threonine lead to the production of α‐ketobutyrate, and lysine catabolism generates acetyl‐coenzyme A in the mitochondria. In our study, >30% of metabolic pathways identified by qMSEA were associated with or led to energy productions. Perturbations in arginine and proline metabolism, ammonia recycling, and urea cycle were evidenced with changes in several uremic toxins in the blood. The 3‐MH is the methylation product of histidine residues on the main myofibrillar proteins actin and myosin. Muscle atrophy or cachexia is common in dogs with CHF.^59^, ^60^ Myofibrillar protein overdegradation was reported in human patients with clinically stable CHF, resulting in a higher circulating level of 3‐MH when compared with healthy patients.^61^ The level of 1‐methylhistidine, which derives from dietary anserine (β‐alanyl‐3‐methyl‐L‐histidine), was inversely associated with left ventricular diastolic function in humans.^62^, ^63^ Finally, tryptophan serves as a substrate for the generation of several important compounds, such as conversion to serotonin and degradation through kynurenine pathway leading to the de novo NAD^+^ biosynthesis.^64^ In summary, our data revealed amino acid metabolic reprogramming and renal insufficiency in the pathogenesis of canine MMVD. Changes in numerous circulating metabolites raised the opportunity for new therapeutic targets and biomarkers that could be used for diagnosis, prognosis, or interventional studies.

Our study underscored the challenge of matching age and body weight between healthy dogs and dogs with MMVD. MMVD is prevalent in small‐breed geriatric dogs, and dogs with CHF generally experience body weight loss attributable to cachexia.^60^ Several attempts were made to reduce age and body weight differences without success. The bootstrap resampling experiment supported the hypothesis that the observed difference was independent of age or body weight. Despite this effort, we cannot completely rule out the possibility of small confounding effects from age or body weight. Our analysis also showed no difference between dogs in stable cardiac medications and those that were not. This study is one of our initial efforts towards the understanding of canine MMVD progression and pathogenesis. A targeted metabolomics study on a different cohort of dogs can be used to confirm the findings. A multilayered approach that integrates metabolomics and other “omics” data will allow us to delve further into the mechanisms^8^ Last, many pet dogs are often given treats and human food in addition to their base dog food diets, making it difficult to accurately assess diet effects. In addition, we were unable to rule out potential confounding effects from breed or fasting state in this study. A study with research colony dogs may address these limitations. However, the challenge would be to enroll a large number of research dogs with naturally occurring MMVD of different stages.

## Sources of Funding

The study was funded by the Nestlé Purina PetCare Company.

## Disclosures

Li is a current employee of the Nestlé Purina PetCare Company. The remaining authors have no disclosures to report.

**Table 1:** Physical Characteristics, Echocardiography, and Common Cardiac Medications of the Dogs

ACVIM Stage	A	B1	B2	C/D	*P* Value
Sample size	27	22	18	17	
Male/female	14/13	12/10	11/7	10/7	0.93
Age, y	8.3±0.6	10.2±0.5	10.4±0.6	12.4±0.5	<0.0001
Body weight, kg	14.9±1.6	10.5±1.1	8.1±0.9	7.8±1.0	0.0004
BCS (1–9)	5.1±0.2	5.6±0.2	5.2±0.3	4.8±0.3	0.14
Cardiac medications					
Pimobendan	0	4	15	15	
Lasix	0	1	2	15	
ACEI	1	0	5	10	
Spironolactone	0	0	1	8	
Echocardiography*					
nLVIDd, cm	1.47±0.04	1.47±0.04	1.80±0.05	2.03±0.09	<0.0001
nLVIDs, cm	0.98±0.04	0.88±0.04	0.89±0.05	1.00±0.07	0.18
nLAD, cm	0.97±0.03	1.04±0.03	1.30±0.06	1.51±0.08	<0.0001
nAoD, cm	0.76±0.02	0.76±0.02	0.80±0.04	0.72±0.03	0.23
LA/Ao	1.30±0.05	1.40±0.05	1.70±0.10	2.17±0.12	<0.0001

Continuous variables are reported as mean±standard error. ACEI indicates angiotensin‐converting enzyme inhibitor; ACVIM, American College of Veterinary Internal Medicine; BCS, body condition score; LA/Ao, left atrial to aortic root diameter ratio; nAoD, normalized aortic root diameter; nLAD, normalized left atrial diameter; nLVIDd, normalized left ventricular internal diameter end‐diastole; and nLVIDs, normalized left ventricular internal diameter end‐systole.

* Eight group A dogs had no echocardiography.

## Supporting information


Supplementary Methods
Tables S1–S6
Figures S1–S5

DataS1

DataS2
