# Supplementary material for: Metabolomics Analysis Reveals Deranged Energy Metabolism and Amino Acid Metabolic Reprogramming in Dogs With Myxomatous Mitral Valve Disease
Source: J Am Heart Assoc. 2021 Apr 23;10(9):e018923. doi: 10.1161/JAHA.120.018923 (PMC8200728; doi:10.1161/JAHA.120.018923)

# SUPPLEMENTAL MATERIAL

## Supplemental Methods

### *Metabolon Platform*

**Sample Accessioning:** Following receipt, samples were inventoried and immediately stored at -80°C. Each sample received was accessioned into the Metabolon LIMS system and was assigned by the LIMS a unique identifier that was associated with the original source identifier only. This identifier was used to track all sample handling, tasks, results, etc. The samples (and all derived aliquots) were tracked by the LIMS system. All portions of any sample were automatically assigned their own unique identifiers by the LIMS when a new task was created; the relationship of these samples was also tracked. All samples were maintained at -80°C until processed.

**Sample Preparation:** Samples were prepared using the automated MicroLab STAR® system from Hamilton Company. Several recovery standards were added prior to the first step in the extraction process for QC purposes. To remove protein, dissociate small molecules bound to protein or trapped in the precipitated protein matrix, and to recover chemically diverse metabolites, proteins were precipitated with methanol under vigorous shaking for 2 min (Glen Mills GenoGrinder 2000) followed by centrifugation. The resulting extract was divided into five fractions: two for analysis by two separate reverse phase (RP)/UPLC-MS/MS methods with positive ion mode electrospray ionization (ESI), one for analysis by RP/UPLC-MS/MS with negative ion mode ESI, one for analysis by HILIC/UPLC-MS/MS with negative ion mode ESI, and one sample was reserved for backup. Samples were placed briefly on a TurboVap® (Zymark) to remove the organic solvent. The sample extracts were stored overnight under nitrogen before preparation for analysis.

**QA/QC:** Several types of controls were analyzed in concert with the experimental samples: a pooled matrix sample generated by taking a small volume of each experimental sample (or alternatively, use of a pool of well-characterized human plasma) served as a technical replicate throughout the data set; extracted water samples served as process blanks; and a cocktail of QC standards that were carefully chosen not to interfere with the measurement of endogenous compounds were spiked into every analyzed sample, allowed instrument performance monitoring and aided chromatographic alignment. Tables 1 and 2 describe these QC samples and standards. Instrument variability was determined by calculating the median relative standard deviation (RSD) for the standards that were added to each sample prior to injection into the mass spectrometers. Overall process variability was determined by calculating the median RSD for all endogenous metabolites (i.e., non-instrument standards) present in 100% of the pooled matrix samples. Experimental samples were randomized across the platform run with QC samples spaced evenly among the injections, as outlined in Figure 1.

**Table 1: Description of Metabolon QC Samples**

| Type | Description | Purpose |
|---|---|---|
| MTRX | Large pool of human plasma maintained by Metabolon that has been characterized extensively. | Assure that all aspects of the Metabolon process are operating within specifications. |
| CMTRX | Pool created by taking a small aliquot from every customer sample. | Assess the effect of a non-plasma matrix on the Metabolon process and distinguish biological variability from process variability. |
| PRCS | Aliquot of ultra-pure water | Process Blank used to assess the contribution to compound signals from the process. |
| SOLV | Aliquot of solvents used in extraction. | Solvent Blank used to segregate contamination sources in the extraction. |

**Table 2: Metabolon QC Standards**

| Type | Description | Purpose |
|---|---|---|
| RS | Recovery Standard | Assess variability and verify performance of extraction and instrumentation. |
| IS | Internal Standard | Assess variability and performance of instrument. |

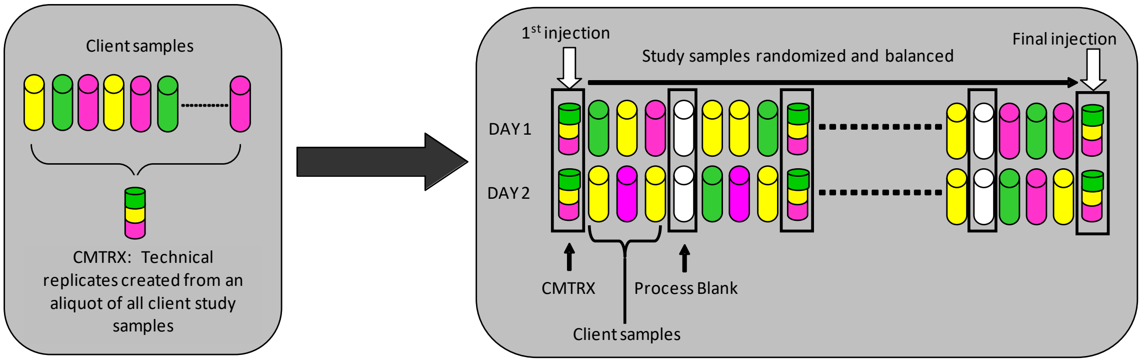


**Figure 1. Preparation of client-specific technical replicates.** A small aliquot of each client sample (colored cylinders) is pooled to create a CMTRX technical replicate sample (multi-colored cylinder), which is then injected periodically throughout the platform run. Variability among consistently detected biochemicals can be used to calculate an estimate of overall process and platform variability.

**Ultrahigh Performance Liquid Chromatography-Tandem Mass Spectroscopy (UPLC-MS/MS):** All methods utilized a Waters ACQUITY ultra-performance liquid chromatography (UPLC) and a Thermo Scientific Q-Exactive high resolution/accurate mass spectrometer interfaced with a heated electrospray ionization (HESI-II) source and Orbitrap mass analyzer operated at 35,000 mass resolution. The sample extract was dried then reconstituted in solvents compatible to each of the four methods. Each reconstitution solvent contained a series of standards at fixed concentrations to ensure injection and chromatographic consistency. One aliquot was analyzed using acidic positive ion conditions, chromatographically optimized for more hydrophilic compounds. In this method, the extract was gradient eluted from a C18 column (Waters UPLC BEH C18-2.1x100 mm, 1.7 µm) using water and methanol, containing 0.05% perfluoropentanoic acid (PFPA) and 0.1% formic acid (FA). Another aliquot was also analyzed using acidic positive ion conditions, however it was chromatographically optimized for more hydrophobic compounds. In this method, the extract was gradient eluted from the same afore mentioned C18 column using methanol, acetonitrile, water, 0.05% PFPA and 0.01% FA and was operated at an overall higher organic content. Another aliquot was analyzed using basic negative ion optimized conditions using a separate dedicated C18 column. The basic extracts were gradient eluted from the column using methanol and water, however with 6.5mM Ammonium Bicarbonate at pH 8. The fourth aliquot was analyzed via negative ionization following elution from a HILIC column (Waters UPLC BEH Amide 2.1x150 mm, 1.7 µm) using a gradient consisting of water and acetonitrile with 10mM Ammonium Formate, pH 10.8. The MS analysis alternated between MS and data-dependent $MS^n$ scans using dynamic exclusion. The scan range varied slighted between methods but covered 70-1000 m/z. Raw data files are archived and extracted as described below.

**Bioinformatics:** The informatics system consisted of four major components, the Laboratory Information Management System (LIMS), the data extraction and peak-identification software, data processing tools for QC and compound identification, and a collection of information interpretation and visualization tools for use by data analysts. The hardware and software foundations for these informatics components were the LAN backbone, and a database server running Oracle 10.2.0.1 Enterprise Edition.

**LIMS:** The purpose of the Metabolon LIMS system was to enable fully auditable laboratory automation through a secure, easy to use, and highly specialized system. The scope of the Metabolon LIMS system encompasses sample accessioning, sample preparation and instrumental analysis and reporting and advanced data analysis. All of the subsequent software systems are grounded in the LIMS data structures. It has been modified to leverage and interface with the in-house information extraction and data visualization systems, as well as third party instrumentation and data analysis software.

**Data Extraction and Compound Identification:** Raw data was extracted, peak-identified and QC processed using Metabolon's hardware and software. These systems are built on a web-service platform utilizing Microsoft's .NET technologies, which run on high-performance application servers and fiber-channel storage arrays in clusters to provide active failover and load-balancing. Compounds were identified by comparison to library entries of purified standards or recurrent unknown entities. Metabolon maintains a library based on authenticated standards that contains the retention time/index (RI), mass to charge ratio (*m/z)*, and chromatographic data (including MS/MS spectral data) on all molecules present in the library. Furthermore, biochemical identifications are based on three criteria: retention index within a narrow RI window of the proposed identification, accurate mass match to the library +/- 10 ppm, and the

MS/MS forward and reverse scores between the experimental data and authentic standards. The MS/MS scores are based on a comparison of the ions present in the experimental spectrum to the ions present in the library spectrum. While there may be similarities between these molecules based on one of these factors, the use of all three data points can be utilized to distinguish and differentiate biochemicals. More than 3300 commercially available purified standard compounds have been acquired and registered into LIMS for analysis on all platforms for determination of their analytical characteristics. Additional mass spectral entries have been created for structurally unnamed biochemicals, which have been identified by virtue of their recurrent nature (both chromatographic and mass spectral). These compounds have the potential to be identified by future acquisition of a matching purified standard or by classical structural analysis.

**Curation:** A variety of curation procedures were carried out to ensure that a high quality data set was made available for statistical analysis and data interpretation. The QC and curation processes were designed to ensure accurate and consistent identification of true chemical entities, and to remove those representing system artifacts, mis-assignments, and background noise. Metabolon data analysts use proprietary visualization and interpretation software to confirm the consistency of peak identification among the various samples. Library matches for each compound were checked for each sample and corrected if necessary.

**Metabolite Quantification and Data Normalization:** Peaks were quantified using area-under-the-curve. For studies spanning multiple days, a data normalization step was performed to correct variation resulting from instrument inter-day tuning differences. Essentially, each compound was corrected in run-day blocks by registering the medians to equal one (1.00) and normalizing each data point proportionately (termed the "block correction"; Figure 2). For studies that did not require more than one day of analysis, no normalization is necessary, other than for purposes of data visualization. In certain instances, biochemical data may have been normalized to an additional factor (e.g., cell counts, total protein as determined by Bradford assay, osmolality, etc.) to account for differences in metabolite levels due to differences in the amount of material present in each sample.

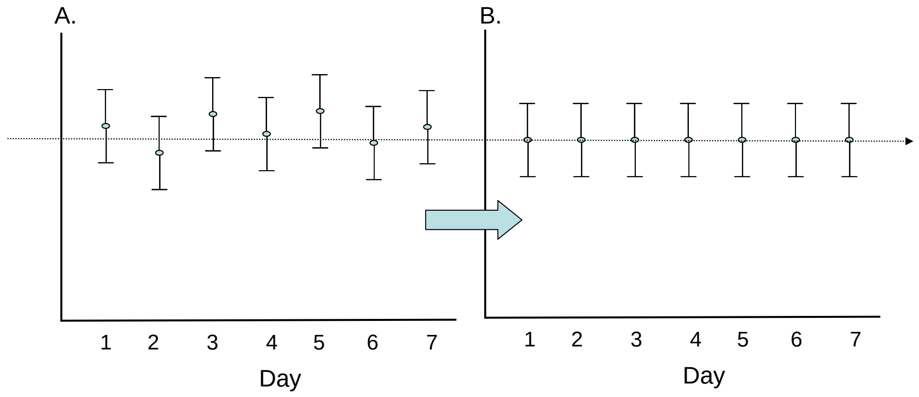


**Figure 2: Visualization of data normalization steps for a multiday platform run.**

**Table S1.** Metabolites that were significantly different among the four ACVIM groups.

| | Linear model | | Adjusted P values from pairwise comparisons | | | | | | Mean, log2 transformed | | | | Fold changes | | | | | | Pathways | | | | | |
|---|---|---|---|---|---|---|---|---|---|---|---|---|---|---|---|---|---|---|---|---|---|---|---|---|
| **BIOCHEMICAL** | **P value** | **FDR** | **A v B1** | **A v B2** | **A v C/D** | **B1 v B2** | **B1 v C/D** | **B2 v C/D** | **A** | **B1** | **B2** | **C/D** | **B1/A** | **B2/A** | **CD/A** | **CD/B1** | **CD/B2** | **B2/B1** | **SUPER_PATH** | **SUB_PATHWAY** | **PUBCHEM** | **CAS** | **KEGG** | **HMDB** |
| hydroxyasparagine | 0 | 0 | 0.9158 | 0.5446 | 0 | 0.5446 | 0 | 0 | -0.356 | -0.33 | -0.132 | 1.132 | 1.018 | 1.168 | 2.804 | 2.756 | 2.401 | 1.148 | Amino Acid | Alanine and Aspartate Metabolism | 97663 | | C03124 | HMDB32332 |
| N-acetylalanine | 0 | 0.0001 | 0.0816 | 0.1206 | 0 | 0.8626 | 0.001 | 0.001 | -0.523 | -0.039 | -0.087 | 0.973 | 1.398 | 1.353 | 2.819 | 2.017 | 2.084 | 0.968 | Amino Acid | Alanine and Aspartate Metabolism | 88064 | 97-69-8 | C02847 | HMDB00766 |
| N-acetylasparagine | 0 | 0.0001 | 0.0031 | 0.0015 | 0 | 0.6188 | 0.0891 | 0.2146 | -0.696 | 0.126 | 0.264 | 0.664 | 1.768 | 1.946 | 2.567 | 1.452 | 1.32 | 1.101 | Amino Acid | Alanine and Aspartate Metabolism | 99715 | 4033-40-3 | | HMDB06028 |
| N-carbamoylalanine | 0.0052 | 0.024 | 0.0992 | 0.3867 | 0.0236 | 0.0236 | 0.4185 | 0.01 | -0.222 | 0.28 | -0.507 | 0.527 | 1.417 | 0.821 | 1.681 | 1.187 | 2.048 | 0.579 | Amino Acid | Alanine and Aspartate Metabolism | 426409 | 77340-50-2 | | |
| N,N-dimethylalanine | 0.0082 | 0.0349 | 0.1143 | 0.7388 | 0.1099 | 0.2355 | 0.0051 | 0.1099 | 0.033 | -0.457 | -0.064 | 0.606 | 0.712 | 0.935 | 1.487 | 2.088 | 1.59 | 1.313 | Amino Acid | Alanine and Aspartate Metabolism | 5488191 | 2812-31-9 | | |
| creatine | 0 | 0 | 0.0151 | 0 | 0 | 0.0073 | 0.0027 | 0.6561 | -0.738 | -0.145 | 0.601 | 0.723 | 1.509 | 2.53 | 2.754 | 1.825 | 1.089 | 1.677 | Amino Acid | Creatine Metabolism | 586 | 57-00-1 | C00300 | HMDB00064 |
| alpha-ketoglutaramate* | 0.0001 | 0.0009 | 0.1807 | 0.585 | 0.0001 | 0.4491 | 0.0047 | 0.0007 | -0.402 | 4E-04 | -0.253 | 0.905 | 1.321 | 1.109 | 2.473 | 1.872 | 2.231 | 0.839 | Amino Acid | Glutamate Metabolism | 48 | 18465-19-5 | | |
| carboxyethyl-GABA | 0.0009 | 0.0055 | 0.3716 | 0.5805 | 0.008 | 0.7059 | 0.0013 | 0.0037 | -0.048 | -0.357 | -0.246 | 0.799 | 0.807 | 0.872 | 1.799 | 2.229 | 2.064 | 1.08 | Amino Acid | Glutamate Metabolism | 2572 | 3/2/4386 | | HMDB02201 |
| glutamine | 0.0032 | 0.0157 | 0.2137 | 0.1762 | 0.0024 | 0.7746 | 0.0659 | 0.1068 | 0.411 | 0.044 | -0.042 | -0.666 | 0.775 | 0.73 | 0.474 | 0.611 | 0.649 | 0.942 | Amino Acid | Glutamate Metabolism | 5961 | 56-85-9 | C00064 | HMDB00641 |
| N-acetylglutamate | 0.0102 | 0.0418 | 0.3284 | 0.2489 | 0.0059 | 0.7329 | 0.0718 | 0.1285 | -0.369 | -0.068 | 0.035 | 0.638 | 1.232 | 1.324 | 2.01 | 1.632 | 1.519 | 1.074 | Amino Acid | Glutamate Metabolism | 70914 | 8/3/5817 | C00624 | HMDB01138 |
| N-acetylglutamine | 0 | 0.0006 | 0.0484 | 0.0383 | 0 | 0.6898 | 0.0205 | 0.0383 | -0.551 | -0.015 | 0.099 | 0.788 | 1.45 | 1.569 | 2.529 | 1.745 | 1.612 | 1.082 | Amino Acid | Glutamate Metabolism | 182230 | 2490-97-3 | C02716 | HMDB06029 |
| 2-hydroxybutyrate/2-hydroxyisobut | 0 | 0 | 0.0004 | 0 | 0 | 0.4142 | 0.1358 | 0.4594 | -0.787 | 0.155 | 0.408 | 0.619 | 1.921 | 2.289 | 2.65 | 1.379 | 1.157 | 1.192 | Amino Acid | Glutathione Metabolism | | | | |
| glycine | 0.0005 | 0.0035 | 0.1499 | 0.0038 | 0.0081 | 0.0976 | 0.1499 | 0.7184 | 0.504 | 0.09 | -0.5 | -0.387 | 0.751 | 0.499 | 0.54 | 0.719 | 1.082 | 0.664 | Amino Acid | Glycine, Serine and Threonine Metabolism | 750 | 56-40-6 | C00037 | HMDB00123 |
| N-acetylserine | 0.0004 | 0.003 | 0.1216 | 0.0716 | 0.0002 | 0.6703 | 0.0215 | 0.0565 | -0.486 | -0.052 | 0.072 | 0.762 | 1.351 | 1.473 | 2.375 | 1.757 | 1.613 | 1.09 | Amino Acid | Glycine, Serine and Threonine Metabolism | 65249 | 97-14-3 | | HMDB02931 |
| N-acetylthreonine | 0 | 0 | 0.0037 | 0.0019 | 0 | 0.6182 | 0.0103 | 0.0385 | -0.711 | 0.069 | 0.203 | 0.825 | 1.717 | 1.884 | 2.9 | 1.689 | 1.539 | 1.097 | Amino Acid | Glycine, Serine and Threonine Metabolism | 152204 | 17093-74-2 | | HMDB62557 |
| sarcosine | 0.011 | 0.0437 | 0.0208 | 0.9025 | 0.5837 | 0.0387 | 0.0144 | 0.5837 | -0.164 | 0.591 | -0.128 | -0.369 | 1.688 | 1.025 | 0.867 | 0.514 | 0.846 | 0.607 | Amino Acid | Glycine, Serine and Threonine Metabolism | 1088 | 107-97-1 | C00213 | HMDB00271 |
| 4-guanidinobutanoate | 0.0124 | 0.0478 | 0.0743 | 0.0203 | 0.0203 | 0.5246 | 0.5246 | 0.9753 | -0.495 | 0.084 | 0.334 | 0.324 | 1.494 | 1.777 | 1.764 | 1.181 | 0.993 | 1.189 | Amino Acid | Guanidino and Acetamido Metabolism | 500 | 463-003;463-( | C01035 | HMDB03464 |
| 1-methyl-5-imidazoleacetate | 0.0003 | 0.0022 | 0.6647 | 0.9478 | 0.0005 | 0.6647 | 0.0024 | 0.001 | -0.291 | -0.101 | -0.273 | 0.882 | 1.141 | 1.013 | 2.255 | 1.978 | 2.227 | 0.888 | Amino Acid | Histidine Metabolism | 6451814 | 4200-48-0 | | HMDB04988 |
| 1-methylhistidine | 0 | 0 | 0.3465 | 0.2562 | 0 | 0.7211 | 0 | 0 | -0.483 | -0.244 | -0.155 | 1.246 | 1.18 | 1.255 | 3.313 | 2.807 | 2.64 | 1.063 | Amino Acid | Histidine Metabolism | 92105 | 332-80-9 | C01152 | HMDB00001 |
| 3-methylhistidine | 0 | 0.0002 | 0.2057 | 0.2495 | 0 | 0.8879 | 0.0008 | 0.0008 | -0.459 | -0.083 | -0.122 | 0.966 | 1.298 | 1.264 | 2.686 | 2.069 | 2.126 | 0.973 | Amino Acid | Histidine Metabolism | 64969 | 368-16-1 | C01152 | HMDB00479 |
| carnosine | 0.0007 | 0.0045 | 0.6641 | 0.2386 | 0.0007 | 0.4037 | 0.0022 | 0.0332 | -0.349 | -0.234 | 0.048 | 0.807 | 1.083 | 1.317 | 2.229 | 2.059 | 1.693 | 1.216 | Amino Acid | Histidine Metabolism | 439224 | 305-84-0 | C00386 | HMDB00033 |
| formiminoglutamate | 0 | 0.0001 | 0.0259 | 0.0149 | 0 | 0.6106 | 0.0103 | 0.0259 | -0.602 | -0.014 | 0.128 | 0.837 | 1.503 | 1.658 | 2.711 | 1.804 | 1.635 | 1.103 | Amino Acid | Histidine Metabolism | 439233 | 816-90-0 | C00439 | HMDB00854 |
| imidazole lactate | 0.0002 | 0.0018 | 0.0077 | 0.0077 | 0.0002 | 0.8901 | 0.21 | 0.2397 | -0.625 | 0.154 | 0.194 | 0.589 | 1.716 | 1.764 | 2.32 | 1.352 | 1.315 | 1.028 | Amino Acid | Histidine Metabolism | 793 | 14403-45-3 | C05568 | HMDB02320 |
| N-acetyl-1-methylhistidine* | 0 | 0 | 0.0342 | 0.0727 | 0 | 0.7835 | 0 | 0 | -0.588 | -0.053 | -0.123 | 1.134 | 1.449 | 1.38 | 3.298 | 2.276 | 2.39 | 0.952 | Amino Acid | Histidine Metabolism | 53859791 | | | |
| N-acetyl-3-methylhistidine* | 0 | 0.0004 | 0.0892 | 0.0892 | 0 | 0.9362 | 0.0088 | 0.011 | -0.502 | -0.033 | -0.011 | 0.852 | 1.384 | 1.406 | 2.556 | 1.847 | 1.818 | 1.016 | Amino Acid | Histidine Metabolism | 193270 | 37841-04-6 | | |
| 2-hydroxy-3-methylvalerate | 0.0003 | 0.0025 | 0.0042 | 0.0063 | 0.004 | 0.9372 | 0.7875 | 0.7875 | -0.616 | 0.259 | 0.236 | 0.392 | 1.834 | 1.805 | 2.011 | 1.096 | 1.114 | 0.984 | Amino Acid | Leucine, Isoleucine and Valine Metabolism | 164623 | 488-15-3 | | HMDB00317 |
| 3-hydroxy-2-ethylpropionate | 0 | 0 | 0.021 | 0.0028 | 0 | 0.3129 | 0.0117 | 0.1009 | -0.646 | -0.019 | 0.261 | 0.775 | 1.544 | 1.875 | 2.677 | 1.734 | 1.428 | 1.214 | Amino Acid | Leucine, Isoleucine and Valine Metabolism | 188979 | 4374-62-3 | | HMDB00396 |
| 3-hydroxyisobutyrate | 0.002 | 0.0107 | 0.0784 | 0.0784 | 0.0014 | 0.8728 | 0.1042 | 0.1347 | -0.503 | 0.058 | 0.106 | 0.612 | 1.476 | 1.525 | 2.166 | 1.468 | 1.42 | 1.034 | Amino Acid | Leucine, Isoleucine and Valine Metabolism | 87 | 2068-83-9 | C06001 | HMDB00336 |
| 3-methyl-2-oxobutyrate | 0.0004 | 0.0029 | 0.051 | 0.0061 | 0.0024 | 0.3864 | 0.2012 | 0.6124 | -0.564 | 0.04 | 0.333 | 0.492 | 1.52 | 1.862 | 2.079 | 1.368 | 1.117 | 1.225 | Amino Acid | Leucine, Isoleucine and Valine Metabolism | 49 | 3715-29-5 | C00141 | HMDB00019 |
| 3-methylglutaconate | 0 | 0.0005 | 0.0223 | 0.2197 | 0 | 0.2916 | 0.0239 | 0.0037 | -0.527 | 0.134 | -0.165 | 0.838 | 1.581 | 1.285 | 2.575 | 1.629 | 2.004 | 0.813 | Amino Acid | Leucine, Isoleucine and Valine Metabolism | 1551553 | 5746-90-7 | | HMDB00522 |
| ethylmalonate | 0 | 0 | 0.1001 | 0.1041 | 0 | 0.9844 | 0.0005 | 0.0006 | -0.527 | -0.073 | -0.079 | 1.015 | 1.37 | 1.365 | 2.912 | 2.126 | 2.134 | 0.996 | Amino Acid | Leucine, Isoleucine and Valine Metabolism | 11756 | 601-75-2 | | HMDB00622 |
| isobutyrylcarnitine (C4) | 0 | 0 | 0.0194 | 0.0057 | 0 | 0.5295 | 0.0008 | 0.0057 | -0.643 | -0.055 | 0.112 | 0.973 | 1.503 | 1.687 | 3.065 | 2.039 | 1.816 | 1.123 | Amino Acid | Leucine, Isoleucine and Valine Metabolism | 168379 | 25518-49-4 | | HMDB00736 |
| isovalerylcarnitine (C5) | 0.0002 | 0.0017 | 0.0417 | 0.1712 | 0.0001 | 0.545 | 0.0417 | 0.0191 | -0.497 | 0.087 | -0.089 | 0.772 | 1.499 | 1.328 | 2.41 | 1.608 | 1.815 | 0.886 | Amino Acid | Leucine, Isoleucine and Valine Metabolism | 6426851 | 31023-24-2 | | HMDB00688 |
| N-acetylisoleucine | 0 | 0 | 0.0022 | 0.0022 | 0 | 0.875 | 0.0395 | 0.057 | -0.706 | 0.134 | 0.177 | 0.76 | 1.79 | 1.844 | 2.763 | 1.544 | 1.499 | 1.03 | Amino Acid | Leucine, Isoleucine and Valine Metabolism | 306109 | 3077-46-1 | | HMDB61684 |
| N-acetylleucine | 0.0002 | 0.0017 | 0.0235 | 0.066 | 0.0001 | 0.557 | 0.066 | 0.0362 | -0.559 | 0.149 | -0.021 | 0.717 | 1.634 | 1.453 | 2.422 | 1.483 | 1.667 | 0.889 | Amino Acid | Leucine, Isoleucine and Valine Metabolism | 70912 | 1188-21-2 | C02710 | HMDB11756 |
| N-acetylvaline | 0 | 0 | 0.0084 | 0.003 | 0 | 0.5689 | 0.0026 | 0.0084 | -0.682 | -0.002 | 0.149 | 0.928 | 1.603 | 1.78 | 3.054 | 1.906 | 1.716 | 1.111 | Amino Acid | Leucine, Isoleucine and Valine Metabolism | 66789 | 96-81-1 | | HMDB11757 |
| tiglyl carnitine (C5) | 0.0001 | 0.0012 | 0.171 | 0.0967 | 0 | 0.6493 | 0.0048 | 0.0173 | -0.478 | -0.097 | 0.033 | 0.85 | 1.303 | 1.426 | 2.511 | 1.928 | 1.761 | 1.094 | Amino Acid | Leucine, Isoleucine and Valine Metabolism | 22833596 | 64191-86-2 | | HMDB02366 |
| 2-aminoadipate | 0.0001 | 0.0007 | 0.0049 | 0.001 | 0.001 | 0.5175 | 0.5175 | 0.9756 | -0.649 | 0.165 | 0.392 | 0.402 | 1.757 | 2.057 | 2.071 | 1.179 | 1.007 | 1.171 | Amino Acid | Lysine Metabolism | 469 | 542-32-5;1118 | C00956 | HMDB00510 |
| 5-(galactosylhydroxy)-L-lysine | 0 | 0.0001 | 0.248 | 0.248 | 0 | 0.939 | 0.0009 | 0.0013 | -0.44 | -0.119 | -0.098 | 0.957 | 1.249 | 1.268 | 2.634 | 2.108 | 2.077 | 1.015 | Amino Acid | Lysine Metabolism | 123986 | 32448-36-5 | | |
| 6-oxopiperidine-2-carboxylate | 0.0003 | 0.0025 | 0.0097 | 0.1683 | 0.0003 | 0.1683 | 0.1683 | 0.0166 | -0.534 | 0.255 | -0.151 | 0.678 | 1.729 | 1.304 | 2.318 | 1.341 | 1.777 | 0.754 | Amino Acid | Lysine Metabolism | 3014237 | 34622-39-4 | | HMDB61705 |
| N,N,N-trimethyl-5-aminovalerate | 0.0005 | 0.0033 | 0.3693 | 0.0095 | 0.0008 | 0.079 | 0.0093 | 0.3483 | -0.464 | -0.228 | 0.342 | 0.669 | 1.178 | 1.748 | 2.194 | 1.862 | 1.255 | 1.484 | Amino Acid | Lysine Metabolism | 14274897 | | | |
| N2-acetyl,N6-methyllysine | 0.0011 | 0.0066 | 0.1059 | 0.6158 | 0.0024 | 0.2929 | 0.1059 | 0.0132 | -0.376 | 0.116 | -0.233 | 0.693 | 1.406 | 1.104 | 2.098 | 1.493 | 1.9 | 0.786 | Amino Acid | Lysine Metabolism | | | | |
| N6-acetyllysine | 0.0015 | 0.0083 | 0.0081 | 0.0671 | 0.0026 | 0.4684 | 0.4684 | 0.2402 | -0.56 | 0.265 | 0.05 | 0.495 | 1.771 | 1.527 | 2.077 | 1.173 | 1.361 | 0.862 | Amino Acid | Lysine Metabolism | 92832 | 692-04-6 | C02727 | HMDB00206 |
| N6-carboxyethyllysine | 0.0076 | 0.0333 | 0.2212 | 0.5862 | 0.0046 | 0.5123 | 0.0866 | 0.0246 | -0.344 | 0.052 | -0.188 | 0.678 | 1.316 | 1.115 | 2.032 | 1.543 | 1.822 | 0.847 | Amino Acid | Lysine Metabolism | 22283604 | 3/2/5746 | | |
| N6,N6-dimethyllysine | 0.0015 | 0.0085 | 0.1343 | 0.4349 | 0.0009 | 0.4349 | 0.0477 | 0.013 | -0.4 | 0.057 | -0.174 | 0.746 | 1.373 | 1.17 | 2.213 | 1.612 | 1.892 | 0.852 | Amino Acid | Lysine Metabolism | 193344 | 2259-86-1 | C05545 | HMDB13287 |
| N6,N6,N6-trimethyllysine | 0.0011 | 0.0067 | 0.0651 | 0.0759 | 0.0006 | 0.933 | 0.0651 | 0.0651 | -0.496 | 0.059 | 0.034 | 0.676 | 1.469 | 1.444 | 2.254 | 1.534 | 1.561 | 0.983 | Amino Acid | Lysine Metabolism | 440120 | 23284-33-5 | C03793 | HMDB01325 |
| pipecolate | 0.0021 | 0.0113 | 0.0073 | 0.007 | 0.3414 | 0.696 | 0.125 | 0.0887 | -0.487 | 0.351 | 0.467 | -0.176 | 1.788 | 1.938 | 1.24 | 0.694 | 0.64 | 1.084 | Amino Acid | Lysine Metabolism | 849 | 4043-87-2 | C00408 | HMDB00070 |
| 2,3-dihydroxy-5-methylthio-4-pent | 0.0001 | 0.0009 | 0.3584 | 0.6352 | 0.0001 | 0.6415 | 0.0019 | 0.0011 | -0.379 | -0.075 | -0.207 | 0.918 | 1.235 | 1.127 | 2.458 | 1.99 | 2.182 | 0.912 | Amino Acid | Methionine, Cysteine, SAM and Taurine Metabolism | | | | |
| alpha-ketobutyrate | 0.0016 | 0.0091 | 0.0661 | 0.004 | 0.0064 | 0.2339 | 0.3226 | 0.7781 | -0.551 | 0.028 | 0.45 | 0.362 | 1.493 | 2.001 | 1.882 | 1.26 | 0.94 | 1.34 | Amino Acid | Methionine, Cysteine, SAM and Taurine Metabolism | 58 | 600-18-0 | C00109 | HMDB00005 |
| methionine | 0.003 | 0.0153 | 0.2908 | 0.1146 | 0.0453 | 0.0201 | 0.0094 | 0.6098 | 0.163 | 0.481 | -0.35 | -0.512 | 1.246 | 0.701 | 0.626 | 0.502 | 0.893 | 0.562 | Amino Acid | Methionine, Cysteine, SAM and Taurine Metabolism | 6137 | 63-68-3 | C00073 | HMDB00696 |
| S-carboxyethylcysteine | 0.0002 | 0.0019 | 0.0225 | 0.4849 | 0.0002 | 0.1179 | 0.0838 | 0.0029 | -0.467 | 0.204 | -0.275 | 0.768 | 1.592 | 1.143 | 2.354 | 1.478 | 2.06 | 0.718 | Amino Acid | Methionine, Cysteine, SAM and Taurine Metabolism | | 4033-46-9 | | |
| S-methylmethionine | 0.0071 | 0.0314 | 0.638 | 0.0214 | 0.0741 | 0.0497 | 0.166 | 0.5392 | 0.347 | 0.218 | -0.523 | -0.278 | 0.914 | 0.547 | 0.648 | 0.709 | 1.185 | 0.599 | Amino Acid | Methionine, Cysteine, SAM and Taurine Metabolism | 458 | 12/7/3493 | C05319 | HMDB38670 |
| N-acetylphenylalanine | 0.0001 | 0.0006 | 0.0328 | 0.1943 | 0 | 0.4487 | 0.0199 | 0.0053 | -0.513 | 0.085 | -0.131 | 0.843 | 1.513 | 1.303 | 2.56 | 1.692 | 1.964 | 0.861 | Amino Acid | Phenylalanine Metabolism | 74839 | 2018-61-3 | C03519 | HMDB00512 |
| 5-methylthioadenosine (MTA) | 0.0005 | 0.0035 | 0.1924 | 0.2657 | 0.0004 | 0.835 | 0.0198 | 0.0198 | -0.422 | -0.015 | -0.077 | 0.771 | 1.326 | 1.271 | 2.287 | 1.725 | 1.8 | 0.958 | Amino Acid | Polyamine Metabolism | 439176 | 2457-80-9 | C00170 | HMDB01173 |
| N-acetyl-isoputreanine | 0.0008 | 0.0051 | 0.9244 | 0.7019 | 0.0012 | 0.7019 | 0.0013 | 0.0076 | -0.271 | -0.246 | -0.086 | 0.841 | 1.018 | 1.137 | 2.161 | 2.124 | 1.901 | 1.117 | Amino Acid | Polyamine Metabolism | 131802901 | | | |
| N-acetyltryptophan | 0.0124 | 0.0478 | 0.2399 | 0.6895 | 0.0121 | 0.4463 | 0.1517 | 0.0369 | -0.318 | 0.071 | -0.201 | 0.625 | 1.309 | 1.084 | 1.922 | 1.468 | 1.773 | 0.828 | Amino Acid | Tryptophan Metabolism | 700653 | 1218-34-4 | C03137 | HMDB13713 |
| 2-oxoarginine* | 0 | 0.0004 | 0.0062 | 0.0002 | 0.0002 | 0.2183 | 0.2183 | 0.9901 | -0.685 | 0.088 | 0.475 | 0.472 | 1.709 | 2.236 | 2.23 | 1.305 | 0.997 | 1.308 | Amino Acid | Urea cycle; Arginine and Proline Metabolism | 558 | 10/4/3715 | C03771 | HMDB04225 |
| argininate* | 0.0088 | 0.0364 | 0.0505 | 0.0116 | 0.0475 | 0.4738 | 0.7376 | 0.6386 | -0.507 | 0.114 | 0.418 | 0.216 | 1.538 | 1.898 | 1.651 | 1.074 | 0.87 | 1.235 | Amino Acid | Urea cycle; Arginine and Proline Metabolism | 160437 | 157-07-3 | | HMDB03148 |
| dimethylarginine (ADMA + SDMA) | 0.0007 | 0.0044 | 0.3902 | 0.1279 | 0.0125 | 0.3902 | 0.0026 | 0.0005 | 0.003 | -0.224 | -0.482 | 0.797 | 0.854 | 0.715 | 1.735 | 2.03 | 2.428 | 0.836 | Amino Acid | Urea cycle; Arginine and Proline Metabolism | 123831 | 102783-24-4 | C03626 | HMDB01539 |
| homocitrulline | 0 | 0.0001 | 0.1729 | 0.2746 | 0 | 0.0291 | 0.0031 | 0 | -0.29 | 0.072 | -0.576 | 0.977 | 1.285 | 0.82 | 2.407 | 1.873 | 2.934 | 0.638 | Amino Acid | Urea cycle; Arginine and Proline Metabolism | 65072 | 1190-49-4 | C02427 | HMDB00679 |
| N-acetylcitrulline | 0.0059 | 0.0267 | 0.0094 | 0.0807 | 0.0524 | 0.506 | 0.671 | 0.7295 | -0.507 | 0.385 | 0.095 | 0.206 | 1.856 | 1.517 | 1.639 | 0.883 | 1.08 | 0.817 | Amino Acid | Urea cycle; Arginine and Proline Metabolism | 656979 | 33965-42-3 | C15532 | HMDB00856 |
| N-acetylhomocitrulline | 0 | 0 | 0.0015 | 0.4155 | 0 | 0.0295 | 0.0569 | 0.0002 | -0.556 | 0.301 | -0.343 | 0.856 | 1.811 | 1.159 | 2.659 | 1.468 | 2.296 | 0.64 | Amino Acid | Urea cycle; Arginine and Proline Metabolism | | | | |
| N-acetylproline | 0.0027 | 0.0137 | 0.933 | 0.0045 | 0.933 | 0.0046 | 0.933 | 0.0079 | 0.231 | 0.208 | -0.764 | 0.172 | 0.984 | 0.502 | 0.96 | 0.975 | 1.914 | 0.51 | Amino Acid | Urea cycle; Arginine and Proline Metabolism | 322640 | 1074-79-9 | | |
| N-delta-acetylornithine | 0.0112 | 0.0442 | 0.2055 | 0.0814 | 0.0086 | 0.4276 | 0.1418 | 0.3853 | -0.443 | -0.034 | 0.207 | 0.528 | 1.329 | 1.57 | 1.96 | 1.476 | 1.249 | 1.182 | Amino Acid | Urea cycle; Arginine and Proline Metabolism | 9920500 | | | |

| | | | | | | | | | | | | | | | | | | | | | | | | |
|---|---|---|---|---|---|---|---|---|---|---|---|---|---|---|---|---|---|---|---|---|---|---|---|---|
| N-methylproline | 0.0129 | 0.0488 | 0.243 | 0.0326 | 0.0211 | 0.243 | 0.1898 | 0.6858 | -0.437 | -0.071 | 0.318 | 0.449 | 1.289 | 1.688 | 1.848 | 1.433 | 1.095 | 1.309 | Amino Acid | Urea cycle; Arginine and Proline Metabolism | 557 | 475-11-6 | | |
| N,N,N-trimethyl-alanylproline betai | 0.0006 | 0.0043 | 0.6429 | 0.5268 | 0.002 | 0.6429 | 0.0012 | 0.001 | -0.106 | -0.232 | -0.367 | 0.857 | 0.917 | 0.835 | 1.95 | 2.127 | 2.335 | 0.911 | Amino Acid | Urea cycle; Arginine and Proline Metabolism | | | | |
| N2,N5-diacetylornithine | 0 | 0.0004 | 0.0831 | 0.0093 | 0 | 0.2775 | 0.0093 | 0.0831 | -0.565 | -0.087 | 0.222 | 0.775 | 1.393 | 1.726 | 2.532 | 1.817 | 1.467 | 1.239 | Amino Acid | Urea cycle; Arginine and Proline Metabolism | 65977 | 39825-23-5 | | |
| proline | 0.0008 | 0.0054 | 0.3172 | 0.0177 | 0.0057 | 0.1414 | 0.0491 | 0.5524 | 0.452 | 0.151 | -0.352 | -0.541 | 0.811 | 0.573 | 0.503 | 0.619 | 0.878 | 0.706 | Amino Acid | Urea cycle; Arginine and Proline Metabolism | 145742 | 147-85-3 | C00148 | HMDB00162 |
| urea | 0 | 0 | 0.0204 | 0.0479 | 0 | 0.7796 | 0.0005 | 0.0005 | -0.601 | 0.001 | -0.073 | 1.03 | 1.518 | 1.442 | 3.095 | 2.04 | 2.147 | 0.95 | Amino Acid | Urea cycle; Arginine and Proline Metabolism | 1176 | 57-13-6 | C00086 | HMDB00294 |
| erythronate* | 0 | 0 | 0.0354 | 0.0739 | 0 | 0.7875 | 0.0001 | 0.0001 | -0.582 | -0.042 | -0.112 | 1.098 | 1.454 | 1.385 | 3.204 | 2.204 | 2.313 | 0.953 | Carbohydrate | Aminosugar Metabolism | 2781043 | 88759-55-1 | | HMDB00613 |
| N-acetylglucosaminylasparagine | 0.0002 | 0.0021 | 0.043 | 0.043 | 0.0003 | 0.8834 | 0.0757 | 0.099 | -0.547 | 0.07 | 0.113 | 0.658 | 1.534 | 1.58 | 2.306 | 1.503 | 1.459 | 1.03 | Carbohydrate | Aminosugar Metabolism | 123826 | 2776-93-4 | C04540 | HMDB00489 |
| N-acetylneuraminate | 0.0014 | 0.008 | 0.1304 | 0.1304 | 0.0011 | 0.9773 | 0.0709 | 0.0709 | -0.447 | 0.005 | 0.014 | 0.688 | 1.368 | 1.376 | 2.196 | 1.606 | 1.596 | 1.006 | Carbohydrate | Aminosugar Metabolism | 439197 | 131-48-6 | C00270 | HMDB00230 |
| arabonate/xylonate | 0 | 0 | 0.2125 | 0.8574 | 0 | 0.2125 | 0 | 0 | -0.381 | -0.061 | -0.426 | 1.135 | 1.249 | 0.969 | 2.861 | 2.291 | 2.952 | 0.776 | Carbohydrate | Pentose Metabolism | | | | |
| ribonate | 0 | 0 | 0.0235 | 0.2766 | 0 | 0.2766 | 0.0003 | 0 | -0.54 | 0.04 | -0.268 | 1.09 | 1.496 | 1.208 | 3.095 | 2.07 | 2.562 | 0.808 | Carbohydrate | Pentose Metabolism | 5460677 | 8/3/5336 | C01685 | HMDB00867 |
| ribulonate/xylulonate/lyxonate* | 0.0003 | 0.0023 | 0.0403 | 0.0597 | 0.0001 | 0.8967 | 0.0348 | 0.0348 | -0.533 | 0.053 | 0.015 | 0.762 | 1.501 | 1.462 | 2.453 | 1.635 | 1.678 | 0.974 | Carbohydrate | Pentose Metabolism | | | | |
| 2-O-methylascorbic acid | 0 | 0.0002 | 0.0168 | 0.1788 | 0 | 0.3446 | 0.0167 | 0.0025 | -0.541 | 0.112 | -0.153 | 0.876 | 1.572 | 1.309 | 2.671 | 1.699 | 2.041 | 0.832 | Cofactors and | Ascorbate and Aldarate Metabolism | 99779 | 17860-87-6 | | |
| ascorbic acid 3-sulfate* | 0.0009 | 0.0055 | 0.6895 | 0.6895 | 0.0011 | 0.9931 | 0.0052 | 0.0056 | -0.302 | -0.146 | -0.144 | 0.821 | 1.114 | 1.116 | 2.178 | 1.955 | 1.951 | 1.002 | Cofactors and | Ascorbate and Aldarate Metabolism | 11425365 | | | |
| gulonate* | 0 | 0.0001 | 0.1979 | 0.0379 | 0 | 0.3624 | 0.0002 | 0.004 | -0.523 | -0.18 | 0.069 | 0.99 | 1.269 | 1.507 | 2.854 | 2.25 | 1.894 | 1.188 | Cofactors and | Ascorbate and Aldarate Metabolism | 152304 | 20246-53-1 | C00257 | HMDB03290 |
| N1-Methyl-2-pyridone-5-carboxami | 0 | 0.0001 | 0.6394 | 0.6808 | 0 | 0.5089 | 0 | 0.0004 | -0.235 | -0.391 | -0.126 | 1.013 | 0.897 | 1.079 | 2.376 | 2.647 | 2.202 | 1.202 | Cofactors and | Nicotinate and Nicotinamide Metabolism | 69698 | 701-44-0 | C05842 | HMDB04193 |
| nicotinamide | 0.013 | 0.0488 | 0.0462 | 0.892 | 0.0462 | 0.0783 | 0.9475 | 0.0783 | 0.355 | -0.357 | 0.26 | -0.378 | 0.61 | 0.936 | 0.602 | 0.986 | 0.643 | 1.534 | Cofactors and | Nicotinate and Nicotinamide Metabolism | 936 | 98-92-0 | C00153 | HMDB01406 |
| nicotinamide N-oxide | 0.0018 | 0.0096 | 0.0055 | 0.846 | 0.0334 | 0.0135 | 0.5703 | 0.0636 | 0.395 | -0.524 | 0.34 | -0.309 | 0.529 | 0.963 | 0.614 | 1.161 | 0.638 | 1.819 | Cofactors and | Nicotinate and Nicotinamide Metabolism | 72661 | 1986-81-8 | | HMDB02730 |
| quinolinate | 0 | 0.0002 | 0.3112 | 0.3771 | 0 | 0.8584 | 0.0005 | 0.0005 | -0.426 | -0.107 | -0.157 | 0.982 | 1.247 | 1.205 | 2.654 | 2.128 | 2.203 | 0.966 | Cofactors and | Nicotinate and Nicotinamide Metabolism | 1066 | 89-00-9 | C03722 | HMDB00232 |
| pterin | 0.0049 | 0.0231 | 0.1111 | 0.1111 | 0.0032 | 0.9734 | 0.1473 | 0.1473 | -0.481 | 0.086 | 0.076 | 0.573 | 1.481 | 1.471 | 2.075 | 1.401 | 1.411 | 0.993 | Cofactors and | Pterin Metabolism | 73000 | 22363-60-4 | C00715 | HMDB00802 |
| alpha-CEHC sulfate | 0.0015 | 0.0083 | 0.0057 | 0.0147 | 0.0147 | 0.829 | 0.829 | 0.9818 | 0.574 | -0.347 | -0.228 | -0.221 | 0.528 | 0.573 | 0.576 | 1.091 | 1.005 | 1.086 | Cofactors and | Tocopherol Metabolism | | | | |
| carotene diol (1) | 0.0003 | 0.0025 | 0.026 | 0.0022 | 0.0049 | 0.3164 | 0.4674 | 0.7206 | 0.587 | -0.085 | -0.454 | -0.342 | 0.628 | 0.486 | 0.525 | 0.837 | 1.081 | 0.774 | Cofactors and | Vitamin A Metabolism | | | | |
| pyridoxal | 0.0109 | 0.0437 | 0.0191 | 0.0372 | 0.2952 | 0.7661 | 0.266 | 0.2952 | 0.451 | -0.382 | -0.291 | 0.086 | 0.562 | 0.598 | 0.776 | 1.382 | 1.298 | 1.065 | Cofactors and | Vitamin B6 Metabolism | 1050 | 65-22-5 | C00250 | HMDB01545 |
| phosphate | 0.0029 | 0.0151 | 0.4399 | 0.1188 | 0.0022 | 0.3645 | 0.0153 | 0.1188 | -0.383 | -0.175 | 0.133 | 0.694 | 1.155 | 1.43 | 2.111 | 1.827 | 1.476 | 1.238 | Energy | Oxidative Phosphorylation | 1061 | 7664-38-2 | C00009 | HMDB01429 |
| aconitate [cis or trans] | 0 | 0 | 0.006 | 0.0018 | 0 | 0.4842 | 0.0018 | 0.0124 | -0.703 | 6E-04 | 0.185 | 0.92 | 1.629 | 1.851 | 3.079 | 1.891 | 1.664 | 1.137 | Energy | TCA Cycle | | | | |
| citrate | 0 | 0.0005 | 0.0211 | 0.0211 | 0.0001 | 0.8663 | 0.0494 | 0.0711 | -0.596 | 0.079 | 0.128 | 0.709 | 1.597 | 1.651 | 2.47 | 1.547 | 1.496 | 1.034 | Energy | TCA Cycle | 311 | 77-92-9 | C00158 | HMDB00094 |
| carnitine | 0.0004 | 0.0031 | 0.9682 | 0.772 | 0.0006 | 0.772 | 0.0006 | 0.0032 | -0.267 | -0.256 | -0.122 | 0.883 | 1.007 | 1.106 | 2.219 | 2.203 | 2.007 | 1.098 | Lipid | Carnitine Metabolism | 10917 | 461-05-2 | C00318 | HMDB00062 |
| adipoylcarnitine (C6-DC) | 0.0003 | 0.0025 | 0.7965 | 0.9621 | 0.0006 | 0.7965 | 0.0017 | 0.0011 | -0.274 | -0.135 | -0.261 | 0.885 | 1.101 | 1.009 | 2.232 | 2.027 | 2.212 | 0.916 | Lipid | Fatty Acid Metabolism (Acyl Carnitine, Dicarboxylate) | 71296139 | | | HMDB61677 |
| (R)-3-hydroxybutyrylcarnitine | 0.0001 | 0.0009 | 0.3628 | 0.0218 | 0.0004 | 0.1489 | 0.0059 | 0.1679 | -0.456 | -0.217 | 0.265 | 0.724 | 1.18 | 1.649 | 2.266 | 1.92 | 1.374 | 1.397 | Lipid | Fatty Acid Metabolism (Acyl Carnitine, Hydroxy) | 53481617 | | | HMDB13127 |
| (S)-3-hydroxybutyrylcarnitine | 0 | 0.0001 | 0.2131 | 0.1283 | 0 | 0.6627 | 0.001 | 0.0044 | -0.474 | -0.131 | -0.009 | 0.932 | 1.269 | 1.381 | 2.651 | 2.09 | 1.92 | 1.088 | Lipid | Fatty Acid Metabolism (Acyl Carnitine, Hydroxy) | | | | |
| 3-hydroxyhexanoylcarnitine (1) | 0.0023 | 0.0121 | 0.02 | 0.1737 | 0.0038 | 0.3524 | 0.3524 | 0.1424 | -0.502 | 0.247 | -0.049 | 0.53 | 1.68 | 1.369 | 2.044 | 1.216 | 1.493 | 0.815 | Lipid | Fatty Acid Metabolism (Acyl Carnitine, Hydroxy) | | | | |
| 3-hydroxypalmitoylcarnitine | 0.0043 | 0.0206 | 0.0317 | 0.0483 | 0.0039 | 0.8687 | 0.3441 | 0.3441 | -0.533 | 0.171 | 0.122 | 0.496 | 1.63 | 1.575 | 2.041 | 1.252 | 1.296 | 0.966 | Lipid | Fatty Acid Metabolism (Acyl Carnitine, Hydroxy) | | | | |
| arachidoylcarnitine (C20)* | 0 | 0.0005 | 0.0152 | 0.0035 | 0 | 0.454 | 0.0364 | 0.1696 | -0.648 | 0.045 | 0.256 | 0.699 | 1.617 | 1.871 | 2.544 | 1.573 | 1.359 | 1.157 | Lipid | Fatty Acid Metabolism (Acyl Carnitine, Long Chain Saturated) | | | | HMDB06460 |
| lignoceroylcarnitine (C24)* | 0.0002 | 0.0017 | 0.0181 | 0.0181 | 0.0001 | 0.7856 | 0.0672 | 0.1139 | -0.605 | 0.086 | 0.164 | 0.677 | 1.614 | 1.704 | 2.433 | 1.507 | 1.428 | 1.056 | Lipid | Fatty Acid Metabolism (Acyl Carnitine, Long Chain Saturated) | | | | |
| margaroylcarnitine (C17)* | 0.0062 | 0.0273 | 0.0405 | 0.0104 | 0.0104 | 0.55 | 0.55 | 0.9483 | -0.532 | 0.109 | 0.332 | 0.353 | 1.559 | 1.82 | 1.847 | 1.184 | 1.015 | 1.167 | Lipid | Fatty Acid Metabolism (Acyl Carnitine, Long Chain Saturated) | | 106182-29-0 | | HMDB06210 |
| palmitoylcarnitine (C16) | 0.001 | 0.0063 | 0.128 | 0.0151 | 0.0009 | 0.2866 | 0.0517 | 0.2954 | -0.523 | -0.063 | 0.284 | 0.612 | 1.376 | 1.75 | 2.196 | 1.596 | 1.255 | 1.272 | Lipid | Fatty Acid Metabolism (Acyl Carnitine, Long Chain Sat | 461 | 6865-14-1 | C02990 | HMDB00222 |
| stearoylcarnitine (C18) | 0.0001 | 0.001 | 0.0334 | 0.0069 | 0.0001 | 0.366 | 0.0334 | 0.1812 | -0.602 | -0.004 | 0.254 | 0.693 | 1.514 | 1.811 | 2.453 | 1.621 | 1.355 | 1.196 | Lipid | Fatty Acid Metabolism (Acyl Carnitine, Long Chain Sat | 6426855 | 18822-91-8 | | HMDB00848 |
| eicosenoylcarnitine (C20:1)* | 0 | 0.0005 | 0.0176 | 0.0176 | 0 | 0.9403 | 0.0262 | 0.0329 | -0.619 | 0.081 | 0.102 | 0.772 | 1.624 | 1.648 | 2.622 | 1.614 | 1.591 | 1.015 | Lipid | Fatty Acid Metabolism (Acyl Carnitine, Monounsaturated) | | | | |
| oleoylcarnitine (C18:1) | 0.0001 | 0.001 | 0.042 | 0.0284 | 0.0002 | 0.636 | 0.042 | 0.1138 | -0.567 | 0.03 | 0.167 | 0.685 | 1.513 | 1.663 | 2.382 | 1.575 | 1.433 | 1.1 | Lipid | Fatty Acid Metabolism (Acyl Carnitine, Monounsatura | 6441392 | 38677-66-6 | | HMDB05065 |
| arachidonoylcarnitine (C20:4) | 0.0001 | 0.0006 | 0.0285 | 0.024 | 0 | 0.7398 | 0.0226 | 0.0285 | -0.585 | 0.014 | 0.108 | 0.798 | 1.515 | 1.616 | 2.608 | 1.722 | 1.614 | 1.067 | Lipid | Fatty Acid Metabolism (Acyl Carnitine, Polyunsaturated) | | | | |
| dihomo-linolenoylcarnitine (C20:3n | 0.0035 | 0.017 | 0.2875 | 0.252 | 0.0018 | 0.7945 | 0.0354 | 0.0593 | -0.391 | -0.072 | 0.006 | 0.708 | 1.247 | 1.317 | 2.142 | 1.717 | 1.627 | 1.055 | Lipid | Fatty Acid Metabolism (Acyl Carnitine, Polyunsaturated) | | | | |
| dihomo-linoleoylcarnitine (C20:2)* | 0.0031 | 0.0156 | 0.283 | 0.1077 | 0.0019 | 0.4294 | 0.0381 | 0.1429 | -0.424 | -0.103 | 0.133 | 0.667 | 1.249 | 1.471 | 2.131 | 1.706 | 1.448 | 1.178 | Lipid | Fatty Acid Metabolism (Acyl Carnitine, Polyunsaturated) | | | | |
| linolenoylcarnitine (C18:3)* | 0.0117 | 0.0458 | 0.0431 | 0.0656 | 0.0198 | 0.8564 | 0.5991 | 0.5991 | -0.495 | 0.19 | 0.135 | 0.398 | 1.607 | 1.547 | 1.857 | 1.156 | 1.201 | 0.963 | Lipid | Fatty Acid Metabolism (Acyl Carnitine, Polyunsaturated) | | | | |
| acetylcarnitine (C2) | 0 | 0 | 0.2218 | 0.0399 | 0 | 0.343 | 0.0002 | 0.0055 | -0.514 | -0.184 | 0.076 | 0.973 | 1.257 | 1.505 | 2.802 | 2.23 | 1.862 | 1.198 | Lipid | Fatty Acid Metabolism (Acyl Carnitine, Short Chain) | 1 | 5080-50-2 | C02571 | HMDB00201 |
| butyrylcarnitine (C4) | 0.0002 | 0.0021 | 0.2735 | 0.2735 | 0.0002 | 0.8522 | 0.0078 | 0.0131 | -0.413 | -0.095 | -0.04 | 0.822 | 1.247 | 1.295 | 2.354 | 1.887 | 1.818 | 1.038 | Lipid | Fatty Acid Metabolism (also BCAA Metabolism) | 439829 | 25576-40-3 | C02862 | HMDB02013 |
| methylmalonate (MMA) | 0.0022 | 0.0113 | 0.8078 | 0.2056 | 0.0034 | 0.2724 | 0.0055 | 0.1132 | -0.319 | -0.253 | 0.108 | 0.719 | 1.047 | 1.344 | 2.054 | 1.963 | 1.528 | 1.285 | Lipid | Fatty Acid Metabolism (also BCAA Metabolism) | 487 | 516-05-2 | C02170 | HMDB00202 |
| propionylcarnitine (C3) | 0 | 0 | 0.016 | 0.0013 | 0 | 0.3049 | 0.0013 | 0.0176 | -0.679 | -0.055 | 0.218 | 0.919 | 1.542 | 1.862 | 3.028 | 1.964 | 1.626 | 1.208 | Lipid | Fatty Acid Metabolism (also BCAA Metabolism) | 107738 | 17298-37-2 | C03017 | HMDB00824 |
| malonate | 0.0004 | 0.003 | 0.0871 | 0.1334 | 0.0004 | 0.8455 | 0.0483 | 0.0483 | -0.472 | 0.037 | -0.02 | 0.722 | 1.424 | 1.368 | 2.288 | 1.607 | 1.673 | 0.961 | Lipid | Fatty Acid Synthesis | 867 | 141-82-2;265 | C00383 | HMDB00691 |
| 2-aminoheptanoate | 0.013 | 0.0488 | 0.2141 | 0.0388 | 0.0204 | 0.3865 | 0.2141 | 0.6345 | 0.453 | 0.017 | -0.285 | -0.439 | 0.739 | 0.6 | 0.539 | 0.729 | 0.899 | 0.811 | Lipid | Fatty Acid, Amino | 227939 | 1115-90-8 | | |
| azelate (C9-DC) | 0.0105 | 0.0424 | 0.3457 | 0.0327 | 0.0265 | 0.1953 | 0.1338 | 0.737 | 0.413 | 0.121 | -0.342 | -0.45 | 0.817 | 0.593 | 0.55 | 0.673 | 0.928 | 0.726 | Lipid | Fatty Acid, Dicarboxylate | 2266 | 123-99-9 | C08261 | HMDB00784 |
| maleate | 0 | 0.0003 | 0.1357 | 0.0425 | 0 | 0.492 | 0.0021 | 0.0158 | -0.519 | -0.114 | 0.079 | 0.889 | 1.324 | 1.513 | 2.654 | 2.005 | 1.754 | 1.143 | Lipid | Fatty Acid, Dicarboxylate | 444266 | 110-16-7 | C01384 | HMDB00176 |
| 2R,3R-dihydroxybutyrate | 0 | 0.0001 | 0.0469 | 0.0644 | 0 | 0.9158 | 0.0008 | 0.0008 | -0.565 | -0.032 | -0.06 | 1.002 | 1.447 | 1.418 | 2.962 | 2.047 | 2.088 | 0.98 | Lipid | Fatty Acid, Dihydroxy | 13120901 | 759-06-8 | | HMDB00498 |
| 2S,3R-dihydroxybutyrate | 0.0002 | 0.002 | 0.0527 | 0.0527 | 0.0001 | 0.89 | 0.0288 | 0.0372 | -0.525 | 0.006 | 0.045 | 0.779 | 1.445 | 1.485 | 2.469 | 1.709 | 1.663 | 1.028 | Lipid | Fatty Acid, Dihydroxy | 10964471 | 5057-93-2 | | HMDB02453 |
| 3,4-dihydroxybutyrate | 0 | 0.0004 | 0.0026 | 0.3436 | 0.0001 | 0.0505 | 0.2114 | 0.0026 | -0.535 | 0.329 | -0.279 | 0.719 | 1.821 | 1.195 | 2.386 | 1.311 | 1.997 | 0.656 | Lipid | Fatty Acid, Dihydroxy | | 51267-44-8 | | |
| 2-hydroxynervonate* | 0.0082 | 0.0349 | 0.3324 | 0.0949 | 0.0113 | 0.3324 | 0.0968 | 0.3324 | -0.414 | -0.083 | 0.219 | 0.533 | 1.258 | 1.552 | 1.929 | 1.533 | 1.243 | 1.233 | Lipid | Fatty Acid, Monohydroxy | 5312783 | | | |
| 3-hydroxystearate | 0.0041 | 0.0196 | 0.1712 | 0.0153 | 0.0153 | 0.2874 | 0.2748 | 0.8669 | -0.481 | -0.007 | 0.349 | 0.403 | 1.389 | 1.778 | 1.846 | 1.329 | 1.038 | 1.281 | Lipid | Fatty Acid, Monohydroxy | 5282907 | 45261-96-9 | | |
| chiro-inositol | 0.0004 | 0.0031 | 0.4298 | 0.2154 | 0.0002 | 0.5643 | 0.003 | 0.016 | -0.402 | -0.161 | 0.006 | 0.841 | 1.182 | 1.327 | 2.368 | 2.004 | 1.785 | 1.123 | Lipid | Inositol Metabolism | | 643-12-9 | C19891 | HMDB34220 |
| 3-hydroxybutyrate (BHBA) | 0.0001 | 0.0009 | 0.0614 | 0.0016 | 0.0009 | 0.1747 | 0.1009 | 0.6975 | -0.59 | -0.017 | 0.407 | 0.527 | 1.488 | 1.996 | 2.169 | 1.458 | 1.087 | 1.342 | Lipid | Ketone Bodies | 441 | 625-72-9 | C01089 | HMDB00357 |
| acetoacetate | 0 | 0.0006 | 0.0061 | 0.0003 | 0.0003 | 0.2616 | 0.2655 | 0.9122 | -0.681 | 0.096 | 0.481 | 0.448 | 1.713 | 2.238 | 2.188 | 1.277 | 0.977 | 1.306 | Lipid | Ketone Bodies | 96 | 11/2/3483 | C00164 | HMDB00060 |
| docosatrienoate (22:3n3) | 0.004 | 0.0195 | 0.1596 | 0.0638 | 0.0044 | 0.4414 | 0.1189 | 0.3303 | -0.467 | -0.025 | 0.206 | 0.557 | 1.359 | 1.595 | 2.034 | 1.497 | 1.275 | 1.174 | Lipid | Long Chain Polyunsaturated Fatty Acid (n3 and n6) | 5312556 | 59708-86-0 | C16534 | HMDB02823 |
| hexadecatrienoate (16:3n3) | 0.0099 | 0.0408 | 0.7634 | 0.5277 | 0.0082 | 0.6416 | 0.0139 | 0.062 | 0.277 | 0.194 | 0.006 | -0.697 | 0.945 | 0.829 | 0.509 | 0.539 | 0.614 | 0.878 | Lipid | Long Chain Polyunsaturated Fatty Acid (n3 and n6) | 5312428 | 7561-64-0 | | |
| 1-palmitoyl-2-arachidonoyl-GPC (16 | 0.0004 | 0.0029 | 0.1453 | 0.0069 | 0.0004 | 0.1658 | 0.0244 | 0.3008 | -0.541 | -0.104 | 0.328 | 0.647 | 1.354 | 1.827 | 2.279 | 1.683 | 1.248 | 1.349 | Lipid | Phosphatidylcholine (PC) | 10747814 | 35418-58-7 | | HMDB07982 |
| 1-stearoyl-2-arachidonoyl-GPC (18: | 0.0012 | 0.0069 | 0.0146 | 0.0146 | 0.0018 | 0.8354 | 0.3611 | 0.4246 | -0.58 | 0.15 | 0.212 | 0.503 | 1.659 | 1.731 | 2.119 | 1.277 | 1.224 | 1.043 | Lipid | Phosphatidylcholine (PC) | 16219824 | 35418-59-8 | | HMDB08048 |
| 1-palmitoyl-2-oleoyl-GPI (16:0/18:1 | 0.0008 | 0.0049 | 0.0221 | 0.0168 | 0.0006 | 0.7086 | 0.16 | 0.2779 | -0.585 | 0.099 | 0.208 | 0.581 | 1.607 | 1.733 | 2.245 | 1.397 | 1.295 | 1.079 | Lipid | Phosphatidylinositol (PI) | | | | HMDB09783 |
| 1-stearoyl-2-arachidonoyl-GPI (18:0 | 0 | 0.0001 | 0.0001 | 0 | 0.0001 | 0.6339 | 0.7781 | 0.7527 | -0.772 | 0.273 | 0.493 | 0.351 | 2.064 | 2.403 | 2.179 | 1.056 | 0.907 | 1.164 | Lipid | Phosphatidylinositol (PI) | | 383907-33-3 | | HMDB09815 |
| 1-stearoyl-2-oleoyl-GPI (18:0/18:1)* | 0.0004 | 0.0031 | 0.0033 | 0.0129 | 0.0007 | 0.7039 | 0.4584 | 0.3518 | -0.628 | 0.255 | 0.145 | 0.513 | 1.845 | 1.709 | 2.206 | 1.196 | 1.291 | 0.927 | Lipid | Phosphatidylinositol (PI) | | | | |
| trimethylamine N-oxide | 0 | 0.0003 | 0.1084 | 0.0165 | 0 | 0.2992 | 0.0039 | 0.0462 | -0.549 | -0.115 | 0.178 | 0.833 | 1.351 | 1.655 | 2.607 | 1.93 | 1.575 | 1.225 | Lipid | Phospholipid Metabolism | 1145 | 1184-78-7 | C01104 | HMDB00925 |
| 1-(1-enyl-palmitoyl)-2-arachidonoyl | 0.002 | 0.0109 | 0.2298 | 0.0173 | 0.0035 | 0.1962 | 0.0532 | 0.4721 | -0.473 | -0.121 | 0.331 | 0.558 | 1.277 | 1.746 | 2.044 | 1.601 | 1.171 | 1.368 | Lipid | Plasmalogen | | | | HMDB11352 |

| | | | | | | | | | | | | | | | | | | | | | | | | |
|---|---|---|---|---|---|---|---|---|---|---|---|---|---|---|---|---|---|---|---|---|---|---|---|---|
| 1-(1-enyl-palmitoyl)-2-linoleoyl-GPC | 0.0078 | 0.0339 | 0.0235 | 0.0235 | 0.0601 | 0.9168 | 0.8727 | 0.8727 | 0.501 | -0.259 | -0.291 | -0.152 | 0.59 | 0.578 | 0.636 | 1.078 | 1.102 | 0.978 | Lipid | Plasmalogen | | | | HMDB11211 |
| 1-(1-enyl-palmitoyl)-2-palmitoyl-GP | 0.0005 | 0.0035 | 0.008 | 0.0145 | 0.0043 | 0.8707 | 0.6454 | 0.6454 | 0.587 | -0.238 | -0.19 | -0.423 | 0.564 | 0.583 | 0.496 | 0.88 | 0.851 | 1.034 | Lipid | Plasmalogen | 11146967 | | | HMDB11206 |
| 1-(1-enyl-stearoyl)-2-arachidonoyl-( | 0.0004 | 0.0029 | 0.6243 | 0.007 | 0.0016 | 0.0256 | 0.0055 | 0.5187 | -0.429 | -0.301 | 0.402 | 0.645 | 1.093 | 1.78 | 2.106 | 1.926 | 1.183 | 1.628 | Lipid | Plasmalogen | 9547058 | | | HMDB05779 |
| 1-(1-enyl-stearoyl)-2-oleoyl-GPE (P- | 0.0051 | 0.0238 | 0.1158 | 0.0235 | 0.0056 | 0.4621 | 0.1755 | 0.4907 | -0.505 | 0.014 | 0.274 | 0.494 | 1.433 | 1.717 | 2 | 1.395 | 1.165 | 1.198 | Lipid | Plasmalogen | | 144371-68-6 | | HMDB11375 |
| sphingomyelin (d18:1/18:1, d18:2/1 | 0.0051 | 0.0238 | 0.0031 | 0.1125 | 0.1232 | 0.2619 | 0.2494 | 0.8672 | 0.498 | -0.48 | -0.108 | -0.055 | 0.508 | 0.657 | 0.682 | 1.343 | 1.038 | 1.294 | Lipid | Sphingomyelins | 6443882 | 108392-10-5 | | HMDB12101 |
| sphingomyelin (d18:1/20:1, d18:2/2 | 0 | 0.0001 | 0.002 | 0.002 | 0.0002 | 0.7692 | 0.3749 | 0.4909 | 0.678 | -0.195 | -0.279 | -0.529 | 0.546 | 0.515 | 0.433 | 0.794 | 0.841 | 0.944 | Lipid | Sphingomyelins | | 222403-67-0 | | |
| sphingomyelin (d18:1/20:2, d18:2/2 | 0.0079 | 0.0339 | 0.011 | 0.011 | 0.2486 | 0.8256 | 0.293 | 0.2819 | 0.493 | -0.32 | -0.386 | 0.039 | 0.569 | 0.544 | 0.73 | 1.282 | 1.342 | 0.955 | Lipid | Sphingomyelins | | | | |
| sphingomyelin (d18:1/22:2, d18:2/2 | 0.0015 | 0.0085 | 0.0322 | 0.009 | 0.009 | 0.5751 | 0.5127 | 0.807 | 0.552 | -0.109 | -0.32 | -0.397 | 0.633 | 0.547 | 0.518 | 0.819 | 0.948 | 0.864 | Lipid | Sphingomyelins | | | | |
| sphingomyelin (d18:2/24:2)* | 0.0017 | 0.0094 | 0.0352 | 0.0103 | 0.0103 | 0.5573 | 0.5573 | 0.9734 | 0.54 | -0.115 | -0.349 | -0.338 | 0.635 | 0.54 | 0.544 | 0.857 | 1.007 | 0.85 | Lipid | Sphingomyelins | | | | |
| stearoyl sphingomyelin (d18:1/18:0 | 0.0036 | 0.0177 | 0.003 | 0.0903 | 0.0903 | 0.2883 | 0.2883 | 0.917 | 0.511 | -0.469 | -0.115 | -0.082 | 0.507 | 0.648 | 0.663 | 1.308 | 1.023 | 1.278 | Lipid | Sphingomyelins | 6453725 | 85187-10-6;85 | C00550 | HMDB01348 |
| 1-methylhypoxanthine | 0.0001 | 0.0009 | 0.1015 | 0.1015 | 0.0118 | 0.9323 | 0.0002 | 0.0002 | 0.062 | -0.391 | -0.415 | 0.847 | 0.731 | 0.719 | 1.724 | 2.358 | 2.398 | 0.983 | Nucleotide | Purine Metabolism, (Hypo)Xanthine/Inosine containin | 70765 | 1125-39-9 | | HMDB13141 |
| allantoin | 0 | 0 | 0.0404 | 0.1099 | 0 | 0.6889 | 0 | 0 | -0.572 | -0.053 | -0.155 | 1.141 | 1.434 | 1.335 | 3.278 | 2.287 | 2.455 | 0.931 | Nucleotide | Purine Metabolism, (Hypo)Xanthine/Inosine containin | 204 | 97-59-6 | C02350 | HMDB00462 |
| inosine | 0.0126 | 0.0482 | 0.131 | 0.3152 | 0.0846 | 0.0296 | 0.6619 | 0.0286 | -0.177 | 0.296 | -0.504 | 0.431 | 1.388 | 0.797 | 1.524 | 1.098 | 1.912 | 0.574 | Nucleotide | Purine Metabolism, (Hypo)Xanthine/Inosine containin | 6021 | 58-63-9 | C00294 | HMDB00195 |
| N1-methylinosine | 0.0019 | 0.0103 | 0.0878 | 0.0878 | 0.0923 | 0.9897 | 0.0035 | 0.0035 | 0.153 | -0.385 | -0.389 | 0.667 | 0.689 | 0.687 | 1.429 | 2.074 | 2.079 | 0.997 | Nucleotide | Purine Metabolism, (Hypo)Xanthine/Inosine containin | 65095 | 20245-33-4 | | HMDB02721 |
| urate | 0.008 | 0.0341 | 0.0194 | 0.2205 | 0.0158 | 0.3171 | 0.6254 | 0.2205 | -0.474 | 0.287 | -0.052 | 0.437 | 1.695 | 1.34 | 1.88 | 1.11 | 1.403 | 0.791 | Nucleotide | Purine Metabolism, (Hypo)Xanthine/Inosine containin | 1175 | 69-93-2;120K5 | C00366 | HMDB00289 |
| 7-methylguanine | 0.0005 | 0.0035 | 0.0535 | 0.1622 | 0.0535 | 0.6204 | 0.001 | 0.0042 | 0.116 | -0.451 | -0.306 | 0.724 | 0.675 | 0.746 | 1.524 | 2.258 | 2.042 | 1.106 | Nucleotide | Purine Metabolism, Guanine containing | 11361 | 578-76-7 | C02242 | HMDB00897 |
| N2,N2-dimethylguanosine | 0 | 0 | 0.1082 | 0.1014 | 0.0004 | 0.8234 | 0 | 0 | 0.006 | -0.412 | -0.472 | 1.024 | 0.749 | 0.718 | 2.025 | 2.705 | 2.821 | 0.959 | Nucleotide | Purine Metabolism, Guanine containing | 92919 | 2140-67-2 | | HMDB04824 |
| 5-hydroxymethylcytidine | 0.0104 | 0.0419 | 0.5052 | 0.0545 | 0.0545 | 0.0209 | 0.0209 | 0.9665 | -0.201 | -0.421 | 0.413 | 0.427 | 0.858 | 1.531 | 1.545 | 1.8 | 1.009 | 1.783 | Nucleotide | Pyrimidine Metabolism, Cytidine containing | | 19235-17-7 | | |
| 5-methyl-2'-deoxycytidine | 0.0046 | 0.0219 | 0.2715 | 0.0429 | 0.0098 | 0.2715 | 0.1053 | 0.4663 | 0.441 | 0.086 | -0.281 | -0.515 | 0.782 | 0.607 | 0.516 | 0.659 | 0.85 | 0.775 | Nucleotide | Pyrimidine Metabolism, Cytidine containing | 440055 | 838-07-3 | C03592 | HMDB02224 |
| N4-acetylcytidine | 0.0001 | 0.0013 | 0.5441 | 0.9035 | 0.0008 | 0.5752 | 0.0002 | 0.0011 | -0.145 | -0.381 | -0.179 | 0.914 | 0.849 | 0.977 | 2.084 | 2.454 | 2.132 | 1.151 | Nucleotide | Pyrimidine Metabolism, Cytidine containing | 107461 | 3768-18-1 | | HMDB05923 |
| 3-ureidopropionate | 0.0007 | 0.0045 | 0.76 | 0.239 | 0.0006 | 0.3385 | 0.0014 | 0.0321 | -0.34 | -0.259 | 0.056 | 0.817 | 1.057 | 1.316 | 2.23 | 2.109 | 1.695 | 1.244 | Nucleotide | Pyrimidine Metabolism, Uracil containing | 111 | 462-88-4 | C02642 | HMDB00026 |
| 5,6-dihydrouridine | 0 | 0.0001 | 0.9153 | 0.7941 | 0 | 0.7941 | 0 | 0.0002 | -0.313 | -0.286 | -0.166 | 1.042 | 1.019 | 1.107 | 2.558 | 2.512 | 2.31 | 1.087 | Nucleotide | Pyrimidine Metabolism, Uracil containing | 94312 | 5/4/5627 | | |
| pseudouridine | 0 | 0 | 0.9467 | 0.9467 | 0 | 0.9467 | 0 | 0 | -0.31 | -0.294 | -0.232 | 1.118 | 1.011 | 1.055 | 2.689 | 2.659 | 2.549 | 1.043 | Nucleotide | Pyrimidine Metabolism, Uracil containing | 15047 | 1445-07-4 | C02067 | HMDB00767 |
| bilirubin degradation product, C17H | 0.0061 | 0.0271 | 0.0085 | 0.1986 | 0.0149 | 0.252 | 0.8652 | 0.3069 | 0.507 | -0.387 | 0.029 | -0.335 | 0.538 | 0.718 | 0.558 | 1.037 | 0.777 | 1.334 | Partially Chara | Partially Characterized Molecules | | | | |
| gamma-glutamyl-alpha-lysine | 0.0129 | 0.0488 | 0.2492 | 0.1855 | 0.0175 | 0.6878 | 0.1855 | 0.2599 | -0.395 | -0.009 | 0.114 | 0.519 | 1.307 | 1.423 | 1.885 | 1.442 | 1.324 | 1.089 | Peptide | Gamma-glutamyl Amino Acid | 65254 | | | |
| gamma-glutamylglutamate | 0.0011 | 0.0065 | 0.0495 | 0.0088 | 0.0016 | 0.4632 | 0.1628 | 0.4672 | -0.564 | 0.042 | 0.298 | 0.526 | 1.522 | 1.817 | 2.129 | 1.398 | 1.171 | 1.194 | Peptide | Gamma-glutamyl Amino Acid | 92865 | 1116-22-9 | C05282 | HMDB11737 |
| gamma-glutamylisoleucine* | 0.0011 | 0.0066 | 0.1629 | 0.0838 | 0.0024 | 0.5384 | 0.0838 | 0.1629 | -0.451 | -0.044 | 0.14 | 0.625 | 1.325 | 1.506 | 2.107 | 1.59 | 1.399 | 1.136 | Peptide | Gamma-glutamyl Amino Acid | 14253342 | | | HMDB11170 |
| gamma-glutamylphenylalanine | 0.0053 | 0.0241 | 0.5712 | 0.6723 | 0.008 | 0.8137 | 0.037 | 0.037 | -0.298 | -0.057 | -0.128 | 0.682 | 1.182 | 1.125 | 1.971 | 1.669 | 1.753 | 0.952 | Peptide | Gamma-glutamyl Amino Acid | 111299 | 7432-24-8 | | HMDB00594 |
| gamma-glutamyltyrosine | 0.0103 | 0.0419 | 0.69 | 0.0537 | 0.4532 | 0.1087 | 0.3467 | 0.0224 | 0.127 | 0.017 | -0.58 | 0.391 | 0.926 | 0.613 | 1.201 | 1.296 | 1.96 | 0.661 | Peptide | Gamma-glutamyl Amino Acid | 94340 | 7432-23-7 | | HMDB11741 |
| N,N-dimethyl-pro-pro | 0.0001 | 0.0008 | 0.9006 | 0.9006 | 0.0001 | 0.9006 | 0.0003 | 0.0003 | -0.279 | -0.2 | -0.245 | 0.961 | 1.056 | 1.024 | 2.362 | 2.237 | 2.307 | 0.97 | Peptide | Modified Peptides | | | | |
| 2-methoxyhydroquinone sulfate (1) | 0.0048 | 0.0225 | 0.0696 | 0.0051 | 0.0807 | 0.2741 | 0.9521 | 0.2741 | 0.501 | -0.126 | -0.495 | -0.108 | 0.647 | 0.501 | 0.656 | 1.013 | 1.308 | 0.774 | Xenobiotics | Benzoate Metabolism | | | | |
| 4-vinylcatechol sulfate | 0.0008 | 0.005 | 0.0489 | 0.0055 | 0.0055 | 0.3775 | 0.3775 | 0.9059 | 0.55 | -0.065 | -0.365 | -0.402 | 0.653 | 0.531 | 0.517 | 0.791 | 0.974 | 0.812 | Xenobiotics | Benzoate Metabolism | | | | |
| 4-vinylphenol sulfate | 0.0003 | 0.0024 | 0.214 | 0.0044 | 0.0018 | 0.0855 | 0.0353 | 0.619 | 0.508 | 0.151 | -0.411 | -0.566 | 0.781 | 0.529 | 0.475 | 0.609 | 0.898 | 0.677 | Xenobiotics | Benzoate Metabolism | 6426766 | 2628-17-3 | C05627 | HMDB62775 |
| hippurate | 0.0021 | 0.0112 | 0.9024 | 0.9024 | 0.0032 | 0.9024 | 0.0032 | 0.0092 | -0.207 | -0.24 | -0.137 | 0.785 | 0.977 | 1.05 | 1.989 | 2.035 | 1.894 | 1.075 | Xenobiotics | Benzoate Metabolism | 464 | 495-69-2 | C01586 | HMDB00714 |
| S-(3-hydroxypropyl)mercapturic aci | 0 | 0 | 0.0808 | 0.0603 | 0 | 0.7491 | 0.0001 | 0.0003 | -0.558 | -0.118 | -0.034 | 1.075 | 1.357 | 1.438 | 3.102 | 2.286 | 2.157 | 1.06 | Xenobiotics | Chemical | 3371179 | 23127-40-4 | | |
| sulfate* | 0 | 0 | 0.017 | 0.0053 | 0 | 0.5407 | 0 | 0.0005 | -0.661 | -0.088 | 0.067 | 1.092 | 1.488 | 1.657 | 3.372 | 2.266 | 2.035 | 1.114 | Xenobiotics | Chemical | 1118 | 14808-79-8 | C00059 | HMDB01448 |
| triethanolamine | 0.0123 | 0.0478 | 0.1 | 0.0109 | 0.0634 | 0.3007 | 0.6291 | 0.5465 | -0.482 | 0.061 | 0.451 | 0.21 | 1.457 | 1.909 | 1.615 | 1.109 | 0.846 | 1.31 | Xenobiotics | Chemical | 7618 | 102-71-6 | C06771 | HMDB32538 |
| 2-aminophenol sulfate | 0.003 | 0.0153 | 0.0863 | 0.0016 | 0.1489 | 0.1489 | 0.625 | 0.0943 | 0.48 | -0.117 | -0.606 | 0.031 | 0.661 | 0.471 | 0.732 | 1.108 | 1.555 | 0.713 | Xenobiotics | Food Component/Plant | 181670 | | | HMDB61116 |
| ferulic acid 4-sulfate | 0.0052 | 0.024 | 0.2756 | 0.0093 | 0.0692 | 0.1156 | 0.368 | 0.411 | 0.436 | 0.071 | -0.51 | -0.245 | 0.776 | 0.519 | 0.624 | 0.804 | 1.202 | 0.669 | Xenobiotics | Food Component/Plant | 6305574 | 86321-29-1 | | HMDB29200 |
| gluconate | 0 | 0.0006 | 0.025 | 0.346 | 0 | 0.1977 | 0.0344 | 0.0021 | -0.493 | 0.16 | -0.236 | 0.826 | 1.572 | 1.195 | 2.495 | 1.587 | 2.089 | 0.76 | Xenobiotics | Food Component/Plant | 10690 | 527-07-1 | C00257 | HMDB00625 |
| homostachydrine* | 0.0056 | 0.0251 | 0.166 | 0.0026 | 0.0847 | 0.0847 | 0.5743 | 0.1984 | -0.462 | -0.027 | 0.589 | 0.144 | 1.352 | 2.072 | 1.523 | 1.126 | 0.735 | 1.533 | Xenobiotics | Food Component/Plant | 441447 | 1195-94-4 | C08283 | HMDB33433 |
| mannonate* | 0 | 0.0001 | 0.2345 | 0.2273 | 0 | 0.837 | 0.0003 | 0.0007 | -0.463 | -0.139 | -0.083 | 1.003 | 1.252 | 1.302 | 2.761 | 2.206 | 2.122 | 1.04 | Xenobiotics | Food Component/Plant | 3246006 | 642-99-9 | | |
| X - 11381 | 0.0001 | 0.0009 | 0.0774 | 0.0008 | 0.0002 | 0.0774 | 0.0272 | 0.5713 | -0.591 | -0.112 | 0.443 | 0.614 | 1.394 | 2.047 | 2.305 | 1.653 | 1.126 | 1.469 | | | | | | |
| X - 12097 | 0.0011 | 0.0066 | 0.4356 | 0.4356 | 0.0031 | 0.1833 | 0.0239 | 0.0011 | -0.198 | 0.01 | -0.448 | 0.776 | 1.155 | 0.841 | 1.964 | 1.701 | 2.336 | 0.728 | | | | | | |
| X - 12100 | 0.0076 | 0.0331 | 0.1789 | 0.1789 | 0.0072 | 0.9922 | 0.1626 | 0.1626 | -0.401 | 0.018 | 0.021 | 0.591 | 1.338 | 1.34 | 1.989 | 1.487 | 1.484 | 1.002 | | | | | | |
| X - 12101 | 0 | 0 | 0.0904 | 0.0639 | 0 | 0.7311 | 0.0002 | 0.0007 | -0.548 | -0.114 | -0.022 | 1.042 | 1.351 | 1.439 | 3.009 | 2.228 | 2.091 | 1.066 | | | | | | |
| X - 12117 | 0.0012 | 0.0067 | 0.0368 | 0.0584 | 0.0014 | 0.85 | 0.1953 | 0.1953 | -0.538 | 0.146 | 0.09 | 0.569 | 1.607 | 1.545 | 2.154 | 1.341 | 1.394 | 0.962 | | | | | | |
| X - 12127 | 0.0001 | 0.0008 | 0.5841 | 0.2304 | 0.0001 | 0.4561 | 0.0007 | 0.0113 | -0.377 | -0.235 | 0.018 | 0.883 | 1.103 | 1.314 | 2.393 | 2.169 | 1.821 | 1.191 | | | | | | |
| X - 12680 | 0.0002 | 0.002 | 0.0101 | 0.4344 | 0.0002 | 0.101 | 0.1355 | 0.0047 | -0.487 | 0.259 | -0.272 | 0.725 | 1.677 | 1.161 | 2.317 | 1.381 | 1.995 | 0.692 | | | | | | |
| X - 12689 | 0.0003 | 0.0023 | 0.0023 | 0.0089 | 0.0011 | 0.7233 | 0.6142 | 0.5077 | -0.636 | 0.276 | 0.174 | 0.47 | 1.882 | 1.753 | 2.152 | 1.144 | 1.228 | 0.931 | | | | | | |
| X - 13684 | 0.0123 | 0.0478 | 0.1239 | 0.5606 | 0.0107 | 0.3036 | 0.2412 | 0.0515 | -0.364 | 0.153 | -0.195 | 0.588 | 1.431 | 1.124 | 1.934 | 1.352 | 1.72 | 0.786 | | | | | | |
| X - 13695 | 0.0038 | 0.0183 | 0.0196 | 0.0164 | 0.0152 | 0.777 | 0.777 | 0.777 | 0.546 | -0.169 | -0.272 | -0.362 | 0.609 | 0.567 | 0.533 | 0.875 | 0.939 | 0.931 | | | | | | |
| X - 15461 | 0 | 0.0005 | 0.1038 | 0.0456 | 0 | 0.5896 | 0.0042 | 0.0194 | -0.524 | -0.082 | 0.07 | 0.864 | 1.358 | 1.509 | 2.615 | 1.926 | 1.733 | 1.111 | | | | | | |
| X - 16580 | 0.0084 | 0.0353 | 0.6211 | 0.4246 | 0.0075 | 0.6211 | 0.0269 | 0.0793 | -0.309 | -0.146 | 0.004 | 0.676 | 1.119 | 1.242 | 1.978 | 1.768 | 1.593 | 1.109 | | | | | | |
| X - 18345 | 0.0011 | 0.0067 | 0.0299 | 0.2727 | 0.0006 | 0.2727 | 0.1397 | 0.0283 | -0.479 | 0.178 | -0.145 | 0.684 | 1.578 | 1.261 | 2.239 | 1.419 | 1.776 | 0.799 | | | | | | |
| X - 22162 | 0 | 0 | 0.589 | 0.589 | 0 | 0.8635 | 0 | 0 | -0.399 | -0.235 | -0.189 | 1.138 | 1.121 | 1.157 | 2.902 | 2.589 | 2.509 | 1.032 | | | | | | |
| X - 23536 | 0.0009 | 0.0057 | 0.6397 | 0.7285 | 0.0011 | 0.7285 | 0.006 | 0.0045 | -0.304 | -0.092 | -0.206 | 0.82 | 1.158 | 1.07 | 2.18 | 1.882 | 2.037 | 0.924 | | | | | | |
| X - 23593 | 0.0085 | 0.0357 | 0.6381 | 0.6381 | 0.0071 | 0.9433 | 0.028 | 0.028 | -0.292 | -0.089 | -0.111 | 0.696 | 1.151 | 1.134 | 1.984 | 1.723 | 1.749 | 0.985 | | | | | | |
| X - 23659 | 0 | 0.0005 | 0.9814 | 0.9814 | 0.0001 | 0.9814 | 0.0002 | 0.0002 | -0.276 | -0.228 | -0.235 | 0.983 | 1.034 | 1.029 | 2.393 | 2.315 | 2.325 | 0.995 | | | | | | |
| X - 23665 | 0.003 | 0.0151 | 0.0478 | 0.0046 | 0.0463 | 0.3112 | 0.7459 | 0.4554 | -0.523 | 0.098 | 0.478 | 0.197 | 1.538 | 2.001 | 1.647 | 1.071 | 0.823 | 1.301 | | | | | | |
| X - 23739 | 0 | 0.0003 | 0.0538 | 0.2489 | 0 | 0.4786 | 0.0033 | 0.0009 | -0.505 | 0.028 | -0.168 | 0.943 | 1.447 | 1.263 | 2.728 | 1.885 | 2.161 | 0.873 | | | | | | |
| X - 24408 | 0.0134 | 0.0498 | 0.0631 | 0.0931 | 0.022 | 0.8484 | 0.5271 | 0.5271 | -0.475 | 0.172 | 0.113 | 0.412 | 1.565 | 1.503 | 1.849 | 1.181 | 1.23 | 0.96 | | | | | | |
| X - 24623 | 0 | 0 | 0.0002 | 0 | 0 | 0.2945 | 0.5033 | 0.6543 | -0.801 | 0.204 | 0.551 | 0.424 | 2.006 | 2.552 | 2.336 | 1.165 | 0.915 | 1.272 | | | | | | |
| X - 24728 | 0 | 0 | 0.0392 | 0.04 | 0 | 0.9794 | 0.0006 | 0.0008 | -0.585 | -0.04 | -0.033 | 1.017 | 1.459 | 1.466 | 3.035 | 2.08 | 2.07 | 1.005 | | | | | | |
| X - 25247 | 0.0003 | 0.0022 | 0.0659 | 0.0006 | 0.0082 | 0.0782 | 0.3639 | 0.3905 | 0.571 | 0.002 | -0.571 | -0.304 | 0.674 | 0.453 | 0.545 | 0.809 | 1.203 | 0.672 | | | | | | |
| X - 25419 | 0.0089 | 0.0369 | 0.016 | 0.016 | 0.0854 | 0.7266 | 0.6823 | 0.5751 | -0.516 | 0.263 | 0.369 | 0.088 | 1.716 | 1.846 | 1.52 | 0.886 | 0.823 | 1.076 | | | | | | |
| X - 25422 | 0 | 0 | 0.4007 | 0.4684 | 0 | 0.8553 | 0 | 0 | -0.453 | -0.201 | -0.246 | 1.24 | 1.191 | 1.154 | 3.233 | 2.715 | 2.802 | 0.969 | | | | | | |

| | | | | | | | | | | | | | | | | | | | | | | | | |
|---|---|---|---|---|---|---|---|---|---|---|---|---|---|---|---|---|---|---|---|---|---|---|---|---|
| X - 25794 | 0.0112 | 0.0442 | 0.0447 | 0.0447 | 0.0225 | 0.8551 | 0.6471 | 0.6739 | -0.494 | 0.144 | 0.199 | 0.387 | 1.556 | 1.617 | 1.842 | 1.184 | 1.139 | 1.039 | | | | | | |
| X - 25948 | 0 | 0 | 0.061 | 0.009 | 0 | 0.365 | 0 | 0.0012 | -0.611 | -0.148 | 0.088 | 1.069 | 1.378 | 1.623 | 3.203 | 2.324 | 1.974 | 1.177 | | | | | | |
| X - 26035 | 0 | 0 | 0.3293 | 0.0569 | 0 | 0.3124 | 0 | 0.0006 | -0.496 | -0.263 | 0.034 | 1.093 | 1.175 | 1.444 | 3.008 | 2.56 | 2.083 | 1.229 | | | | | | |
| | | | | | | | | | | | | | | | | | | | | | | | | |

**Table S2.** P value distributions from a bootstrap subsampling study

| | Resampling size | | | | Variation Explained | | P values | | | | | |
|---|---|---|---|---|---|---|---|---|---|---|---|---|
| Iteration | A | B1 | B2 | C/D | PC1 (%) | PC2 (%) | PC1 | PC2 | Age | BCS | Body weight | Sex |
| 1 | 10 | 10 | 10 | 10 | 15.2 | 8.6 | 0.001183 | 0.070508 | 0.06754 | 0.744694 | 0.0542404 | 0.392559 |
| 2 | 10 | 10 | 10 | 10 | 15.1 | 8.2 | 3.15E-06 | 0.146352 | 0.084529 | 0.098693 | 0.05361359 | 0.410112 |
| 3 | 10 | 10 | 10 | 10 | 12.4 | 9.3 | 0.00028 | 0.531539 | 0.223515 | 0.573529 | 0.07686274 | 0.969057 |
| 4 | 10 | 10 | 10 | 10 | 13.7 | 8.8 | 0.007622 | 0.21972 | 0.090911 | 0.505312 | 0.13676947 | 1 |
| 5 | 10 | 10 | 10 | 10 | 15.7 | 9 | 0.000325 | 0.24474 | 0.128654 | 0.566646 | 0.26767972 | 0.969057 |
| 6 | 10 | 10 | 10 | 10 | 14.5 | 9.1 | 0.003234 | 0.720436 | 0.208693 | 0.595038 | 0.10319423 | 0.642298 |
| 7 | 10 | 10 | 10 | 10 | 15.6 | 8.8 | 6.39E-05 | 0.033048 | 0.154502 | 0.142478 | 0.34166331 | 1 |
| 8 | 10 | 10 | 10 | 10 | 13.5 | 9.7 | 1.43E-05 | 0.681202 | 0.087986 | 0.033059 | 0.06187466 | 0.726949 |
| 9 | 10 | 10 | 10 | 10 | 16 | 8.3 | 6.89E-05 | 0.915567 | 0.086226 | 0.370308 | 0.47209743 | 0.600571 |
| 10 | 10 | 10 | 10 | 10 | 11.9 | 9.4 | 4.07E-05 | 0.884057 | 0.145653 | 0.141674 | 0.16059284 | 0.684285 |
| 11 | 10 | 10 | 10 | 10 | 14.5 | 9.6 | 0.006612 | 0.315633 | 0.113612 | 0.477426 | 0.08467992 | 1 |
| 12 | 10 | 10 | 10 | 10 | 14 | 9.5 | 0.000205 | 0.133699 | 0.093926 | 0.810124 | 0.10714826 | 1 |
| 13 | 10 | 10 | 10 | 10 | 12 | 9.4 | 4.06E-05 | 0.037046 | 0.184981 | 0.008503 | 0.05588512 | 0.783186 |
| 14 | 10 | 10 | 10 | 10 | 15.1 | 8.4 | 0.000394 | 0.061978 | 0.055667 | 0.047621 | 0.39344308 | 1 |
| 15 | 10 | 10 | 10 | 10 | 12.6 | 8.6 | 0.000257 | 0.096358 | 0.113514 | 0.227209 | 0.15256719 | 0.897606 |
| 16 | 10 | 10 | 10 | 10 | 12.4 | 8.4 | 0.001286 | 0.025318 | 0.101169 | 0.229236 | 0.07250139 | 0.726949 |
| 17 | 10 | 10 | 10 | 10 | 14.6 | 9 | 0.000156 | 0.203072 | 0.090936 | 0.128633 | 0.113736 | 1 |
| 18 | 10 | 10 | 10 | 10 | 15.5 | 9.4 | 0.001402 | 0.155934 | 0.10813 | 0.096057 | 0.12303757 | 1 |
| 19 | 10 | 10 | 10 | 10 | 14.5 | 9.4 | 0.000689 | 0.49528 | 0.181322 | 0.470677 | 0.38799428 | 0.969057 |
| 20 | 10 | 10 | 10 | 10 | 14.7 | 9.1 | 0.000772 | 0.240639 | 0.229444 | 0.907359 | 0.05166925 | 1 |
| 21 | 10 | 10 | 10 | 10 | 12.5 | 8.2 | 1.77E-05 | 0.419127 | 0.23555 | 0.282169 | 0.28843598 | 0.684285 |
| 22 | 10 | 10 | 10 | 10 | 13.2 | 7.9 | 1.38E-05 | 0.614165 | 0.075208 | 0.981894 | 0.0938096 | 0.684285 |
| 23 | 10 | 10 | 10 | 10 | 15.9 | 8.8 | 0.00156 | 0.523986 | 0.149735 | 0.255732 | 0.06590854 | 1 |
| 24 | 10 | 10 | 10 | 10 | 15.9 | 8.6 | 0.002623 | 0.141546 | 0.209909 | 0.661177 | 0.08030861 | 0.851798 |
| 25 | 10 | 10 | 10 | 10 | 15.1 | 8.6 | 0.000265 | 0.877152 | 0.05727 | 0.4875 | 0.0525548 | 0.970745 |
| 26 | 10 | 10 | 10 | 10 | 13 | 10.6 | 0.001401 | 0.582323 | 0.075549 | 0.011838 | 0.37898745 | 0.851798 |
| 27 | 10 | 10 | 10 | 10 | 16.1 | 9.9 | 0.000344 | 0.620809 | 0.221061 | 0.340364 | 0.14046016 | 1 |
| 28 | 10 | 10 | 10 | 10 | 14.1 | 9.6 | 0.000177 | 0.087658 | 0.111755 | 0.08738 | 0.26388862 | 0.627147 |
| 29 | 10 | 10 | 10 | 10 | 15.1 | 7.9 | 3.95E-05 | 0.074181 | 0.060841 | 0.063928 | 0.21598928 | 0.969057 |
| 30 | 10 | 10 | 10 | 10 | 14.6 | 9.3 | 0.000116 | 0.233741 | 0.054195 | 0.252148 | 0.22918415 | 0.715437 |
| 31 | 10 | 10 | 10 | 10 | 15.3 | 8.8 | 0.000138 | 0.029782 | 0.064164 | 0.442496 | 0.47058824 | 0.865456 |

| | | | | | | | | | | | | |
|---|---|---|---|---|---|---|---|---|---|---|---|---|
| 32 | 10 | 10 | 10 | 10 | 13.5 | 9.7 | 0.000242 | 0.095229 | 0.182337 | 0.183773 | 0.30995848 | 0.897606 |
| 33 | 10 | 10 | 10 | 10 | 13.9 | 8.8 | 0.000154 | 0.425447 | 0.145248 | 0.221992 | 0.09694064 | 0.31176 |
| 34 | 10 | 10 | 10 | 10 | 13.4 | 11 | 0.000156 | 0.254732 | 0.267763 | 0.22301 | 0.31390304 | 0.894795 |
| 35 | 10 | 10 | 10 | 10 | 15.3 | 7.8 | 4.2E-05 | 0.879759 | 0.062503 | 0.377616 | 0.20689479 | 0.688927 |
| 36 | 10 | 10 | 10 | 10 | 16.5 | 7.7 | 0.001003 | 0.450305 | 0.057179 | 0.361604 | 0.7555242 | 0.341394 |
| 37 | 10 | 10 | 10 | 10 | 15.1 | 9.1 | 0.000409 | 0.465285 | 0.065419 | 0.330447 | 0.74337872 | 0.641891 |
| 38 | 10 | 10 | 10 | 10 | 12.7 | 9.7 | 0.000126 | 0.085036 | 0.112987 | 0.683971 | 0.38250436 | 1 |
| 39 | 10 | 10 | 10 | 10 | 12.6 | 9.4 | 0.001613 | 0.14771 | 0.139354 | 0.137491 | 0.08632695 | 0.715437 |
| 40 | 10 | 10 | 10 | 10 | 13.5 | 9.4 | 0.002035 | 0.191266 | 0.077283 | 0.340364 | 0.07671969 | 0.889491 |
| 41 | 10 | 10 | 10 | 10 | 16 | 8.8 | 0.000328 | 0.167299 | 0.330831 | 0.37295 | 0.08435731 | 1 |
| 42 | 10 | 10 | 10 | 10 | 15.2 | 9.4 | 0.000193 | 0.642325 | 0.109656 | 0.809324 | 0.22263356 | 0.528081 |
| 43 | 10 | 10 | 10 | 10 | 15.1 | 8.8 | 0.000254 | 0.434908 | 0.053729 | 0.173974 | 0.48959048 | 0.678373 |
| 44 | 10 | 10 | 10 | 10 | 15.6 | 8.4 | 0.000154 | 0.327034 | 0.063962 | 0.334678 | 0.44338568 | 1 |
| 45 | 10 | 10 | 10 | 10 | 13.2 | 9.8 | 0.000406 | 0.539191 | 0.213572 | 0.240978 | 0.16933386 | 0.715437 |
| 46 | 10 | 10 | 10 | 10 | 15.2 | 8.4 | 0.000254 | 0.805446 | 0.104361 | 0.924353 | 0.19599014 | 0.894795 |
| 47 | 10 | 10 | 10 | 10 | 13.6 | 8.7 | 0.000418 | 0.021968 | 0.284257 | 0.386108 | 0.05787573 | 1 |
| 48 | 10 | 10 | 10 | 10 | 15.5 | 8.3 | 0.000305 | 0.86906 | 0.11038 | 0.52511 | 0.22808267 | 0.402566 |
| 49 | 10 | 10 | 10 | 10 | 14.6 | 9.4 | 0.000394 | 0.256649 | 0.175034 | 0.395091 | 0.07936651 | 0.410112 |
| 50 | 10 | 10 | 10 | 10 | 13.8 | 9.5 | 0.000261 | 0.038818 | 0.413948 | 0.259724 | 0.1325843 | 0.894795 |
| 51 | 10 | 10 | 10 | 10 | 14.4 | 9.3 | 0.000654 | 0.281038 | 0.257104 | 0.511467 | 0.42120478 | 0.684285 |
| 52 | 10 | 10 | 10 | 10 | 14.7 | 9 | 0.00017 | 0.605826 | 0.095164 | 0.063903 | 0.1220913 | 0.894795 |
| 53 | 10 | 10 | 10 | 10 | 14.3 | 9.6 | 0.000442 | 0.560091 | 0.1234 | 0.962178 | 0.58359931 | 0.662157 |
| 54 | 10 | 10 | 10 | 10 | 13.8 | 9.8 | 0.000849 | 0.136716 | 0.206222 | 0.221538 | 0.11333071 | 0.889491 |
| 55 | 10 | 10 | 10 | 10 | 14.5 | 9.6 | 7.21E-06 | 0.287108 | 0.057829 | 0.008774 | 0.32083612 | 0.897606 |
| 56 | 10 | 10 | 10 | 10 | 14.1 | 9.1 | 1.74E-07 | 0.428548 | 0.070605 | 0.091221 | 0.1405942 | 0.642298 |
| 57 | 10 | 10 | 10 | 10 | 15.8 | 9.9 | 3.07E-05 | 0.45433 | 0.190637 | 0.663969 | 0.35966529 | 1 |
| 58 | 10 | 10 | 10 | 10 | 15.8 | 8.9 | 0.002521 | 0.197514 | 0.074013 | 0.056241 | 0.82599288 | 0.969057 |
| 59 | 10 | 10 | 10 | 10 | 14 | 9.5 | 3.06E-05 | 0.487298 | 0.099617 | 0.104569 | 0.09521663 | 1 |
| 60 | 10 | 10 | 10 | 10 | 15.5 | 8.4 | 0.000363 | 0.457847 | 0.104035 | 0.37862 | 0.24428358 | 1 |
| 61 | 10 | 10 | 10 | 10 | 15 | 8.8 | 0.000475 | 0.334217 | 0.080959 | 0.528141 | 0.36510877 | 0.783186 |
| 62 | 10 | 10 | 10 | 10 | 15.4 | 8.7 | 0.000564 | 0.316927 | 0.062043 | 0.492867 | 0.05887165 | 0.678373 |
| 63 | 10 | 10 | 10 | 10 | 17.3 | 7.6 | 9.46E-05 | 0.001811 | 0.348 | 0.648411 | 0.58771005 | 1 |
| 64 | 10 | 10 | 10 | 10 | 14.4 | 9.6 | 1.14E-05 | 0.073108 | 0.36307 | 0.819518 | 0.301322 | 0.538008 |
| 65 | 10 | 10 | 10 | 10 | 14.5 | 10.1 | 0.000117 | 0.078575 | 0.159807 | 0.917639 | 0.07796497 | 0.538008 |

| | | | | | | | | | | | | | |
|---|---|---|---|---|---|---|---|---|---|---|---|---|---|
| 66 | 10 | 10 | 10 | 10 | 14.5 | 9.4 | 1.71E-07 | 0.293448 | 0.081376 | 0.017478 | 0.06093505 | 0.897606 |
| 67 | 10 | 10 | 10 | 10 | 14.1 | 8.9 | 0.002456 | 0.902474 | 0.123927 | 0.640149 | 0.15885904 | 0.969057 |
| 68 | 10 | 10 | 10 | 10 | 16.3 | 8.4 | 0.000224 | 0.153466 | 0.168878 | 0.095337 | 0.07261736 | 0.433148 |
| 69 | 10 | 10 | 10 | 10 | 14.1 | 8.2 | 0.000105 | 0.546562 | 0.593803 | 0.12315 | 0.20313042 | 0.970745 |
| 70 | 10 | 10 | 10 | 10 | 15.7 | 9.6 | 0.000254 | 0.581101 | 0.073972 | 0.358373 | 0.08900392 | 0.688927 |
| 71 | 10 | 10 | 10 | 10 | 15.5 | 9.4 | 2.14E-05 | 0.353216 | 0.081706 | 0.980579 | 0.05896502 | 0.715437 |
| 72 | 10 | 10 | 10 | 10 | 14 | 10 | 8.92E-05 | 0.113399 | 0.142311 | 0.475564 | 0.215377 | 0.889491 |
| 73 | 10 | 10 | 10 | 10 | 14 | 10.5 | 0.003044 | 0.775273 | 0.620981 | 0.535467 | 0.08665017 | 1 |
| 74 | 10 | 10 | 10 | 10 | 12.9 | 9 | 0.006832 | 0.094136 | 0.25126 | 0.20492 | 0.4452356 | 1 |
| 75 | 10 | 10 | 10 | 10 | 15.2 | 8.8 | 0.00158 | 0.958492 | 0.310557 | 0.975152 | 0.08521172 | 0.688927 |
| 76 | 10 | 10 | 10 | 10 | 17.2 | 9.4 | 0.000473 | 0.33756 | 0.070077 | 0.408795 | 0.12750215 | 1 |
| 77 | 10 | 10 | 10 | 10 | 14 | 9.5 | 0.020279 | 0.326104 | 0.139802 | 0.09557 | 0.61004381 | 1 |
| 78 | 10 | 10 | 10 | 10 | 14.4 | 9.2 | 0.00048 | 0.422001 | 0.052036 | 0.738764 | 0.15729539 | 0.684285 |
| 79 | 10 | 10 | 10 | 10 | 14.2 | 8 | 0.00048 | 0.508843 | 0.327702 | 0.236181 | 0.05579793 | 1 |
| 80 | 10 | 10 | 10 | 10 | 14.4 | 9.1 | 0.000237 | 0.464298 | 0.118501 | 0.47211 | 0.172807 | 0.688927 |
| 81 | 10 | 10 | 10 | 10 | 15.3 | 8.7 | 0.000119 | 0.314442 | 0.05842 | 0.188548 | 0.07249767 | 0.897606 |
| 82 | 10 | 10 | 10 | 10 | 14.1 | 9.1 | 4.37E-06 | 0.981317 | 0.073567 | 0.00651 | 0.30216675 | 1 |
| 83 | 10 | 10 | 10 | 10 | 14.6 | 9.3 | 0.00041 | 0.174179 | 0.41236 | 0.251083 | 0.43565649 | 0.894795 |
| 84 | 10 | 10 | 10 | 10 | 15.6 | 9.1 | 5.35E-05 | 0.439883 | 0.053241 | 0.838255 | 0.0749002 | 0.894795 |
| 85 | 10 | 10 | 10 | 10 | 16.9 | 9.2 | 2.19E-06 | 0.087995 | 0.092855 | 0.08585 | 0.06939476 | 0.476884 |
| 86 | 10 | 10 | 10 | 10 | 15.2 | 8.7 | 0.00064 | 0.296322 | 0.118572 | 0.637462 | 0.46922479 | 1 |
| 87 | 10 | 10 | 10 | 10 | 13.8 | 9.8 | 0.00558 | 0.088212 | 0.165974 | 0.118585 | 0.37273213 | 0.897606 |
| 88 | 10 | 10 | 10 | 10 | 14.3 | 8.6 | 0.000148 | 0.319417 | 0.076358 | 0.562747 | 0.06396015 | 1 |
| 89 | 10 | 10 | 10 | 10 | 14.8 | 9.2 | 4.06E-05 | 0.244381 | 0.066454 | 0.545455 | 0.10862026 | 0.263255 |
| 90 | 10 | 10 | 10 | 10 | 15.3 | 9.7 | 1.64E-05 | 0.473905 | 0.06168 | 0.270666 | 0.07483052 | 0.970745 |
| 91 | 10 | 10 | 10 | 10 | 15.3 | 8.5 | 2.35E-05 | 0.507405 | 0.071624 | 0.089346 | 0.14105494 | 0.479046 |
| 92 | 10 | 10 | 10 | 10 | 13.2 | 9.1 | 3.81E-05 | 0.220973 | 0.121585 | 0.913344 | 0.18565285 | 0.969057 |
| 93 | 10 | 10 | 10 | 10 | 13.2 | 9.5 | 0.000153 | 0.471931 | 0.233728 | 0.574434 | 0.5997494 | 1 |
| 94 | 10 | 10 | 10 | 10 | 15.2 | 9.1 | 7.25E-05 | 0.143725 | 0.118875 | 0.408491 | 0.0852853 | 0.715437 |
| 95 | 10 | 10 | 10 | 10 | 14 | 9.6 | 0.000372 | 0.22024 | 0.213461 | 0.076022 | 0.05756272 | 1 |
| 96 | 10 | 10 | 10 | 10 | 14.3 | 9.2 | 4.86E-05 | 0.068715 | 0.065379 | 0.142319 | 0.36611249 | 0.969057 |
| 97 | 10 | 10 | 10 | 10 | 14.6 | 9.6 | 0.001489 | 0.405458 | 0.284746 | 0.237545 | 0.28653984 | 0.969057 |
| 98 | 10 | 10 | 10 | 10 | 16 | 7.8 | 0.002198 | 0.688818 | 0.08227 | 0.306553 | 0.05294746 | 0.969057 |
| 99 | 10 | 10 | 10 | 10 | 13.6 | 8.6 | 8.16E-05 | 0.987409 | 0.455925 | 0.139163 | 0.14350678 | 0.969057 |

| | | | | | | | | | | | | |
|---|---|---|---|---|---|---|---|---|---|---|---|---|
| 100 | 10 | 10 | 10 | 10 | 15.1 | 9.5 | 0.003581 | 0.320797 | 0.4829 | 0.223621 | 0.06390499 | 0.865456 |
| 101 | 10 | 10 | 10 | 10 | 13.6 | 9.6 | 2.69E-05 | 0.664547 | 0.097649 | 0.774976 | 0.25580205 | 0.889491 |
| 102 | 10 | 10 | 10 | 10 | 15.9 | 8.5 | 0.000243 | 0.191024 | 0.083957 | 0.02569 | 0.22614173 | 0.31176 |
| 103 | 10 | 10 | 10 | 10 | 14.5 | 8.6 | 0.018878 | 0.443702 | 0.301467 | 0.448153 | 0.07691323 | 0.970745 |
| 104 | 10 | 10 | 10 | 10 | 13.4 | 8.2 | 1.06E-05 | 0.419548 | 0.089897 | 0.213152 | 0.07341288 | 0.969057 |
| 105 | 10 | 10 | 10 | 10 | 13.2 | 10.1 | 0.000289 | 0.065963 | 0.097564 | 0.630291 | 0.09526896 | 0.512969 |
| 106 | 10 | 10 | 10 | 10 | 16.2 | 8.5 | 6.53E-05 | 0.161891 | 0.085991 | 0.039021 | 0.16733769 | 0.392559 |
| 107 | 10 | 10 | 10 | 10 | 17.8 | 8.1 | 5.86E-05 | 0.070801 | 0.105814 | 0.676201 | 0.12424418 | 0.257296 |
| 108 | 10 | 10 | 10 | 10 | 15.1 | 9.2 | 0.000107 | 0.415658 | 0.235545 | 0.621019 | 0.15173325 | 0.688927 |
| 109 | 10 | 10 | 10 | 10 | 13.1 | 9.1 | 0.000162 | 0.310398 | 0.217101 | 0.713144 | 0.23555273 | 0.715437 |
| 110 | 10 | 10 | 10 | 10 | 15.9 | 9 | 0.004717 | 0.217724 | 0.120508 | 0.42088 | 0.19938548 | 0.969057 |
| 111 | 10 | 10 | 10 | 10 | 14.8 | 8.8 | 0.010558 | 0.579524 | 0.964871 | 0.44433 | 0.06656957 | 0.969057 |
| 112 | 10 | 10 | 10 | 10 | 14.9 | 9.2 | 0.00268 | 0.515505 | 0.141353 | 0.097721 | 0.31738884 | 0.715437 |
| 113 | 10 | 10 | 10 | 10 | 14.5 | 8.7 | 0.000312 | 0.496242 | 0.071241 | 0.077339 | 0.20759038 | 0.268815 |
| 114 | 10 | 10 | 10 | 10 | 16.8 | 8 | 7.3E-05 | 0.398824 | 0.059538 | 0.355868 | 0.16679195 | 1 |
| 115 | 10 | 10 | 10 | 10 | 15 | 9.1 | 5.82E-06 | 0.495541 | 0.054133 | 0.706159 | 0.101234 | 0.402566 |
| 116 | 10 | 10 | 10 | 10 | 13.9 | 9.1 | 0.001948 | 0.442849 | 0.12918 | 0.097516 | 0.12708777 | 0.969057 |
| 117 | 10 | 10 | 10 | 10 | 15.9 | 8.1 | 2.93E-05 | 0.646848 | 0.123174 | 0.338554 | 0.27514907 | 0.969057 |
| 118 | 10 | 10 | 10 | 10 | 14 | 9.1 | 0.001575 | 0.744077 | 0.261605 | 0.402183 | 0.47970205 | 0.662157 |
| 119 | 10 | 10 | 10 | 10 | 14.8 | 9.7 | 0.000137 | 0.305081 | 0.071689 | 0.0816 | 0.13170836 | 1 |
| 120 | 10 | 10 | 10 | 10 | 15.1 | 8.9 | 0.000892 | 0.520254 | 0.055641 | 0.722092 | 0.50134984 | 0.257296 |
| 121 | 10 | 10 | 10 | 10 | 12.1 | 9.4 | 7.7E-05 | 0.719244 | 0.132065 | 0.091963 | 0.13498011 | 0.970745 |
| 122 | 10 | 10 | 10 | 10 | 14.4 | 9.7 | 0.000733 | 0.485155 | 0.20321 | 0.225503 | 0.15632289 | 0.969057 |
| 123 | 10 | 10 | 10 | 10 | 15.9 | 9.5 | 4.33E-05 | 0.518932 | 0.050702 | 0.075641 | 0.1709285 | 0.433148 |
| 124 | 10 | 10 | 10 | 10 | 15.5 | 9.2 | 0.002367 | 0.109291 | 0.420117 | 0.468951 | 0.31566016 | 0.714444 |
| 125 | 10 | 10 | 10 | 10 | 14.8 | 7.6 | 0.001559 | 0.439432 | 0.109375 | 0.25135 | 0.13003736 | 0.835815 |
| 126 | 10 | 10 | 10 | 10 | 14.9 | 8.9 | 0.000464 | 0.404778 | 0.498675 | 0.975082 | 0.51133069 | 0.851798 |
| 127 | 10 | 10 | 10 | 10 | 14.6 | 10 | 0.002789 | 0.31662 | 0.235992 | 0.693804 | 0.11910832 | 0.894795 |
| 128 | 10 | 10 | 10 | 10 | 16.4 | 9.2 | 0.001918 | 0.444899 | 0.15178 | 0.45796 | 0.33128582 | 0.894795 |
| 129 | 10 | 10 | 10 | 10 | 13.3 | 8.6 | 7.19E-05 | 0.622149 | 0.229204 | 0.120184 | 0.34144364 | 1 |
| 130 | 10 | 10 | 10 | 10 | 14 | 9.4 | 0.000132 | 0.120847 | 0.197498 | 0.041169 | 0.39857238 | 0.33233 |
| 131 | 10 | 10 | 10 | 10 | 13.6 | 10 | 2.46E-06 | 0.27344 | 0.084509 | 0.065968 | 0.10362677 | 1 |
| 132 | 10 | 10 | 10 | 10 | 14.8 | 8.5 | 0.001209 | 0.164173 | 0.066371 | 0.452263 | 0.09354636 | 0.894795 |
| 133 | 10 | 10 | 10 | 10 | 13.6 | 9.5 | 0.006461 | 0.390771 | 0.254966 | 0.75516 | 0.05080845 | 1 |

| | | | | | | | | | | | | |
|---|---|---|---|---|---|---|---|---|---|---|---|---|
| 134 | 10 | 10 | 10 | 10 | 15 | 9.2 | 4.31E-05 | 0.167471 | 0.345398 | 0.370308 | 0.09081927 | 1 |
| 135 | 10 | 10 | 10 | 10 | 15.7 | 8.9 | 0.000685 | 0.516563 | 0.35946 | 0.34924 | 0.10834362 | 0.865456 |
| 136 | 10 | 10 | 10 | 10 | 15.3 | 8.7 | 0.003133 | 0.200024 | 0.0989 | 0.331615 | 0.09966473 | 0.715437 |
| 137 | 10 | 10 | 10 | 10 | 14.9 | 9.3 | 0.000427 | 0.197227 | 0.259996 | 0.779627 | 0.05413502 | 0.851798 |
| 138 | 10 | 10 | 10 | 10 | 14.6 | 8.5 | 0.002906 | 0.132122 | 0.088052 | 0.219674 | 0.38322332 | 0.783186 |
| 139 | 10 | 10 | 10 | 10 | 14.5 | 9.6 | 6.08E-05 | 0.354171 | 0.209144 | 0.053576 | 0.26338875 | 0.760758 |
| 140 | 10 | 10 | 10 | 10 | 16.8 | 9.4 | 7.35E-05 | 0.845769 | 0.078527 | 0.020836 | 0.09577757 | 1 |
| 141 | 10 | 10 | 10 | 10 | 13.8 | 8.7 | 1.32E-05 | 0.143165 | 0.124442 | 0.573579 | 0.50322351 | 1 |
| 142 | 10 | 10 | 10 | 10 | 14.6 | 9.8 | 0.000178 | 0.648473 | 0.156947 | 0.135034 | 0.46998433 | 0.851798 |
| 143 | 10 | 10 | 10 | 10 | 13.7 | 10 | 0.000405 | 0.630027 | 0.18562 | 0.390143 | 0.32291028 | 0.889491 |
| 144 | 10 | 10 | 10 | 10 | 13.9 | 9.2 | 1.37E-06 | 0.975025 | 0.085953 | 0.110633 | 0.11039127 | 0.969057 |
| 145 | 10 | 10 | 10 | 10 | 15.1 | 9 | 0.000117 | 0.221549 | 0.198389 | 0.043178 | 0.2562647 | 0.962884 |
| 146 | 10 | 10 | 10 | 10 | 14.7 | 7.9 | 9.4E-05 | 0.250029 | 0.18403 | 0.020797 | 0.53453113 | 0.894795 |
| 147 | 10 | 10 | 10 | 10 | 15.5 | 8.7 | 4.21E-05 | 0.119231 | 0.077658 | 0.699787 | 0.30758495 | 0.894795 |
| 148 | 10 | 10 | 10 | 10 | 15.7 | 9.5 | 0.005257 | 0.374197 | 0.179036 | 0.335657 | 0.12949277 | 0.715437 |
| 149 | 10 | 10 | 10 | 10 | 15 | 9.6 | 0.001478 | 0.42683 | 0.497232 | 0.146525 | 0.06685971 | 0.783186 |
| 150 | 10 | 10 | 10 | 10 | 12.9 | 9.5 | 4.13E-05 | 0.571919 | 0.116864 | 0.457238 | 0.16797602 | 0.528081 |
| 151 | 10 | 10 | 10 | 10 | 14.9 | 9.2 | 1.79E-05 | 0.460601 | 0.058254 | 0.470759 | 0.17656756 | 0.970745 |
| 152 | 10 | 10 | 10 | 10 | 13.7 | 9 | 1.57E-06 | 0.456605 | 0.078993 | 0.299433 | 0.0619239 | 0.969057 |
| 153 | 10 | 10 | 10 | 10 | 12.9 | 9.5 | 6.14E-05 | 0.49592 | 0.293273 | 0.399742 | 0.12967042 | 0.684285 |
| 154 | 10 | 10 | 10 | 10 | 13.5 | 10.4 | 0.000916 | 0.435152 | 0.061308 | 0.47211 | 0.18151395 | 1 |
| 155 | 10 | 10 | 10 | 10 | 14 | 9.3 | 0.000371 | 0.713523 | 0.075484 | 0.610703 | 0.25787576 | 0.715437 |
| 156 | 10 | 10 | 10 | 10 | 14.6 | 7.9 | 3.15E-05 | 0.172813 | 0.124056 | 0.235502 | 0.36213941 | 0.894795 |
| 157 | 10 | 10 | 10 | 10 | 14.8 | 8 | 0.003226 | 0.980795 | 0.207501 | 0.125296 | 0.49221026 | 0.715437 |
| 158 | 10 | 10 | 10 | 10 | 12.2 | 9.5 | 0.000267 | 0.723231 | 0.05655 | 0.681378 | 0.88597133 | 0.688927 |
| 159 | 10 | 10 | 10 | 10 | 16.7 | 9.5 | 0.000426 | 0.430747 | 0.080287 | 0.571388 | 0.20883689 | 0.627147 |
| 160 | 10 | 10 | 10 | 10 | 14.1 | 9.4 | 0.000853 | 0.493464 | 0.059358 | 0.517602 | 0.29057636 | 1 |
| 161 | 10 | 10 | 10 | 10 | 13.5 | 9.6 | 0.00197 | 0.546341 | 0.078239 | 0.04449 | 0.06610737 | 0.970745 |
| 162 | 10 | 10 | 10 | 10 | 13.6 | 9.5 | 0.00289 | 0.584132 | 0.064365 | 0.666215 | 0.35912909 | 1 |
| 163 | 10 | 10 | 10 | 10 | 14.2 | 9.5 | 0.000909 | 0.037671 | 0.271952 | 0.091144 | 0.14107279 | 0.662157 |
| 164 | 10 | 10 | 10 | 10 | 16.3 | 8.2 | 0.000219 | 0.019041 | 0.05523 | 0.230186 | 0.11773847 | 0.889491 |
| 165 | 10 | 10 | 10 | 10 | 15.3 | 10.5 | 1.8E-05 | 0.381673 | 0.075263 | 0.467145 | 0.3145502 | 0.894795 |
| 166 | 10 | 10 | 10 | 10 | 13.2 | 9.7 | 4.2E-06 | 0.984944 | 0.109485 | 0.00469 | 0.09022141 | 1 |
| 167 | 10 | 10 | 10 | 10 | 14 | 9.3 | 4.93E-06 | 0.235206 | 0.052064 | 0.317581 | 0.31297719 | 0.505903 |

| | | | | | | | | | | | | |
|---|---|---|---|---|---|---|---|---|---|---|---|---|
| 168 | 10 | 10 | 10 | 10 | 16.4 | 9.3 | 0.000335 | 0.145398 | 0.132363 | 0.010306 | 0.55693887 | 0.889491 |
| 169 | 10 | 10 | 10 | 10 | 14.6 | 9.5 | 6.58E-05 | 0.554826 | 0.061821 | 0.05972 | 0.06209674 | 0.391327 |
| 170 | 10 | 10 | 10 | 10 | 15.3 | 9.2 | 3.66E-05 | 0.536667 | 0.077176 | 0.175528 | 0.06406548 | 0.715437 |
| 171 | 10 | 10 | 10 | 10 | 15.2 | 8.8 | 1.41E-05 | 0.444034 | 0.081399 | 0.970642 | 0.41467559 | 0.894795 |
| 172 | 10 | 10 | 10 | 10 | 14.5 | 10.2 | 0.001262 | 0.189075 | 0.252783 | 0.198741 | 0.09562496 | 0.715437 |
| 173 | 10 | 10 | 10 | 10 | 12.8 | 8.1 | 4.76E-05 | 0.428104 | 0.335353 | 0.668028 | 0.16309763 | 1 |
| 174 | 10 | 10 | 10 | 10 | 13.7 | 10.7 | 0.001744 | 0.503042 | 0.156466 | 0.67134 | 0.06850823 | 0.476884 |
| 175 | 10 | 10 | 10 | 10 | 16.1 | 8.5 | 0.000531 | 0.068911 | 0.413662 | 0.651096 | 0.30585145 | 1 |
| 176 | 10 | 10 | 10 | 10 | 15.6 | 8.8 | 0.000375 | 0.218655 | 0.07021 | 0.364427 | 0.10402496 | 0.715437 |
| 177 | 10 | 10 | 10 | 10 | 15.6 | 8.7 | 0.00213 | 0.432228 | 0.052005 | 0.037878 | 0.14459604 | 0.715437 |

**Table S3.** Pearson's correlation analysis between significant metabolites and key echo variables.

| | P values | | | FDR | | | Correlation coefficients | | |
|---|---|---|---|---|---|---|---|---|---|
| **BIOCHEMICAL** | **nLVIDd** | **nLAD** | **LA/Ao** | **nLVIDd** | **nLAD** | **LA/Ao** | **nLVIDd** | **nLAD** | **LA/Ao** |
| hydroxyasparagine | 0.034912 | 0.000333 | 0.000129 | 0.143833 | 0.00308 | 0.002098 | 0.242359 | 0.40087 | 0.425254 |
| N,N-dimethylalanine | 0.197889 | 0.027048 | 0.116845 | 0.302856 | 0.048555 | 0.142811 | 0.149342 | 0.253639 | 0.18139 |
| N-acetylalanine | 0.221365 | 0.009516 | 0.0083 | 0.316871 | 0.024272 | 0.020344 | 0.141918 | 0.295651 | 0.300724 |
| N-acetylasparagine | 0.365624 | 0.152091 | 0.253786 | 0.446874 | 0.183343 | 0.291937 | 0.105232 | 0.165891 | 0.132523 |
| N-carbamoylalanine | 0.271966 | 0.985715 | 0.95614 | 0.35721 | 0.985715 | 0.961604 | 0.127611 | -0.00209 | -0.00641 |
| creatine | 0.002969 | 0.000139 | 0.001394 | 0.052205 | 0.002036 | 0.005706 | 0.336369 | 0.423345 | 0.360172 |
| alpha-ketoglutaramate* | 0.281813 | 0.005324 | 0.000491 | 0.367401 | 0.017064 | 0.003307 | 0.125042 | 0.316639 | 0.390294 |
| carboxyethyl-GABA | 0.203971 | 0.038676 | 0.015363 | 0.306828 | 0.060388 | 0.031078 | 0.147361 | 0.237701 | 0.277143 |
| glutamine | 0.051926 | 0.005333 | 0.037242 | 0.166163 | 0.017064 | 0.060691 | -0.22384 | -0.31658 | -0.23943 |
| N-acetylglutamate | 0.234305 | 0.045105 | 0.073372 | 0.319671 | 0.067275 | 0.098576 | 0.138061 | 0.230552 | 0.206588 |
| N-acetylglutamine | 0.417994 | 0.05001 | 0.009107 | 0.493738 | 0.072742 | 0.020823 | 0.094258 | 0.225645 | 0.29729 |
| 2-hydroxybutyrate/2-hydroxyisobutyrat | 0.231709 | 0.012126 | 0.011939 | 0.3186 | 0.02907 | 0.025315 | 0.138822 | 0.286431 | 0.287032 |
| glycine | 0.370873 | 0.008706 | 0.007557 | 0.450163 | 0.022532 | 0.019277 | -0.10409 | -0.29896 | -0.30416 |
| N-acetylserine | 0.483024 | 0.038892 | 0.065287 | 0.549395 | 0.060388 | 0.090476 | 0.081681 | 0.237444 | 0.21254 |
| N-acetylthreonine | 0.152862 | 0.001757 | 0.005674 | 0.258689 | 0.007587 | 0.016108 | 0.165584 | 0.353086 | 0.314407 |
| sarcosine | 0.038679 | 0.007617 | 0.029745 | 0.151278 | 0.020624 | 0.049859 | -0.2377 | -0.30387 | -0.24949 |
| 4-guanidinobutanoate | 0.01348 | 0.000188 | 0.000449 | 0.105949 | 0.002407 | 0.003307 | 0.282311 | 0.4157 | 0.39276 |
| 1-methyl-5-imidazoleacetate | 0.159687 | 0.014347 | 0.015564 | 0.264185 | 0.032372 | 0.031127 | 0.162914 | 0.279859 | 0.276624 |
| 1-methylhistidine | 0.00242 | 1.06E-05 | 6.31E-06 | 0.052205 | 0.000467 | 0.000278 | 0.342997 | 0.481579 | 0.492184 |
| 3-methylhistidine | 0.109993 | 0.005884 | 0.008309 | 0.238135 | 0.017551 | 0.020344 | 0.184809 | 0.31313 | 0.300687 |
| carnosine | 0.110949 | 0.012553 | 0.010546 | 0.238135 | 0.02907 | 0.023796 | 0.184322 | 0.285091 | 0.291776 |
| formiminoglutamate | 0.303264 | 0.034035 | 0.037864 | 0.389594 | 0.056511 | 0.061138 | 0.119645 | 0.243504 | 0.238672 |
| imidazole lactate | 0.387221 | 0.015425 | 0.012933 | 0.466787 | 0.032709 | 0.026468 | 0.1006 | 0.27698 | 0.283929 |
| N-acetyl-1-methylhistidine* | 0.013803 | 4.97E-05 | 4.57E-05 | 0.105949 | 0.001248 | 0.001006 | 0.281381 | 0.447917 | 0.449811 |
| N-acetyl-3-methylhistidine* | 0.225051 | 0.00364 | 0.003809 | 0.316871 | 0.013135 | 0.012036 | 0.140803 | 0.32963 | 0.32811 |
| 2-hydroxy-3-methylvalerate | 0.754811 | 0.572708 | 0.253197 | 0.772364 | 0.59834 | 0.291937 | -0.03642 | 0.065722 | 0.132686 |
| 3-hydroxy-2-ethylpropionate | 0.148748 | 0.015266 | 0.043699 | 0.254224 | 0.032709 | 0.068063 | 0.167237 | 0.277395 | 0.232039 |
| 3-hydroxyisobutyrate | 0.558203 | 0.277571 | 0.622313 | 0.602723 | 0.311162 | 0.651947 | 0.068215 | 0.126141 | 0.057407 |
| 3-methyl-2-oxobutyrate | 0.360137 | 0.164301 | 0.064631 | 0.443245 | 0.196714 | 0.090279 | 0.106435 | 0.161156 | 0.213048 |
| 3-methylglutaconate | 0.46797 | 0.030896 | 0.021788 | 0.539661 | 0.052285 | 0.03913 | 0.084505 | 0.247818 | 0.262855 |
| ethylmalonate | 0.129447 | 0.000517 | 0.000696 | 0.25033 | 0.003793 | 0.003952 | 0.175486 | 0.388879 | 0.380546 |

| | | | | | | | | | |
|---|---|---|---|---|---|---|---|---|---|
| isobutyrylcarnitine (C4) | 0.03057 | 0.001443 | 0.012872 | 0.141586 | 0.006513 | 0.026468 | 0.248287 | 0.359122 | 0.284114 |
| isovalerylcarnitine (C5) | 0.05525 | 0.004643 | 0.007555 | 0.170596 | 0.016023 | 0.019277 | 0.220821 | 0.321381 | 0.304169 |
| N-acetylisoleucine | 0.261995 | 0.061462 | 0.090872 | 0.349326 | 0.08451 | 0.11674 | 0.130276 | 0.215565 | 0.195314 |
| N-acetylleucine | 0.510527 | 0.450697 | 0.438147 | 0.565112 | 0.480743 | 0.476011 | 0.076639 | 0.087807 | 0.09025 |
| N-acetylvaline | 0.131139 | 0.019495 | 0.025161 | 0.25033 | 0.038796 | 0.043845 | 0.174727 | 0.26748 | 0.256755 |
| tiglyl carnitine (C5) | 0.116455 | 0.003402 | 0.024717 | 0.244 | 0.01274 | 0.043502 | 0.181581 | 0.331884 | 0.257517 |
| 2-aminoadipate | 0.205785 | 0.051292 | 0.40253 | 0.306933 | 0.073086 | 0.440033 | 0.146778 | 0.224427 | 0.097409 |
| 5-(galactosylhydroxy)-L-lysine | 0.042624 | 0.000568 | 0.001268 | 0.15337 | 0.003947 | 0.005441 | 0.233204 | 0.386296 | 0.363046 |
| 6-oxopiperidine-2-carboxylate | 0.12485 | 0.036109 | 0.316814 | 0.246895 | 0.057254 | 0.355154 | 0.177586 | 0.240834 | 0.116364 |
| N,N,N-trimethyl-5-aminovalerate | 0.004438 | 0.001044 | 0.00714 | 0.060081 | 0.005104 | 0.018757 | 0.32293 | 0.368818 | 0.306215 |
| N2-acetyl,N6-methyllysine | 0.087614 | 0.048259 | 0.050258 | 0.213507 | 0.071374 | 0.075602 | 0.197274 | 0.227349 | 0.225407 |
| N6,N6,N6-trimethyllysine | 0.663378 | 0.086233 | 0.250221 | 0.699129 | 0.111595 | 0.291937 | 0.050736 | 0.198122 | 0.133514 |
| N6,N6-dimethyllysine | 0.119815 | 0.081195 | 0.258388 | 0.246895 | 0.105854 | 0.295301 | 0.179956 | 0.20131 | 0.131257 |
| N6-acetyllysine | 0.30603 | 0.262403 | 0.45607 | 0.390299 | 0.297955 | 0.492444 | 0.118968 | 0.130166 | 0.086772 |
| N6-carboxyethyllysine | 0.021475 | 0.004927 | 0.003298 | 0.118896 | 0.016676 | 0.010952 | 0.263461 | 0.319333 | 0.332915 |
| pipecolate | 0.657513 | 0.574542 | 0.927073 | 0.697122 | 0.59834 | 0.937729 | 0.051678 | 0.065409 | 0.010676 |
| 2,3-dihydroxy-5-methylthio-4-pentenoa | 0.139257 | 0.000747 | 0.000747 | 0.253017 | 0.004405 | 0.004092 | 0.171185 | 0.378544 | 0.37854 |
| alpha-ketobutyrate | 0.231212 | 0.092058 | 0.082853 | 0.3186 | 0.11573 | 0.10964 | 0.138969 | 0.194615 | 0.200244 |
| methionine | 0.064928 | 0.010215 | 0.017519 | 0.181388 | 0.025323 | 0.033883 | -0.21282 | -0.29298 | -0.27186 |
| S-carboxyethylcysteine | 0.195963 | 0.02367 | 0.075103 | 0.302539 | 0.042948 | 0.100138 | 0.149978 | 0.25936 | 0.205382 |
| S-methylmethionine | 0.148779 | 0.005544 | 0.011932 | 0.254224 | 0.017118 | 0.025315 | -0.16722 | -0.31522 | -0.28705 |
| N-acetylphenylalanine | 0.14219 | 0.034994 | 0.045225 | 0.253017 | 0.056548 | 0.069821 | 0.169944 | 0.242253 | 0.230427 |
| 5-methylthioadenosine (MTA) | 0.2619 | 0.118116 | 0.067662 | 0.349326 | 0.144364 | 0.092314 | 0.130302 | 0.180773 | 0.210732 |
| N-acetyl-isoputreanine | 0.316991 | 0.006507 | 0.00383 | 0.398503 | 0.018775 | 0.012036 | 0.116321 | 0.309551 | 0.32793 |
| N-acetyltryptophan | 0.316533 | 0.111978 | 0.085372 | 0.398503 | 0.138789 | 0.112131 | 0.116431 | 0.183802 | 0.198656 |
| 2-oxoarginine* | 0.016254 | 0.000298 | 0.000483 | 0.105949 | 0.00308 | 0.003307 | 0.274885 | 0.403739 | 0.390768 |
| argininate* | 0.056854 | 0.089002 | 0.206999 | 0.172522 | 0.113509 | 0.246162 | 0.21942 | 0.196432 | 0.14639 |
| dimethylarginine (ADMA + SDMA) | 0.429621 | 0.183738 | 0.059445 | 0.501839 | 0.2185 | 0.085059 | 0.091932 | 0.154126 | 0.217222 |
| homocitrulline | 0.035141 | 0.023034 | 0.061361 | 0.143833 | 0.042228 | 0.087093 | 0.242064 | 0.260515 | 0.215647 |
| N,N,N-trimethyl-alanylproline betaine (T | 0.469137 | 0.056075 | 0.040928 | 0.539661 | 0.07771 | 0.064895 | 0.084284 | 0.220096 | 0.235091 |
| N2,N5-diacetylornithine | 0.011237 | 0.002851 | 0.008323 | 0.102971 | 0.011406 | 0.020344 | 0.289359 | 0.337694 | 0.300625 |
| N-acetylcitrulline | 0.494273 | 0.702994 | 0.87052 | 0.55409 | 0.723549 | 0.890764 | -0.0796 | 0.044451 | 0.019011 |
| N-acetylhomocitrulline | 0.132277 | 0.067916 | 0.063017 | 0.25033 | 0.090554 | 0.088728 | 0.17422 | 0.210542 | 0.214317 |
| N-acetylproline | 0.270269 | 0.076652 | 0.018252 | 0.35721 | 0.100677 | 0.034542 | -0.12806 | -0.20432 | -0.27018 |

| | | | | | | | | | |
|---|---|---|---|---|---|---|---|---|---|
| N-delta-acetylornithine | 0.000865 | 0.016012 | 0.069114 | 0.030444 | 0.033549 | 0.093569 | 0.374319 | 0.275487 | 0.209653 |
| N-methylproline | 0.018976 | 0.069644 | 0.231979 | 0.115165 | 0.092161 | 0.274016 | 0.268591 | 0.209263 | 0.138743 |
| proline | 0.159648 | 0.001767 | 0.006469 | 0.264185 | 0.007587 | 0.017515 | -0.16293 | -0.3529 | -0.30976 |
| urea | 0.01721 | 0.000515 | 0.011056 | 0.108176 | 0.003793 | 0.024323 | 0.272578 | 0.389002 | 0.289978 |
| erythronate* | 0.142322 | 0.002841 | 0.000894 | 0.253017 | 0.011406 | 0.004573 | 0.169889 | 0.337816 | 0.373374 |
| N-acetylglucosaminylasparagine | 0.392651 | 0.066052 | 0.05117 | 0.470113 | 0.089425 | 0.075833 | 0.099459 | 0.211951 | 0.224543 |
| N-acetylneuraminate | 0.039767 | 0.051907 | 0.041768 | 0.152151 | 0.073086 | 0.065635 | 0.236421 | 0.223852 | 0.234149 |
| arabonate/xylonate | 0.07999 | 0.011383 | 0.012376 | 0.207004 | 0.027826 | 0.025931 | 0.202096 | 0.288862 | 0.28564 |
| ribonate | 0.020687 | 0.001351 | 0.001197 | 0.118896 | 0.006259 | 0.005441 | 0.265022 | 0.361115 | 0.364748 |
| ribulonate/xylulonate/lyxonate* | 0.11089 | 0.029892 | 0.119663 | 0.238135 | 0.051078 | 0.145246 | 0.184352 | 0.249275 | 0.180029 |
| 2-O-methylascorbic acid | 0.208607 | 0.029606 | 0.002941 | 0.308528 | 0.051078 | 0.010562 | 0.145879 | 0.249698 | 0.336688 |
| ascorbic acid 3-sulfate* | 0.100891 | 0.001101 | 0.000267 | 0.232394 | 0.005239 | 0.00298 | 0.189615 | 0.36724 | 0.406692 |
| gulonate* | 0.001996 | 1.01E-05 | 2.98E-06 | 0.050192 | 0.000467 | 0.000175 | 0.349098 | 0.482706 | 0.506847 |
| N1-Methyl-2-pyridone-5-carboxamide | 0.006218 | 0.000997 | 0.000767 | 0.072953 | 0.005104 | 0.004092 | 0.311174 | 0.370164 | 0.377773 |
| nicotinamide | 0.886829 | 0.211328 | 0.114435 | 0.891897 | 0.248032 | 0.140843 | -0.0166 | -0.14502 | -0.18257 |
| nicotinamide N-oxide | 0.4916 | 0.863709 | 0.470743 | 0.55409 | 0.878687 | 0.502126 | 0.080093 | -0.02002 | -0.08398 |
| quinolinate | 0.015848 | 0.002254 | 0.000613 | 0.105949 | 0.009447 | 0.003718 | 0.275899 | 0.345254 | 0.384155 |
| pterin | 0.172486 | 0.211391 | 0.021045 | 0.275977 | 0.248032 | 0.038185 | 0.158125 | 0.145 | 0.264306 |
| alpha-CEHC sulfate | 0.680714 | 0.246267 | 0.48461 | 0.713129 | 0.283288 | 0.513803 | -0.04797 | -0.13462 | -0.08139 |
| carotene diol (1) | 0.088557 | 0.09094 | 0.179138 | 0.213507 | 0.115146 | 0.214478 | -0.1967 | -0.19527 | -0.15574 |
| pyridoxal | 0.558014 | 0.395883 | 0.733553 | 0.602723 | 0.427456 | 0.759444 | -0.06825 | -0.09879 | 0.039689 |
| phosphate | 0.010061 | 0.007183 | 0.053608 | 0.102971 | 0.020391 | 0.077975 | 0.293555 | 0.305999 | 0.222291 |
| aconitate [cis or trans] | 0.123472 | 0.000468 | 0.000348 | 0.246895 | 0.003746 | 0.003221 | 0.178228 | 0.391615 | 0.399681 |
| citrate | 0.556566 | 0.018899 | 0.006629 | 0.602723 | 0.038233 | 0.017678 | 0.068498 | 0.268757 | 0.308887 |
| carnitine | 0.033626 | 0.000583 | 0.003127 | 0.143833 | 0.003947 | 0.01079 | 0.244047 | 0.38554 | 0.334677 |
| adipoylcarnitine (C6-DC) | 0.104313 | 0.001009 | 0.00029 | 0.232394 | 0.005104 | 0.002999 | 0.18777 | 0.369811 | 0.404524 |
| (R)-3-hydroxybutyrylcarnitine | 0.104066 | 0.00774 | 0.010933 | 0.232394 | 0.02064 | 0.024323 | 0.187902 | 0.303285 | 0.290407 |
| (S)-3-hydroxybutyrylcarnitine | 0.029768 | 0.000326 | 0.000539 | 0.141586 | 0.00308 | 0.003389 | 0.249458 | 0.401369 | 0.387727 |
| 3-hydroxyhexanoylcarnitine (1) | 0.344853 | 0.02888 | 0.004498 | 0.427423 | 0.050325 | 0.013649 | 0.109848 | 0.250787 | 0.322469 |
| 3-hydroxypalmitoylcarnitine | 0.146751 | 0.051866 | 0.019992 | 0.254224 | 0.073086 | 0.036652 | 0.168052 | 0.223891 | 0.266439 |
| arachidoylcarnitine (C20)* | 0.000775 | 0.00037 | 3.2E-05 | 0.030444 | 0.003258 | 0.000806 | 0.377467 | 0.397994 | 0.457846 |
| lignoceroylcarnitine (C24)* | 0.023899 | 0.015375 | 0.004666 | 0.118896 | 0.032709 | 0.01392 | 0.258951 | 0.277111 | 0.321209 |
| margaroylcarnitine (C17)* | 0.082331 | 0.088182 | 0.103552 | 0.207004 | 0.113285 | 0.128346 | 0.200578 | 0.196928 | 0.188176 |
| palmitoylcarnitine (C16) | 0.048993 | 0.020082 | 0.005353 | 0.165262 | 0.038796 | 0.015445 | 0.226629 | 0.266254 | 0.31645 |

| | | | | | | | | | |
|---|---|---|---|---|---|---|---|---|---|
| stearoylcarnitine (C18) | 0.036854 | 0.021519 | 0.001982 | 0.147418 | 0.040291 | 0.007753 | 0.239905 | 0.263376 | 0.34932 |
| eicosenoylcarnitine (C20:1)* | 0.014802 | 0.008219 | 0.002346 | 0.105949 | 0.02159 | 0.008975 | 0.278623 | 0.301086 | 0.343988 |
| oleoylcarnitine (C18:1) | 0.005659 | 0.000223 | 3.21E-05 | 0.071137 | 0.002458 | 0.000806 | 0.314505 | 0.411289 | 0.45782 |
| arachidonoylcarnitine (C20:4) | 0.048226 | 0.000813 | 0.000271 | 0.165262 | 0.004519 | 0.00298 | 0.227381 | 0.376104 | 0.406287 |
| dihomo-linolenoylcarnitine (C20:3n3 or | 0.130694 | 0.001015 | 0.000909 | 0.25033 | 0.005104 | 0.004573 | 0.174926 | 0.36965 | 0.372861 |
| dihomo-linoleoylcarnitine (C20:2)* | 0.042178 | 0.034675 | 0.018129 | 0.15337 | 0.056548 | 0.034542 | 0.233694 | 0.242666 | 0.270461 |
| linolenoylcarnitine (C18:3)* | 0.16089 | 0.044012 | 0.053482 | 0.264185 | 0.067275 | 0.077975 | 0.162452 | 0.231705 | 0.222405 |
| acetylcarnitine (C2) | 0.003327 | 4.02E-05 | 9.75E-05 | 0.052205 | 0.001179 | 0.001906 | 0.33263 | 0.452743 | 0.432004 |
| butyrylcarnitine (C4) | 0.050651 | 0.000638 | 0.001054 | 0.165262 | 0.004162 | 0.005015 | 0.225033 | 0.382993 | 0.368531 |
| methylmalonate (MMA) | 0.0427 | 5.83E-05 | 0.000507 | 0.15337 | 0.001283 | 0.003307 | 0.233121 | 0.444197 | 0.389413 |
| propionylcarnitine (C3) | 0.00152 | 0.0002 | 0.005143 | 0.044585 | 0.002407 | 0.015086 | 0.357543 | 0.414163 | 0.317844 |
| malonate | 0.732932 | 0.020137 | 0.006371 | 0.754363 | 0.038796 | 0.017515 | 0.039785 | 0.266141 | 0.310307 |
| 2-aminoheptanoate | 0.227598 | 0.016842 | 0.09201 | 0.317915 | 0.034873 | 0.117346 | -0.14004 | -0.27345 | -0.19464 |
| azelate (C9-DC) | 0.113773 | 0.000751 | 0.000461 | 0.241254 | 0.004405 | 0.003307 | -0.1829 | -0.37839 | -0.39207 |
| maleate | 0.219343 | 0.00334 | 0.000245 | 0.316871 | 0.01274 | 0.00298 | 0.142535 | 0.332499 | 0.408893 |
| 2R,3R-dihydroxybutyrate | 0.223609 | 0.000205 | 0.001261 | 0.316871 | 0.002407 | 0.005441 | 0.141238 | 0.41349 | 0.363205 |
| 2S,3R-dihydroxybutyrate | 0.239932 | 0.006075 | 0.00911 | 0.324831 | 0.01782 | 0.020823 | 0.13643 | 0.311998 | 0.297278 |
| 3,4-dihydroxybutyrate | 0.501082 | 0.039115 | 0.100338 | 0.558167 | 0.060388 | 0.125244 | 0.078354 | 0.237183 | 0.189918 |
| 2-hydroxynervonate* | 0.136674 | 0.049048 | 0.087348 | 0.253017 | 0.071937 | 0.113039 | 0.172295 | 0.226575 | 0.197436 |
| 3-hydroxystearate | 0.184561 | 0.044751 | 0.051274 | 0.287458 | 0.067275 | 0.075833 | 0.153841 | 0.230922 | 0.224445 |
| chiro-inositol | 0.010586 | 0.003339 | 0.000695 | 0.102971 | 0.01274 | 0.003952 | 0.291631 | 0.332509 | 0.380603 |
| 3-hydroxybutyrate (BHBA) | 0.103815 | 0.012483 | 0.03874 | 0.232394 | 0.02907 | 0.061983 | 0.188036 | 0.285309 | 0.237625 |
| acetoacetate | 0.031798 | 0.050527 | 0.375131 | 0.1435 | 0.072892 | 0.417025 | 0.246542 | 0.22515 | 0.103173 |
| docosatrienoate (22:3n3) | 0.064455 | 0.02028 | 0.018504 | 0.181388 | 0.038796 | 0.034645 | 0.213185 | 0.265847 | 0.269625 |
| hexadecatrienoate (16:3n3) | 0.053443 | 0.004574 | 0.001239 | 0.167964 | 0.016023 | 0.005441 | -0.22244 | -0.3219 | -0.36373 |
| 1-palmitoyl-2-arachidonoyl-GPC (16:0/2 | 0.045181 | 0.052382 | 0.004392 | 0.159039 | 0.073168 | 0.01356 | 0.230472 | 0.223413 | 0.323287 |
| 1-stearoyl-2-arachidonoyl-GPC (18:0/20 | 0.176202 | 0.231356 | 0.016597 | 0.279383 | 0.267886 | 0.032821 | 0.156784 | 0.138926 | 0.274043 |
| 1-palmitoyl-2-oleoyl-GPI (16:0/18:1)* | 0.003559 | 0.003657 | 0.00037 | 0.052205 | 0.013135 | 0.003252 | 0.330381 | 0.329477 | 0.398049 |
| 1-stearoyl-2-arachidonoyl-GPI (18:0/20: | 0.102385 | 0.031504 | 0.003501 | 0.232394 | 0.052807 | 0.011412 | 0.188804 | 0.246954 | 0.330928 |
| 1-stearoyl-2-oleoyl-GPI (18:0/18:1)* | 0.023565 | 0.015328 | 0.002923 | 0.118896 | 0.032709 | 0.010562 | 0.25955 | 0.277233 | 0.336884 |
| trimethylamine N-oxide | 0.483842 | 0.035342 | 0.048675 | 0.549395 | 0.056548 | 0.074494 | 0.081529 | 0.241806 | 0.226939 |
| 1-(1-enyl-palmitoyl)-2-arachidonoyl-GPI | 0.163975 | 0.249085 | 0.03352 | 0.264767 | 0.284668 | 0.055313 | 0.161279 | 0.133832 | 0.244188 |
| 1-(1-enyl-palmitoyl)-2-linoleoyl-GPC (P- | 0.430555 | 0.306304 | 0.60755 | 0.501839 | 0.336935 | 0.640292 | -0.09175 | -0.1189 | -0.05985 |
| 1-(1-enyl-palmitoyl)-2-palmitoyl-GPC (P | 0.162113 | 0.014394 | 0.025891 | 0.264185 | 0.032372 | 0.044675 | -0.16198 | -0.27973 | -0.25553 |

| | | | | | | | | | |
|---|---|---|---|---|---|---|---|---|---|
| 1-(1-enyl-stearoyl)-2-arachidonoyl-GPE | 0.058957 | 0.035165 | 0.000409 | 0.175332 | 0.056548 | 0.003307 | 0.21763 | 0.242034 | 0.395323 |
| 1-(1-enyl-stearoyl)-2-oleoyl-GPE (P-18:0 | 0.009167 | 0.00733 | 0.001378 | 0.100839 | 0.020477 | 0.005706 | 0.297044 | 0.305266 | 0.360527 |
| sphingomyelin (d18:1/18:1, d18:2/18:0) | 0.655909 | 0.978139 | 0.967637 | 0.697122 | 0.983728 | 0.967637 | -0.05194 | 0.003196 | 0.004732 |
| sphingomyelin (d18:1/20:1, d18:2/20:0) | 0.079406 | 0.009681 | 0.001 | 0.207004 | 0.02434 | 0.004887 | -0.20248 | -0.29501 | -0.3701 |
| sphingomyelin (d18:1/20:2, d18:2/20:1, | 0.821416 | 0.949901 | 0.886364 | 0.83566 | 0.960819 | 0.901735 | -0.02632 | 0.007329 | -0.01667 |
| sphingomyelin (d18:1/22:2, d18:2/22:1, | 0.08524 | 0.027312 | 0.00848 | 0.211299 | 0.048555 | 0.020445 | -0.19874 | -0.25322 | -0.29993 |
| sphingomyelin (d18:2/24:2)* | 0.134687 | 0.063757 | 0.017343 | 0.252181 | 0.086986 | 0.033883 | -0.17316 | -0.21373 | -0.27227 |
| stearoyl sphingomyelin (d18:1/18:0) | 0.522859 | 0.690564 | 0.643704 | 0.575145 | 0.714937 | 0.670367 | -0.07442 | -0.04641 | -0.05391 |
| 1-methylhypoxanthine | 0.33906 | 0.022577 | 0.011645 | 0.423223 | 0.041827 | 0.025303 | 0.111166 | 0.26136 | 0.287989 |
| allantoin | 0.050705 | 0.000822 | 0.003092 | 0.165262 | 0.004519 | 0.01079 | 0.224981 | 0.3758 | 0.335043 |
| inosine | 0.411173 | 0.838581 | 0.277651 | 0.488962 | 0.858083 | 0.315269 | 0.095639 | 0.023757 | 0.12612 |
| N1-methylinosine | 0.297596 | 0.028681 | 0.006194 | 0.385124 | 0.050325 | 0.017305 | 0.121046 | 0.251089 | 0.311307 |
| urate | 0.880864 | 0.304036 | 0.050219 | 0.890989 | 0.336543 | 0.075602 | -0.01748 | 0.119456 | 0.225444 |
| 7-methylguanine | 0.073657 | 0.044392 | 0.055887 | 0.202557 | 0.067275 | 0.080624 | 0.206388 | 0.231301 | 0.220261 |
| N2,N2-dimethylguanosine | 0.059772 | 0.000693 | 0.000162 | 0.175332 | 0.004358 | 0.002375 | 0.21695 | 0.380664 | 0.419498 |
| 5-hydroxymethylcytidine | 0.096946 | 0.113368 | 0.029516 | 0.230573 | 0.13953 | 0.049859 | 0.191804 | 0.183105 | 0.249832 |
| 5-methyl-2'-deoxycytidine | 0.022595 | 0.018407 | 0.002405 | 0.118896 | 0.03767 | 0.009005 | -0.26133 | -0.26984 | -0.3432 |
| N4-acetylcytidine | 0.015579 | 0.005293 | 0.001721 | 0.105949 | 0.017064 | 0.006883 | 0.276585 | 0.316846 | 0.353726 |
| 3-ureidopropionate | 0.140595 | 0.005512 | 0.003296 | 0.253017 | 0.017118 | 0.010952 | 0.170617 | 0.315424 | 0.332936 |
| 5,6-dihydrouridine | 0.06458 | 0.000112 | 0.000477 | 0.181388 | 0.001973 | 0.003307 | 0.213087 | 0.428613 | 0.391131 |
| pseudouridine | 0.034663 | 9.23E-05 | 0.000325 | 0.143833 | 0.001804 | 0.003179 | 0.242681 | 0.433332 | 0.401467 |
| bilirubin degradation product, C17H18N | 0.219978 | 0.224553 | 0.087322 | 0.316871 | 0.261731 | 0.113039 | -0.14234 | -0.14095 | -0.19745 |
| gamma-glutamyl-alpha-lysine | 0.954182 | 0.328206 | 0.092838 | 0.954182 | 0.358784 | 0.11755 | 0.006702 | 0.113674 | 0.194158 |
| gamma-glutamylglutamate | 0.014769 | 0.007547 | 0.028637 | 0.105949 | 0.020624 | 0.048933 | 0.278711 | 0.304206 | 0.251156 |
| gamma-glutamylisoleucine* | 0.724806 | 0.486805 | 0.397606 | 0.750388 | 0.516131 | 0.437366 | 0.041045 | 0.080979 | 0.098427 |
| gamma-glutamylphenylalanine | 0.630797 | 0.268101 | 0.130104 | 0.676953 | 0.302472 | 0.156837 | 0.056014 | 0.128636 | 0.17519 |
| gamma-glutamyltyrosine | 0.180871 | 0.428901 | 0.818124 | 0.284226 | 0.460284 | 0.842046 | -0.15513 | -0.09208 | 0.026818 |
| N,N-dimethyl-pro-pro | 0.201517 | 0.012447 | 0.019395 | 0.30575 | 0.02907 | 0.035932 | 0.148155 | 0.285418 | 0.267691 |
| 2-methoxyhydroquinone sulfate (1) | 0.12251 | 0.14205 | 0.376744 | 0.246895 | 0.17242 | 0.417025 | -0.17868 | -0.17 | -0.10283 |
| 4-vinylcatechol sulfate | 0.143927 | 0.066763 | 0.033628 | 0.253312 | 0.089697 | 0.055313 | -0.16922 | -0.21141 | -0.24404 |
| 4-vinylphenol sulfate | 0.079482 | 0.01976 | 0.009057 | 0.207004 | 0.038796 | 0.020823 | -0.20243 | -0.26692 | -0.29749 |
| hippurate | 0.023544 | 0.005828 | 0.024017 | 0.118896 | 0.017551 | 0.042697 | 0.259587 | 0.313463 | 0.258742 |
| S-(3-hydroxypropyl)mercapturic acid (H | 0.011701 | 0.000126 | 0.000186 | 0.102971 | 0.002015 | 0.002523 | 0.287805 | 0.425756 | 0.415941 |
| sulfate* | 0.002729 | 1.94E-05 | 1.3E-05 | 0.052205 | 0.000682 | 0.000456 | 0.339116 | 0.468871 | 0.47741 |

| | | | | | | | | | |
|---|---|---|---|---|---|---|---|---|---|
| triethanolamine | 0.12108 | 0.548029 | 0.250928 | 0.246895 | 0.577563 | 0.291937 | 0.179354 | 0.069982 | 0.133317 |
| 2-aminophenol sulfate | 0.224079 | 0.29955 | 0.296367 | 0.316871 | 0.333676 | 0.334363 | -0.1411 | -0.12056 | -0.12135 |
| ferulic acid 4-sulfate | 0.081476 | 0.021157 | 0.008866 | 0.207004 | 0.040038 | 0.020823 | -0.20113 | -0.26409 | -0.29829 |
| gluconate | 0.711676 | 0.094655 | 0.094266 | 0.741154 | 0.118151 | 0.118506 | 0.043091 | 0.193107 | 0.193331 |
| homostachydrine* | 0.081726 | 0.339542 | 0.470584 | 0.207004 | 0.368885 | 0.502126 | 0.200967 | 0.111055 | 0.084011 |
| mannonate* | 0.02432 | 0.000452 | 0.000131 | 0.118896 | 0.003746 | 0.002098 | 0.258209 | 0.392601 | 0.424758 |

**Table S4.** Pearson's correlation analysis on carnitine, significant acylcarnitines and echo variables

| | **P values (w/ echo variables)** | | | **FDR (w/ echo variables)** | | |
|---|---|---|---|---|---|---|
| **Carnitine & acylcarnitines** | nLVIDd | nLAD | LA/Ao | nLVIDd | nLAD | LA/Ao |
| adipoylcarnitine (C6-DC) | 0.1043 | 0.001 | 0.0003 | 0.2324 | 0.0049 | 0.0028 |
| carnitine | 0.0336 | 0.0006 | 0.0031 | 0.1437 | 0.0039 | 0.0107 |
| acetylcarnitine (C2) | 0.0033 | 0 | 0.0001 | 0.0528 | 0 | 0.0016 |
| butyrylcarnitine (C4) | 0.0507 | 0.0006 | 0.0011 | 0.1652 | 0.0039 | 0.0052 |
| tiglyl carnitine (C5) | 0.1165 | 0.0034 | 0.0247 | 0.2441 | 0.0127 | 0.0435 |
| (S)-3-hydroxybutyrylcarnitine | 0.0298 | 0.0003 | 0.0005 | 0.1417 | 0.0028 | 0.0031 |
| isovalerylcarnitine (C5) | 0.0552 | 0.0046 | 0.0076 | 0.1704 | 0.0159 | 0.0194 |
| dihomo-linoleoylcarnitine (C20:2)* | 0.0422 | 0.0347 | 0.0181 | 0.1534 | 0.0565 | 0.0346 |
| dihomo-linolenoylcarnitine (C20:3n3 or 6)* | 0.1307 | 0.001 | 0.0009 | 0.2504 | 0.0049 | 0.0045 |
| margaroylcarnitine (C17)* | 0.0823 | 0.0882 | 0.1036 | 0.2069 | 0.1133 | 0.1284 |
| 3-hydroxyhexanoylcarnitine (1) | 0.3449 | 0.0289 | 0.0045 | 0.4275 | 0.0504 | 0.0137 |
| 3-hydroxypalmitoylcarnitine | 0.1468 | 0.0519 | 0.02 | 0.2543 | 0.0731 | 0.0367 |
| isobutyrylcarnitine (C4) | 0.0306 | 0.0014 | 0.0129 | 0.1417 | 0.0063 | 0.0264 |
| propionylcarnitine (C3) | 0.0015 | 0.0002 | 0.0051 | 0.044 | 0.0022 | 0.015 |
| palmitoylcarnitine (C16) | 0.049 | 0.0201 | 0.0054 | 0.1652 | 0.0388 | 0.0156 |
| (R)-3-hydroxybutyrylcarnitine | 0.1041 | 0.0077 | 0.0109 | 0.2324 | 0.0205 | 0.0243 |
| eicosenoylcarnitine (C20:1)* | 0.0148 | 0.0082 | 0.0023 | 0.1063 | 0.0215 | 0.0088 |
| arachidonoylcarnitine (C20:4) | 0.0482 | 0.0008 | 0.0003 | 0.1652 | 0.0044 | 0.0028 |
| stearoylcarnitine (C18) | 0.0369 | 0.0215 | 0.002 | 0.1476 | 0.0403 | 0.0078 |
| linolenoylcarnitine (C18:3)* | 0.1609 | 0.044 | 0.0535 | 0.2642 | 0.0673 | 0.078 |
| arachidoylcarnitine (C20)* | 0.0008 | 0.0004 | 0 | 0.0317 | 0.0035 | 0 |
| lignoceroylcarnitine (C24)* | 0.0239 | 0.0154 | 0.0047 | 0.1188 | 0.0327 | 0.014 |
| oleoylcarnitine (C18:1) | 0.0057 | 0.0002 | 0 | 0.0717 | 0.0022 | 0 |

| | **r (with echo variables)** | | |
|---|---|---|---|
| **Carnitine & acylcarnitines** | nLVIDd | nLAD | LA/Ao |
| adipoylcarnitine (C6-DC) | 0.19 | 0.37 | 0.4 |
| carnitine | 0.24 | 0.39 | 0.33 |
| acetylcarnitine (C2) | 0.33 | 0.45 | 0.43 |
| butyrylcarnitine (C4) | 0.23 | 0.38 | 0.37 |

| | | | |
|---|---|---|---|
| tiglyl carnitine (C5) | 0.18 | 0.33 | 0.26 |
| (S)-3-hydroxybutyrylcarnitine | 0.25 | 0.4 | 0.39 |
| isovalerylcarnitine (C5) | 0.22 | 0.32 | 0.3 |
| dihomo-linoleoylcarnitine (C20:2)* | 0.23 | 0.24 | 0.27 |
| dihomo-linolenoylcarnitine (C20:3n3 or 6)* | 0.17 | 0.37 | 0.37 |
| margaroylcarnitine (C17)* | 0.2 | 0.2 | 0.19 |
| 3-hydroxyhexanoylcarnitine (1) | 0.11 | 0.25 | 0.32 |
| 3-hydroxypalmitoylcarnitine | 0.17 | 0.22 | 0.27 |
| isobutyrylcarnitine (C4) | 0.25 | 0.36 | 0.28 |
| propionylcarnitine (C3) | 0.36 | 0.41 | 0.32 |
| palmitoylcarnitine (C16) | 0.23 | 0.27 | 0.32 |
| (R)-3-hydroxybutyrylcarnitine | 0.19 | 0.3 | 0.29 |
| eicosenoylcarnitine (C20:1)* | 0.28 | 0.3 | 0.34 |
| arachidonoylcarnitine (C20:4) | 0.23 | 0.38 | 0.41 |
| stearoylcarnitine (C18) | 0.24 | 0.26 | 0.35 |
| linolenoylcarnitine (C18:3)* | 0.16 | 0.23 | 0.22 |
| arachidoylcarnitine (C20)* | 0.38 | 0.4 | 0.46 |
| lignoceroylcarnitine (C24)* | 0.26 | 0.28 | 0.32 |
| oleoylcarnitine (C18:1) | 0.31 | 0.41 | 0.46 |

**Table S5.** Significant KEGG Pathways

| Pathways | Total | Hits | P val | FDR | Impact |
|---|---|---|---|---|---|
| Arginine Biosynthesis | 14 | 4 | 0.0024 | 0.20 | 0 |
| Synthesis and Degradation of Ketone Bodies | 5 | 2 | 0.0174 | 0.63 | 0.6 |
| Nicotinate and Nicotinamide Metabolism | 15 | 3 | 0.0252 | 0.63 | 0.19 |
| Histidine Metabolism | 16 | 3 | 0.0300 | 0.63 | 0.14 |
| Valine, Leucine and Isoleucine (BCAA) Biosynthesis | 8 | 2 | 0.0448 | 0.74 | 0 |

**Table S6.** Quantitative metabolite set enrichment analysis (qMSEA)

| Metabolite Sets | Total | Hits | P val | FDR |
|---|---|---|---|---|
| Methionine Metabolism | 43 | 15 | 0.000 | 0.000 |
| Pyrimidine Metabolism | 59 | 8 | 0.000 | 0.000 |
| Nicotinate and Nicotinamide Metabolism | 37 | 6 | 0.000 | 0.000 |
| Methylhistidine Metabolism | 4 | 2 | 0.000 | 0.001 |
| Transfer of Acetyl Groups into Mitochondria | 22 | 3 | 0.000 | 0.001 |
| Spermidine and Spermine Biosynthesis | 18 | 5 | 0.000 | 0.001 |
| Histidine Metabolism | 43 | 12 | 0.000 | 0.003 |
| Homocysteine Degradation | 9 | 4 | 0.000 | 0.004 |
| Glycine and Serine Metabolism | 59 | 16 | 0.001 | 0.006 |
| Arginine and Proline Metabolism | 53 | 10 | 0.001 | 0.010 |
| D-Arginine and D-Ornithine Metabolism | 11 | 1 | 0.002 | 0.013 |
| Beta-Alanine Metabolism | 34 | 10 | 0.002 | 0.014 |
| Aspartate Metabolism | 35 | 10 | 0.003 | 0.016 |
| Purine Metabolism | 74 | 14 | 0.004 | 0.023 |
| Ammonia Recycling | 32 | 10 | 0.004 | 0.023 |
| Porphyrin Metabolism | 40 | 1 | 0.005 | 0.025 |
| Threonine and 2-Oxobutanoate Degradation | 20 | 2 | 0.006 | 0.025 |
| Amino Sugar Metabolism | 33 | 2 | 0.006 | 0.025 |
| Warburg Effect | 58 | 7 | 0.006 | 0.026 |
| Betaine Metabolism | 21 | 5 | 0.007 | 0.028 |
| Lysine Degradation | 30 | 4 | 0.012 | 0.045 |
| Lactose Synthesis | 20 | 1 | 0.014 | 0.047 |
| Lactose Degradation | 9 | 1 | 0.014 | 0.047 |
| Tryptophan Metabolism | 60 | 10 | 0.014 | 0.048 |
| Citric Acid Cycle | 32 | 5 | 0.016 | 0.050 |
| Oxidation of Branched Chain Fatty Acids | 26 | 3 | 0.020 | 0.061 |
| Ketone Body Metabolism | 13 | 2 | 0.025 | 0.075 |
| Pantothenate and CoA Biosynthesis | 21 | 4 | 0.028 | 0.079 |
| Phenylacetate Metabolism | 9 | 3 | 0.029 | 0.080 |
| Butyrate Metabolism | 19 | 3 | 0.03 | 0.08 |
| Phosphatidylethanolamine Biosynthesis | 12 | 3 | 0.038 | 0.098 |
| Phenylalanine and Tyrosine Metabolism | 28 | 6 | 0.042 | 0.106 |
| Bile Acid Biosynthesis | 65 | 10 | 0.050 | 0.123 |

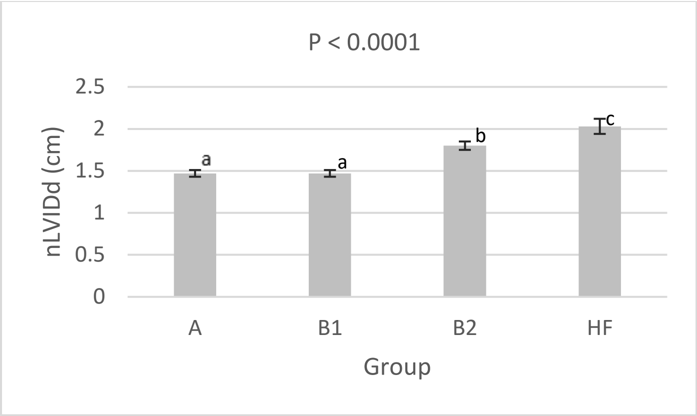
P < 0.0001
nLVIDd (cm)
2.5
2
1.5
1
0.5
0
a
a
b
c
A
B1
B2
HF
Group

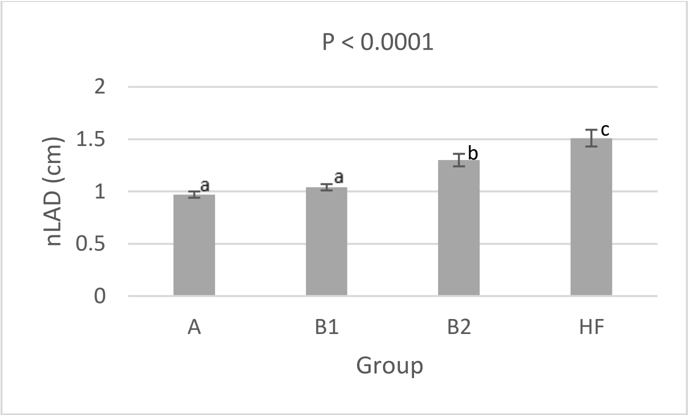
P < 0.0001
nLAD (cm)
2
1.5
1
0.5
0
a
a
b
c
A
B1
B2
HF
Group

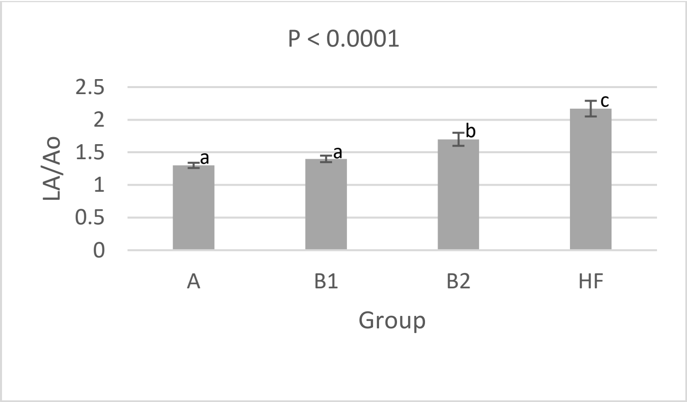
P < 0.0001
LA/Ao
2.5
2
1.5
1
0.5
0
a
a
b
c
A
B1
B2
HF
Group

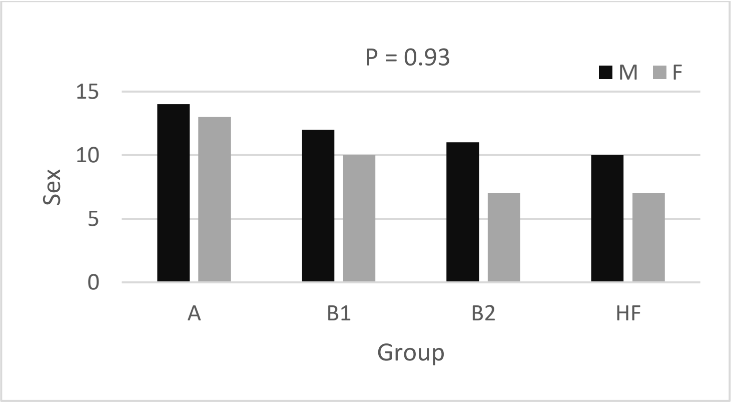
P = 0.93
M
F
Sex
15
10
5
0
A
B1
B2
HF
Group

**Figure S1.** Barplots of sex, age, body weights, BCS, and key echo variables. P value from ANOVA are displayed on the top of each barplot. Different letters indicate statistical difference.

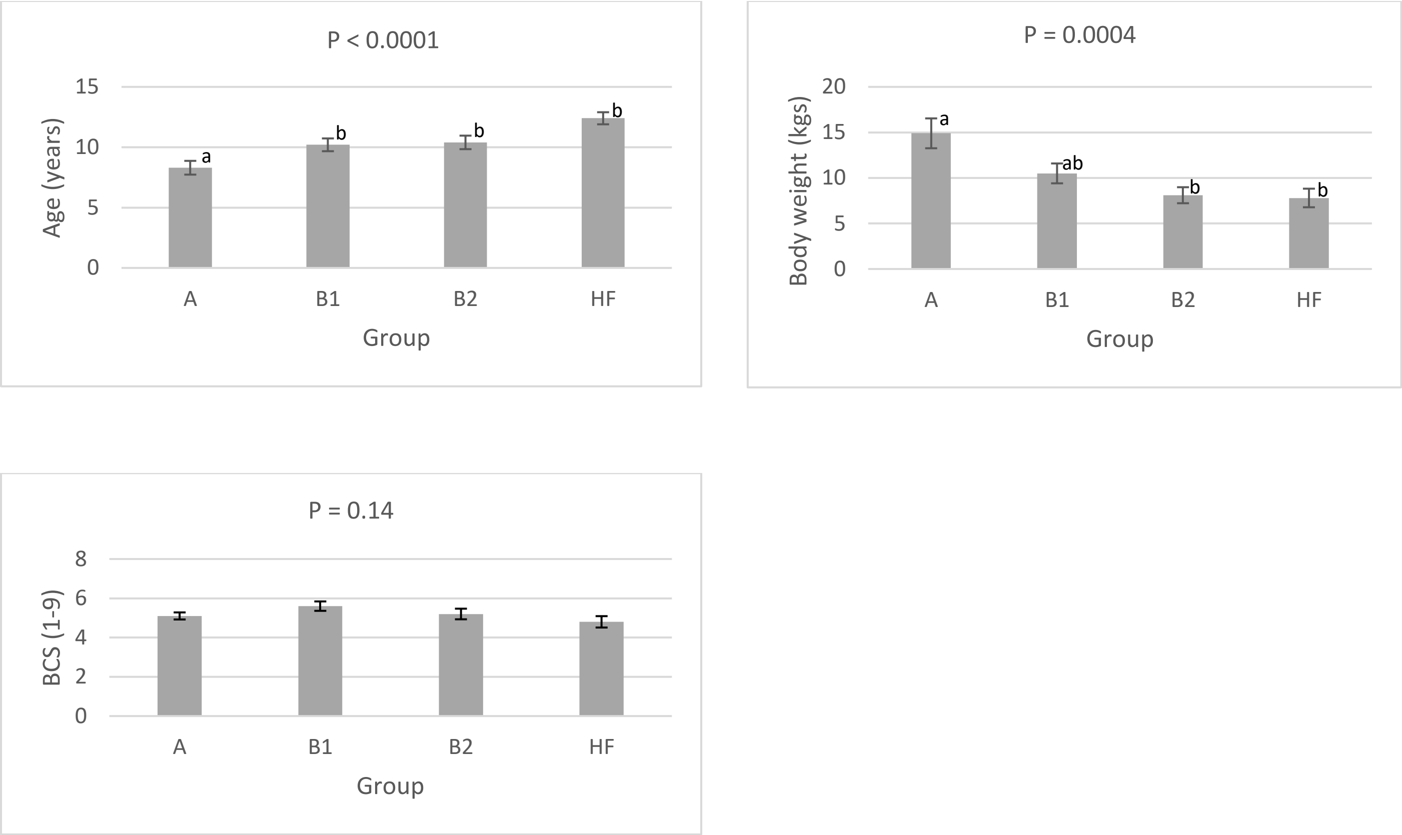

**Figure S2.** PLS-DA (top panel). The percentages of data variation explained by the first two components, are indicated on the x and y axes. Boxplots along component 1 (bottom panel). Tukey's test was performed to compare groups. *** P < 0.001; **** P < 0.0001.

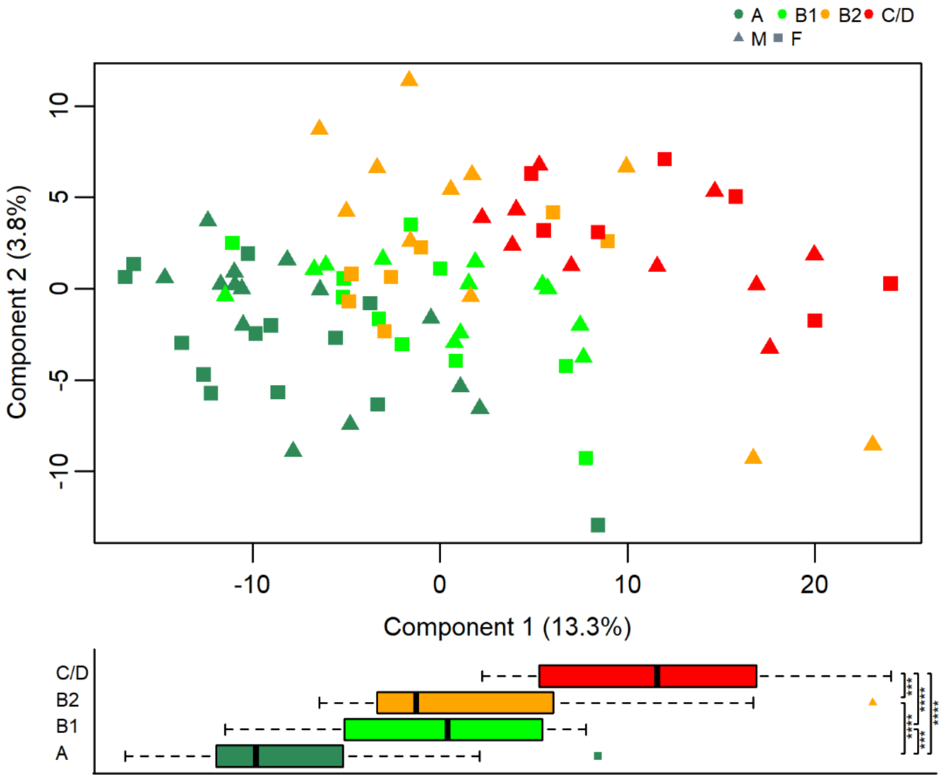

**Figure S3**. (A) Distribution of P values from bootstrap experiments. Only bootstrapped subsamples that showed no difference in age or body weight were analyzed. (Left)   was plotted against each sample on PC1, PC1, age, body weight (BW), sex and BCS. (Right) The histogram of  . The greyish dashed line denotes P = 0.05. (B) PCA analysis on cardiac medications. Only preclinical dogs in the B1 and B2 groups were included. Dogs were considered for two groups: those that were on stable cardiac medications for 2 weeks or longer (Y) and those that were not (N). Student's t-test was performed to compare the difference on PC1 or PC2 between groups.

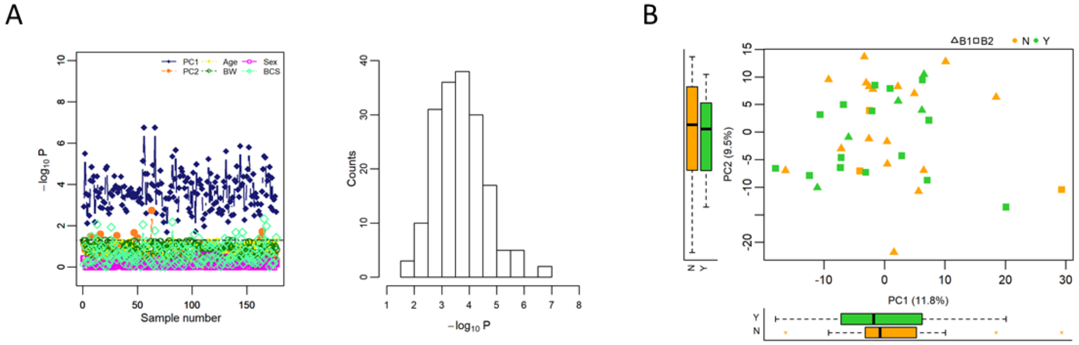

**Figure S4.** Heat map of 22 acylcarnitine concentrations from all samples. Hierarchical clustering was performed on the Euclidean distances between samples. The color scale corresponds to concentrations from low (deep blue) to high (maroon).

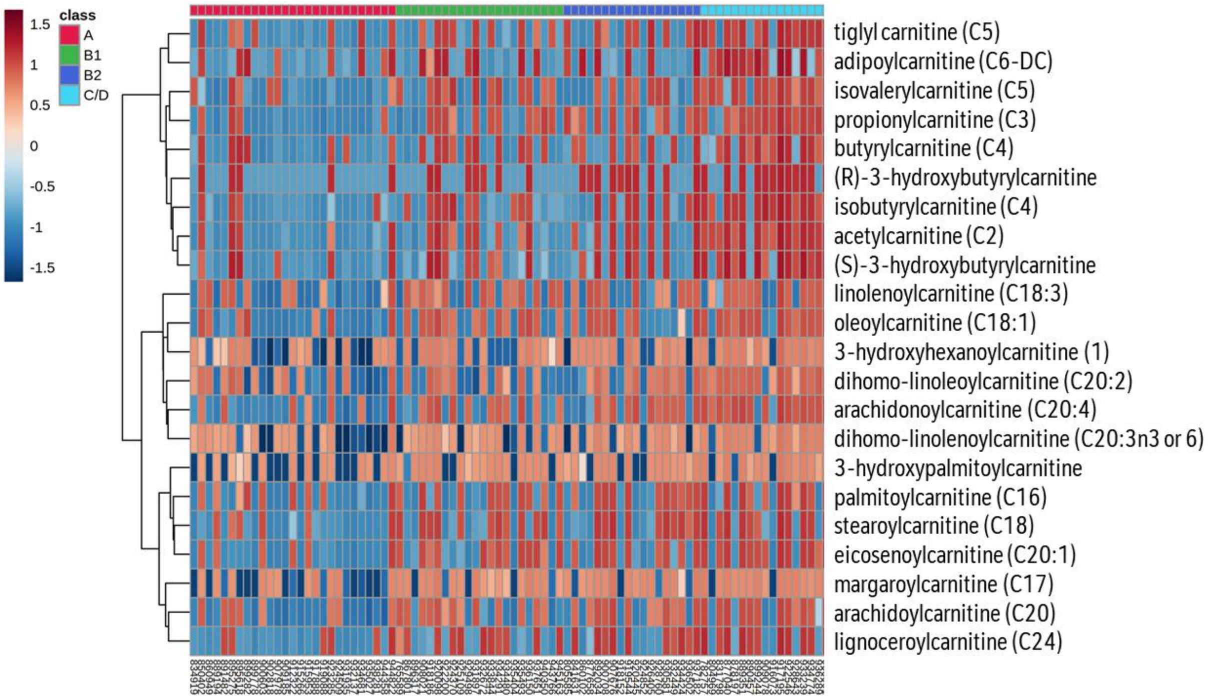

**Figure S5.** Pearson's correlations between aconitate and (A-G) citrate, quinolinate, urea, BHBA, TMAO, 3-MH, and carnosine, and between (H) 3-MH and urea, and (I) 4-guanidinobutanoate and 2-oxoarginine. FDR < 0.0001 in all cases.

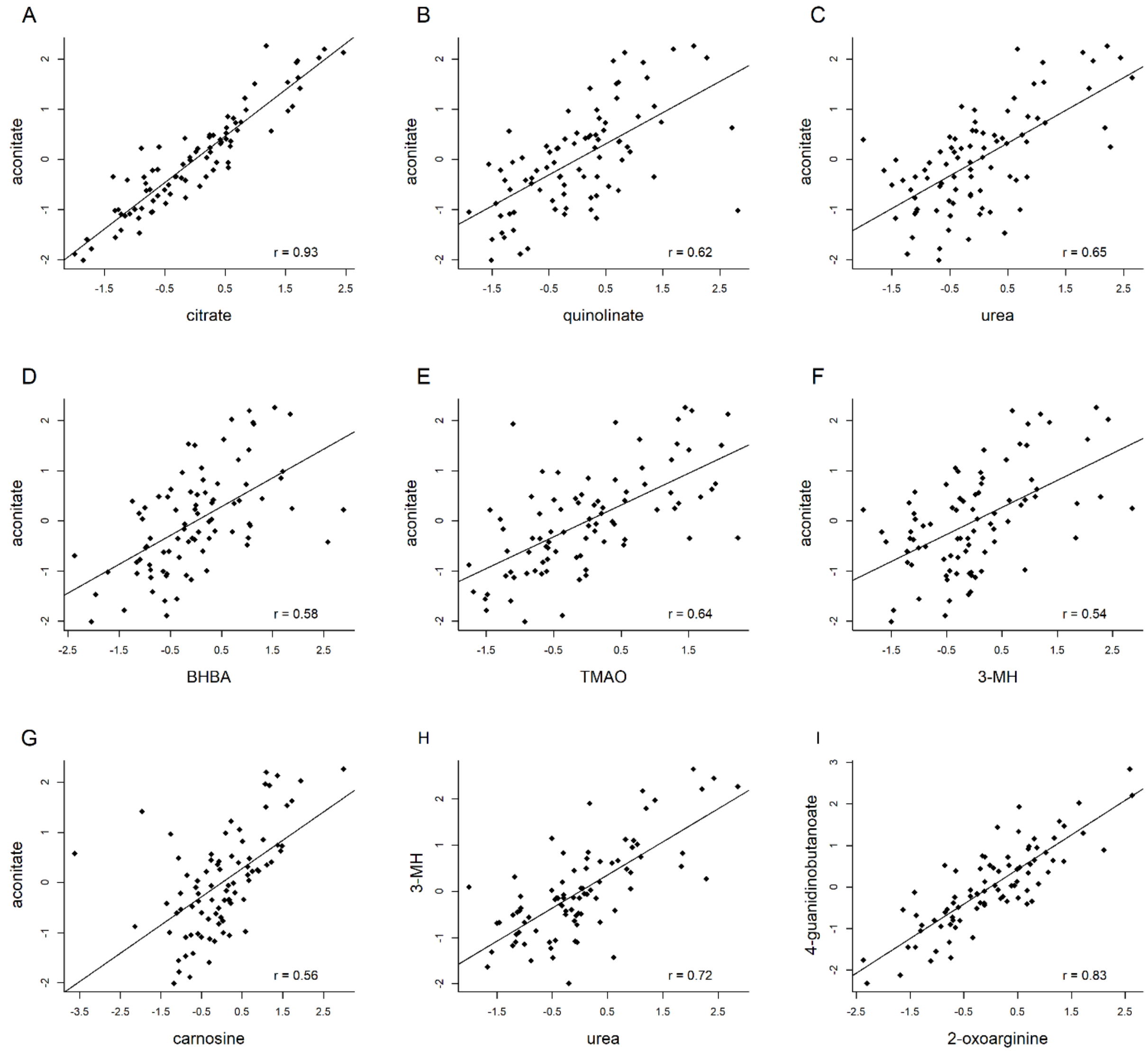

Supplement: Supplementary file 1 — Supplementary Methods Tables S1–S6 Figures S1–S5 [file JAH3-10-e018923-s003.pdf]
